# Systematic Synthesis and Properties Evaluation of Dicationic Ionic Liquids, and a Glance Into a Potential New Field

**DOI:** 10.3389/fchem.2018.00612

**Published:** 2018-12-12

**Authors:** Luca Guglielmero, Andrea Mezzetta, Lorenzo Guazzelli, Christian S. Pomelli, Felicia D'Andrea, Cinzia Chiappe

**Affiliations:** Department of Pharmacy, University of Pisa, Pisa, Italy

**Keywords:** dicationic ionic liquids, thermal analyses, deep eutectic solvents, TGA, DSC

## Abstract

Dicationic ionic liquids (DILs), a subset of the ionic liquid (IL) family, have attracted growing interest in recent years, and the range of applications within which they are investigated is constantly expanding. However, data which allows structure to property correlation of a DIL is still limited, and thus selecting an appropriate salt to address a specific challenge can be problematic. In comparison to traditional ILs, DILs physico-chemical properties can be tuned by changing the length and type of spacer which connects the cationic heads as well as the type of cation. This in turn could give rise to symmetrical or asymmetrical DILs. In this work, a systematic study of a homogeneous class of 12 dibromide DILs and 12 di-carboxylate salts has been performed. The latter class of DILs were also compared to mono cation derivatives. The different traditional exchange methods to prepare carboxylate DILs have been evaluated and an insight into the drawbacks encountered is also presented. Prepared DILs were characterized (NMR, TGA, DSC) allowing the influence of the structure on their thermal stability to be understood. Most DILs were obtained as solid salts after careful drying. For some of these compounds, a new possible application was studied, namely their use as hydrogen bond acceptors (HBA) of deep eutectic mixtures, showing again some significant structural related effects.

## Introduction

Conventional room temperature ionic liquids (ILs) have been extensively studied starting from the beginning of this century by several authors due to their unique physico-chemical properties, which have allowed for their use in numerous applications (Welton, 2018). Low vapor pressure and high thermal stability, associated with possible catalytic actions, which emerge in particular when properly functionalized cations or anions were used (Chiappe and Pomelli, 2014), contributed to their application as innovative green solvents for a plethora of important processes going from (bio)polymer dissolution (Shen et al., 2016; Mezzetta et al., 2017a; Zhang et al., 2017), to chemical reactions (Hallett and Welton, 2011) and separations (Brown et al., 2017). Furthermore, the wide electrochemical window and the high conductivity enabled their use as electrolytes in solar cells and batteries (Watanabe et al., 2017). Generally, they are constituted by an asymmetric organic cation, imidazolium based ILs are the most investigated, associated to a polyatomic inorganic or organic anion (Scalfani et al., 2018). Nonetheless, more recently a new branch of ILs has been proposed: the generally defined dicationic (or geminal) ionic liquids (DILs). These ILs seem to differ from the traditional monocationic ones in many ways offering a growing portfolio of possible applications, ranging from the “classical” use as solvents, catalysts or catalytic supports in organic reactions, to more specific applications as high temperature lubricants/heat transfer fluids, including a variety of roles in analytical sciences (Patil et al., 2016). Further development of these interesting materials in terms of improved performances or new applications are expected, especially if the impact that structural changes can bring upon physical, optical and chemical properties of these unusual material will be understood. Another crucial aspect of ILs is their potential toxicity, which has been scrutinized during the last 10 years. On this regard, the studies performed up to now showed promising toxicity profiles for DILs when compared with the corresponding monocationic ILs (Pretti et al., 2009; Frizzo et al., 2018; Montalbána et al., 2018).

Although a relevant number of DILs has been described in the literature and the number of papers on this topic is rapidly increasing, the relationship between the three-dimensional structure and physicochemical characteristics is, as yet, not well understood.

In analogy with the situation that has characterized simple ILs, the development of DILs chemistry has been much more supported by the creativity of synthetic chemists than by a rational design. Surely, the factors that control the physico-chemical properties of monocationic ILs (MILs), such as the interaction between anions and cations or the structural arrangements of long alkyl chains, play an important role also in the case of dicationic and/or dianionic ILs. However, in this case also the linker(s) between the charged moieties affect the three-dimensional structure of these systems and consequently their principal physical properties, i.e., their ability to solvate dissolved particles, to stabilize transition states or to allow for the formation of polar and apolar domains. Furthermore, asymmetry can be introduced not only by changing the alkyl chains on cations but also introducing two different cationic cores or two different anions. The greater structural variability in DILs is expected to result in an increased tunability and versatility of these salts, when compared to MILs.

Finally, it is noteworthy that the possibility to associate two covalently linked cations to multi-covalently linked anions allows for obtaining the so-called ionic supramolecular polymers by self-assembly, i.e., supramolecular systems with high viscosity and polymer properties arising from the reversible link of small molecules (Craig, 2009). In the last years, starting from the pioneering paper of Wathier and Grinstaff reporting the preparation of organic supramolecular ionic networks by mixing multi-cationic and multi-anionic compounds (Wathier and Grinstaff, 2008), several papers have been published on this topic: for example, using simple proton transfer reactions between citric acid and a diamine, Mecerreyes et al. have prepared (Aboudzadeh et al., 2012a,b, 2013, 2014) protic supramolecular systems based on ionic interactions and, more recently, ionic networks having improved (Aboudzadeh et al., 2015) thermal and chemical stability, employing highly delocalized hydrophobic dianions combined with geminal imidazolium dications. It is to note that this kind of ionic supramolecular organization is generally considered different with respect to the well-known dynamic supramolecular organization of common ionic liquids, which present at the molecular level distinct ionic and non-ionic domains: the two phenomena have probably different time scales.

In order to obtain further information on these ionic media, which have already been used in few applications [e.g., *in situ* formation of DESs in an innovative procedure for the separation of phenols from oils (Youan et al., 2017, 2018), CO_2_ cycloaddition reaction (Liu et al., 2015), elucidation of a reaction mechanism (Bortolini et al., 2014)] or could be used to develop stimuli responsive ionic polymers, a systematic investigation of a homogeneous class of 12 imidazolium based dicationic dibromides and related dicarboxylate salts has been performed. The thermal properties of this latter class of compounds were also compared to mono cation derivatives. The different traditional exchange methods to prepare carboxylate ILs have been evaluated and an insight into the drawbacks encountered is also presented. Prepared salts were characterized (NMR, TGA, DSC) allowing to ascertain the influence of the structure on their thermal behavior/characteristics.

For some of the prepared compounds, a new possible research area was studied, namely their use as hydrogen bond acceptors (HBA) of deep eutectic mixtures. In 2003 Abbott et al. were the first to report the ability of urea to form a deep eutectic solvent (DES) with a quaternary chloride salt (Abbott et al., 2003) Since then, much research has been done in this specific field of solvents which consist of a mixture of two or more components (one of which is a salt) and are characterized by a melting point lower than any of its individual components. The formation of a DES requires the establishment of hydrogen bonds between the salt, in particular its halide anion, and an organic compound acting as hydrogen bond donor (HBD). This determines the formation of a supramolecular complex able to modify the free energy of the solid phase with respect to the liquid one. Although choline chloride is probably the most applied salt to obtain DES, also imidazolium, ammonium and phosphonium salts have been recently used as alternative organic salt components (Florindo et al., 2014).

Thus, considering that all the investigated DILs have a strong hydrogen bond accepting anion, to verify the possibility to transform at least some of the synthesized salts which are solid at room temperature into liquids, we used two reference compounds (**4** and **20**) for the preparation of the corresponding DESs by adding increasing amounts of either glycerol or diethylene glycol.

## Experimental Section

### General Methods

^1^H and ^13^C NMR spectra were recorded with a Bruker Avance II operating at 250.13 and 62.9 MHz at 24°C. The assignments were made with the aid of HSQC and COSY experiments. The first order proton chemical shifts δ are referenced to either residual DMSO (δ_H_ 2.50, δ_C_ 39.5) or D_2_O (δ_H_ 4.79) and *J*-values are given in Hz. The chemical shifts are given in δ and *J*-values are given in Hz. The following abbreviations are used: s = singlet, m = multiplet, bs = broad singlet, t = triplet, bt = broad triplet, q = quartet, qui = quintuplet, sext = sextet.

TLC analyses were performed on Kieselgel 60 F_254_ with detection by UV light (254 nm) and/or with ethanolic 10% phosphomolybdic and heating. All reagents were obtained commercially and used without further purification.

The thermal stability of the synthesized ILs was investigated by thermal gravimetric analysis (TGA), conducted in a TA Instruments Q500 TGA. ILs (15–20 mg) was heated in a platinum crucible as sample holders. First, the heating mode was set to isothermal at 50°C in N_2_ (100 mL/min) for 30 min. Then, IL was heated from 40 to 600°C with a heating rate of 10°C/min under nitrogen (100 mL/min). Mass change was recorded as a function of temperature and time. TGA experiments were carried out in triplicate.

The thermal behavior of the ionic liquids was analyzed by a differential scanning calorimeter (TA DSC, Q250, USA). About 5–10 mg of sample was loaded in hermetic aluminum crucibles. DSC analyses were performed at 10°C/min in nitrogen flow in the temperature range going from −40 to 250°C, as generally reported in the literature for this class of ionic liquids (Lee et al., 2011). In the drying cycle, the sample was heated from 40 to 130°C and maintained at this temperature for 30 min. Then, it was cooled down from 130 to −40°C, at a rate of 10°C/min, and maintained at −40°C for 10 min (cooling run). Finally, the sample was heated to the selected temperature at a rate of 10°C/min to ensure the complete sample melting (heating run). DSC experiments were carried out in duplicate.

Electrospray ionization mass (ESI-MS) spectra were registered on a LCQ Advatage ThermoFinnigan spectrometer equipped with an ion trap analyzer (Thermo Electron Company, San Jose, Ca, USA). Samples were dissolved in methanol (10^−3^ M), spectra were acquired in both negative-ion and positive-ion mode.

#### General Procedure for the Synthesis of 3,3′-(alkane-1,n-diyl)bis(1-alkyl-1H-imidazolium) bromide (1-12)

##### Method A (in toluene)

1,n-Dibromoalkane (58 mmol, 1 equiv) and 10 mL of toluene were added in a three-necked flask. A solution of 1-alkylimidazole (122 mmol, 2.1 equiv) in toluene (10 mL) was added dropwise under magnetic stirring at 0°C. Other 10 mL of toluene were added, and the solution was stirred for 15 min. The reaction mixture was then heated up and stirred at 80°C for 12 h. A white solid precipitation (or liquid separation) was observed after half an hour. Solid precipitates were filtrated under vacuum, washed with toluene (3 × 50 mL), EtOAc (3 × 50 mL) and dried in vacuo, to afford white solids in excellent yields. Liquid products were decanted, washed with toluene and dried in vacuo, to afford colorless liquids in excellent yields.

##### Method B (in 4-methyl-2-pentanone)

Alternatively, compounds **1-12** were prepared according to the general procedure described above, replacing toluene with 4-methyl-2-pentanone (MIBK) as the solvent.

**3,3′-(Propane-1,3-diyl)bis(1-methyl-1*H*-imidazolium) bromide (1)**. The preparation of **1** (97% yield, hygroscopic white solid) was performed according to the general procedure (Method A). ^1^H NMR (D_2_O) δ 8.93 (s, 2H, 2 × H-2), 7.65, 7.40 (2s, each 2H, 2 × H-4, 2 × H-5), 4.35 (t, 4H, *J* = 7.3 Hz, 2 × C*H*_2_N), 3.93 (s, 6H, 2 × NC*H*_3_), 2.56 (qui, 2H, *J* = 7.2 Hz, C*H*_2_);^13^C NMR (D_2_O) δ 136.4 (2 × C-2), 124.1, 122.3 (2 × C-4, 2 × C-5), 46.5 (2 × *C*H_2_N), 36.1 (2 × N*C*H_3_), 30.0 (*C*H_2_). NMR (^1^H, ^13^C) data were in agreement with those reported (Chen et al., 2015; Leclercq and Schmitzer, 2011; Fareghi-Alamdari et al., 2016).

**3,3′-(Butane-1,4-diyl)bis(1-methyl-1*H*-imidazolium) bromide (2)**. The preparation of **2** (97% yield, white solid) was performed according to the general procedure (Method B). ^1^H NMR (D_2_O) δ 8.77 (s, 2H, 2 × H-2), 7.56, 7.45 (2s, each 2H, 2 × H-4, 2 × H-5), 4.26 (bs, 4H, 2 × C*H*_2_N), 3.89 (s, 6H, 2 × NC*H*_3_), 1.94 (bs, 4H, C*H*_2_C*H*_2_); ^13^C NMR (D_2_O) δ 136.2 (2 × C-2), 123.9, 122.3 (2 × C-4, 2 × C-5), 48.9 (2 × *C*H_2_N), 36.0 (2 × N*C*H_3_), 26.4 (*C*H_2_*C*H_2_). NMR (^1^H, ^13^C) data were in agreement with those reported (Nachtigall et al., 2008; Chen et al., 2015).

**3,3′-(Pentane-1,5-diyl)bis(1-methyl-1*H*-imidazolium) bromide (3)**. The preparation of **3** (92% yield, hygroscopic white solid) was performed according to the general procedure (Method A). ^1^H NMR (D_2_O) δ 8.78 (s, 2H, 2 × H-2), 7.88, 7.43 (2s, each 2H, 2 × H-4, 2 × H-5), 4.20 (t, 4H, *J* = 7.1 Hz, 2 × C*H*_2_N), 3.89 (s, 6H, 2 × NC*H*_3_), 1.93 (qui, 4H, *J* = 7.5 Hz, 2 × NCH_2_C*H*_2_), 1.33 (m, 2H, C*H*_2_). ^13^C NMR (D_2_O) δ 136.1 (2 × C-2), 123.8, 122.4 (2 × C-4, 2 × C-5), 49.4 (2 × *C*H_2_N), 36.0 (2 × N*C*H_3_), 28.9 (2 × NCH_2_*C*H_2_), 22.4 (*C*H_2_). NMR (^1^H, ^13^C) data were in agreement with those reported (Nielsen et al., 2006; Chen et al., 2015).

**3,3′-(Hexane-1,5-diyl)bis(1-methyl-1*H*-imidazolium) bromide (4)**. The preparation of **4** (97% yield, white solid) was performed according to the general procedure (Method B). ^1^H NMR (D_2_O) δ 8.62 (s, 2H, 2 × H-2), 7.46, 7.36 (2bs, each 2H, 2 × H-4, 2 × H-5), 4.17 (t, 4H, *J* = 7.1 Hz, 2 × C*H*_2_N), 3.87 (s, 6H, 2 × NC*H*_3_), 1.89 (m, 4H, 2 × NCH_2_C*H*_2_), 1.33 (m, 4H, C*H*_2_C*H*_2_); ^13^C NMR (D_2_O) δ 136.0 (2 × C-2), 123.7, 122.3 (2 × C-4, 2 × C-5), 49.6 (2 × *C*H_2_N), 36.8 (2 × N*C*H_3_), 29.2 (2 × NCH_2_*C*H_2_), 25.0 (*C*H_2_*C*H_2_). NMR (^1^H, ^13^C) data were in agreement with those reported (Bortolini et al., 2017).

**3,3′-(Propane-1,3-diyl)bis(1-butyl-1*H*-imidazolium) bromide (5)**. The preparation of **5** (87% yield, hygroscopic white solid) was performed according to the general procedure (Method B). ^1^H NMR (D_2_O) δ 8.86 (s, 2H, 2 × H-2), 7.52 (bs, 4H, 2 × H-4, 2 × H-5), 4.32 (t, 4H, *J* = 7.2 Hz, 2 × C*H*_2_N), 4.19 (t, 4H, *J* = 7.2 Hz, 2 × C*H*_2_N), 2.56 (qui, 2H, *J* = 7.3 Hz, NCH_2_C*H*_2_CH_2_N), 1.84 (qui, 4H, *J* = 7.3 Hz, NCH_2_C*H*_2_C_2_H_5_), 1.30 (sext, 4H, *J* = 7.5 Hz, 2 × C*H*_2_CH_3_), 0.90 (t, 6H, *J* = 7.3 Hz, 2 × CH_3_); ^13^C NMR (D_2_O) δ 135.3 (2 × C-2), 122.7, 122.1 (2 × C-4, 2 × C-5), 49.4, 46.4 (each 2 × *C*H_2_N), 31.1 (2 × NCH_2_*C*H_2_C_2_H_5_), 29.6 (NCH_2_*C*H_2_CH_2_N), 18.7 (2 × *C*H_2_CH_3_), 12.6 (2 × *C*H_3_). NMR (^1^H) data were in agreement with those reported (Lee et al., 2010; Priede et al., 2014).

**3,3′-(Butane-1,4-diyl)bis(1-butyl-1*H*-imidazolium) bromide (6)**. The preparation of **6** (99% yield, highly viscous colorless liquid) was performed according to the general procedure (Method B). ^1^H NMR (D_2_O) δ 8.82 (s, 2H, 2 × H-2), 7.48 (bs, 4H, 2 × H-4, 2 × H-5), 4.30-4.20 (m, 8H, 4 × C*H*_2_N), 1.90-1.75 (m, 8H, 4 × NCH_2_C*H*_2_), 1.38 (m, 4H, 2 × CH_3_C*H*_2_), 0.85 (t, 6H, *J* = 7.3 Hz, 2 × CH_3_); ^13^C NMR (D_2_O) δ 135.1 (2 × C-2), 122.4, 122.0 (2 × C-4, 2 × C-5), 49.3 (2 × *C*H_2_N), 48.6 (2 × *C*H_2_N), 31.1, 26.2 (2 × NCH_2_*C*H_2_C_2_H_5_, 2 × NCH_2_*(CH*_2_*)*_2_CH_2_N), 18.7 (2 × *CH*_2_CH_3_), 12.6 (2 × *C*H_3_). NMR (^1^H, ^13^C) data were in agreement with those reported (Mata et al., 2004).

**3,3′-(Pentane-1,5-diyl)bis(1-butyl-1*H*-imidazolium) bromide (7)**. The preparation of **7** (99% yield, highly viscous colorless liquid) was performed according to the general procedure (Method B). ^1^H NMR (D_2_O) δ 8.84 (s, 2H, 2 × H-2), 7.52 (bs, 4H, 2 × H-4, 2 × H-5), 4.25-4.18 (m, 8H, 4 × C*H*_2_N), 2.00-1.80 (m, 8H, 4 × C*H*_2_CH_2_N), 1.38-1.23 (m, 6H, N(CH_2_)_2_*C*H_2_(CH_2_)_2_N, 2 × *CH*_2_CH_3_), 0.91 (t, 6H, *J* = 7.3 Hz, 2 × CH_3_); ^13^C NMR (D_2_O) δ 134.9 (2 × C-2), 122.3, 122.2 (2 × C-4, 2 × C-5), 49.3 (2 × *C*H_2_N), 49.1 (2 × *C*H_2_N), 31.2 (2 × NCH_2_*C*H_2_), 28.6 (2 × NCH_2_*CH*_2_), 22.1 (*CH*_2_), 18.7 (2 × *CH*_2_CH_3_), 12.6 (2 × *C*H_3_). ESI-MS: positive mode, [C_5_(BIM)_2_-H]^+^ *m/z* 317.22 (25%), [C_5_(BIM)_2_/Br]^+^ *m/z* 397.12 (100%) 397.15 (100%); negative mode, Br^−^*m/z* 79.05 (100%) and 81.06 (100%), ([C_5_(BIM)_2_]_2_/5Br)^−^ *m/z* 1035.13 (100% and all the other isotopic peaks), ([C_5_(BIM)_2_]_3_/7Br)^−^ *m/z* 1515.30 (100% and all the other isotopic peaks).

**3,3′-(Hexane-1,6-diyl)bis(1-butyl-1*H*-imidazolium) bromide (8)**. The preparation of **8** (93% yield, hygroscopic white solid) was performed according to the general procedure (Method B). ^1^H NMR (D_2_O) δ 8.77 (s, 2H, 2 × H-2), 7.47 (bs, 4H, 2 × H-4, 2 × H-5), 4.21-4.14 (m, 8H, 4 × C*H*_2_N), 1.89-1.77 (m, 8H, 4 × C*H*_2_CH_2_N), 1.36-1.21 (m, 8H, 2 × N(CH_2_)_2_*C*H_2_, 2 × *CH*_2_CH_3_), 0.89 (t, 6H, *J* = 7.3 Hz, 2 × CH_3_); ^13^C NMR (D_2_O) δ 135.0 (2 × C-2), 122.3, 122.1 (2 × C-4, 2 × C-5), 49.3 (4 × *C*H_2_N), 31.1 (2 × NCH_2_*C*H_2_), 29.0 (2 × NCH_2_*CH*_2_), 24.7 (*C*H_2_*C*H_2_), 18.6 (2 × *CH*_2_CH_3_), 12.5 (2 × *C*H_3_). NMR (^1^H) data were in agreement with those reported (Yang et al., 2014).

**3,3′-(Propane-1,3-diyl)bis(1-hexyl-1*H*-imidazolium) bromide (9)**. The preparation of **9** (94% yield, white solid) was performed according to the general procedure (Method B). ^1^H NMR (D_2_O) δ 8.87 (s, 2H, 2 × H-2), 7.50 (s, 4H, 2 × H-4, 2 × H-5), 4.31 (m, 4H, 2 × C*H*_2_N), 4.16 (m, 4H, 2 × C*H*_2_N), 2.52 (m, 2H, C*H*_2_), 1.83 (m, 8H, 2 × C_3_H_9_C*H*_2_CH_2_N), 1.25 [s, 12H, 2 × Me(C*H*_2_)_3_], 0.80 (bt, 6H, 2 × C*H*_3_); ^13^C NMR (D_2_O) δ 135.2 (2 × C-2), 122.6, 122.0 (2 × C-4, 2 × C-5), 49.6, 46.2 (each 2 × *C*H_2_N), 30.1 (2 × C_3_H_9_*C*H_2_CH_2_N), 29.6 (*C*H_2_), 28.9, 24.8, 21.6 (2 × Me(*C*H_2_)_3_),13.1(2 × *C*H_3_).

**3,3′-(butane-1,4-diyl)bis(1-hexyl-1*H*-imidazolium) bromide (10)**. The preparation of **10** (98% yield, viscous colorless liquid) was performed according to the general procedure (Method B). ^1^H NMR (D_2_O) δ 8.79 (s, 2H, 2 × H-2), 7.48 (m, 4H, 2 × H-4, 2 × H-5), 4.23-4.14 (m, 8H, 4 × C*H*_2_N), 1.85 (m, 8H, 4 × NCH_2_C*H*_2_), 1.24 [s, 12H, 2 × Me(C*H*_2_)_3_], 0.80 (bt, 6H, 2 × C*H*_3_); ^13^C NMR (D_2_O) δ 135.1 (2 × C-2), 122.5, 122.0 (2 × C-4, 2 × C-5), 49.5, 48.6 (each 2 × *C*H_2_N), 30.1 (2 × C_3_H_9_*C*H_2_CH_2_N), 28.9, 24.8, 21.6 (2 × Me(*C*H_2_)_3_), 26.1 (*C*H_2_*C*H_2_), 13.0 (2 × *C*H_3_). ESI-MS: positive mode, [C_4_(HIM)_2_-H]^+^ *m/z* 359.36 (20%), [C_4_(HIM)_2_/Br]^+^ *m/z* 439.25 (100%) 441.29 (100%); negative mode, Br^−^*m/z* 79.05 (100%) and 81.06 (100%), ([C_4_(HIM)_2_]_2_/5Br)^−^ *m/z* 1119.33 (100% and all the other isotopic peaks), ([C_5_(BIM)_2_]_3_/7Br)^−^ *m/z* 1641.30 (100% and all the other isotopic peaks).

**3,3′-(pentane-1,5-diyl)bis(1-hexyl-1*H*-imidazolum) bromide (11)**. The preparation of **11** (96% yield, white solid) was performed according to the general procedure (Method B). ^1^H NMR (D_2_O) δ 8.82 (s, 2H, 2 × H-2), 7.49 (s, 4H, 2 × H-4, 2 × H-5), 4.21-4.14 (m, 8H, 4 × C*H*_2_N), 1.90-1.80 (m, 8H, 4 × NCH_2_C*H*_2_), 1.24 [s, 14H, 2 × Me(C*H*_2_)_3_, C*H*_2_], 0.80 (bt, 6H, 2 × C*H*_3_); ^13^C NMR (D_2_O) δ 135.0 (2 × C-2), 122.3, 122.2 (2 × C-4, 2 × C-5), 49.4, 49.0 (each 2 × *C*H_2_N), 30.1, 29.0 (each 2 × *C*H_2_CH_2_N), 28.5, 24.8, 21.6 (2 × Me(*C*H_2_)_3_), 22.0 (*C*H_2_), 13.0 (2 × *C*H_3_). ESI-MS: positive mode, [C_5_(HIM)_2_-H]^+^ *m/z* 373.12 (20%), [C_5_(HIM)_2_/Br]^+^ *m/z* 453.06 (100%) and 455.05 (100%); negative mode, Br^−^*m/z* 79 (100%) and 81.06 (100%), ([C_5_(HIM)_2_]_2_/5Br)^−^ *m/z* 1149.13 (100% and all the other isotopic peaks), ([C_5_(HIM)_2_]_3_/7Br)^−^ *m/z* 1682.90 (100% and all the other isotopic peaks).

**3,3′-(Hexane-1,6-diyl)bis(1-hexyl-1*H*-imidazolium) bromide (12)**. The preparation of **12** (98% yield, viscous colorless liquid) was performed according to the general procedure (Method B). ^1^H NMR (D_2_O) δ 8.82 (s, 2H, 2 × H-2), 7.50 (m, 4H, 2 × H-4, 2 × H-5), 4.19 (t, 8H, *J* = xx Hz, 2 × C*H*_2_N), 1.84 (m, 8H, 4 × NCH_2_C*H*_2_), 1.30-1.24 [m, 16H, 2 × Me(C*H*_2_)_3_, C*H*_2_C*H*_2_], 0.80 (bt, 6H, 2 × C*H*_3_);^13^C NMR (D_2_O) δ 134.9 (2 × C-2), 122.2 (2 × C-4, 2 × C-5), 49.4, 46.2 (each 2 × *C*H_2_N), 30.1 (2 × C_3_H_9_*C*H_2_CH_2_N), 28.9, 24.8, 21.6 (2 × Me(*C*H_2_)_3_), 24.6 (*C*H_2_*,C*H_2_), 13.1 (2 × *C*H_3_). ESI-MS: positive mode, [C_6_(HIM)_2_-H]^+^ *m/z* 387.32 (20%), [C_6_(HIM)_2_/Br]^+^ *m/z* 467.22 (100%) 469.35 (100%); negative mode, Br^−^*m/z* 79.05 (100%) and 81.06 (100%), ([C_6_(HIM)_2_]_2_/5Br)^−^ *m/z* 1175.02 (100% and all the other isotopic peaks), ([C_6_(HIM)_2_]_3_/7Br)^−^ *m/z* 1723.30 (100% and all the other isotopic peaks).

#### General Procedure for the Synthesis of 3,3′-(alkane-1,n-diyl)bis(1-alkyl-1H-imidazolium) carboxylate (13-24)

##### Method A (with silver hydroxide)

To a NaOH aqueous solution (10% w/w), AgNO_3_ (1 equiv) was added and the mixture was stirred at room temperature in the dark, for 10 min. The resulting brown mixture was filtered under vacuum over through a Teflon filter and left drying in the dark. Selected 3,3′-(alkane-1,n-diyl)bis(1-alkyl-1*H*-imidazol-3ium) bromide (1 equiv) was dissolved in water to form a 10% w/w solution. AgOH (1.1 equiv) was added to the bromide solution and the resulting suspension was left under stirring at room temperature in the dark. After 12 h, the mixture was filtered over Celite. To the filtrated solution, opportune carboxylic acid (1 equiv) was added and the mixture was stirred overnight. In case a precipitate was observed, the suspension was filtered over Celite. Evaporation of solvent under reduced pressure afforded hygroscopic white solids or highly viscous yellowish liquids.

##### Method B (with Ag_2_CO_3_)

To a stirred solution of the carboxylic acid (malonic acid, succinic acid or glutaric acid) in water (20 mL/g), commercial Ag_2_CO_3_ (1.02 equiv) was added slowly, in the dark. At the end of the effervescence, opportune 3,3′-(alkane-1,n-diyl)bis(1-alkyl-1*H*-imidazolium) bromide (1.0 equiv) was added and the reaction mixture was stirring at room temperature in the dark overnight. The resulting yellowish suspension was filtered over Celite and the clear solution was concentrated under diminished pressure affording either hygroscopic white solids or highly viscous yellowish liquids.

##### Method C (with ion exchange resin)

A suspension of Amberlite IRA400 ion exchange resin in 500 mL of an aqueous NaOH solution (4% w/w) was stirred at room temperature for 2 days. The pretreated resin was packed into a 3.5 cm diameter column and washed with water until clear washing waters were obtained. Three hundred milliliters of NaOH solution (4% w/w) were passed through the column and recycled for three times. The same procedure was repeated with other 300, 200, and 200 mL of NaOH 4% w/w. The column was then washed with water until neutrality of washing waters.

Selected 3,3′-(alkane-1,n-diyl)bis(1-alkyl-1*H*-imidazolium) bromide (18.6 mmol) was dissolved in 7:3 (v/v) CH_3_OH-water (100 mL) and the solution was passed through the column. The eluted was recovered and passed through the column until absence of halogens (this step was repeated five times). The hydroxyl-bromide substitution was followed by checking the presence of bromide in the eluted solution using pure silver nitrate (AgBr test). The column was then washed with 200 mL of CH_3_OH/water solution and the eluted was combined with the previous 100 mL of opportune 3′-(alkane-1,n-diyl)bis(1-alkyl-1*H*-imidazolium) hydroxide solution. A solution of the proper carboxylic acid (18.6 mmol) in 7:3 (v/v) CH_3_OH-water (250 mL) was added under stirring to the prepared 3,3′-(alkane-1,n-diyl)bis(1-alkyl-1*H*-imidazolium) hydroxide solution. After 1 h at room temperature, the solvent was evaporated under reduced pressure affording, after drying in vacuo, hygroscopic white solids or highly viscous yellowish liquids.

**3,3′-(Propane-1,3-diyl)bis(1-methyl-1*H*-imidazolium) malonate (13)**. The preparation of **13** (95% yield, hygroscopic white solid) was performed according to the general procedure (Method C). ^1^H NMR (D_2_O) δ 8.76 (s, 2H, 2 × H-2), 7.46, 7.42 (2s, each 2H, 2 × H-4, 2 × H-5), 4.27 (t, 4H, *J* = 7.3 Hz, 2 × C*H*_2_N), 3.85 (s, 6H, 2 × NC*H*_3_), 2.47 (m, 4H, C*H*_2_, CH_2_CO); ^13^C NMR (D_2_O) δ 177.1 (2 × C = O), 136.0 (2 × C-2), 123.9, 122.1 (2 × C-4, 2 × C-5), 47.3 (2 × *C*H_2_N, *C*H_2_CO), 35.8 (2 × N*C*H_3_), 29.7 (*C*H_2_). ESI-MS: positive mode, [C_3_(MIM)_2_-H]^+^ *m/z* 204.96 (70%), [C_3_(MIM)_2_/Mal + H]^+^ *m/z* 309.10 (30%), ([C_3_(MIM)_2_/Mal]_2_ + H)^+^ *m/z* 617.10 (100%); negative mode, (Mal + H)^−^*m/z* 102.86 (100%), [C_3_(MIM)_2_]/3(Mal + H)^−^*m/z* 515.20 (70%), 2[C_3_(MIM)_2_]/[Mal+ 3(Mal + H)]^−^*m/z* 823.20 (30%), 3[C_3_(MIM)_2_]/[Mal+ 5(Mal + H)]^−^ *m/z* 1235.40 (100%).

**3,3′-(Butane-1,4-diyl)bis(1-methyl-1*H*-imidazolium) malonate (14)**. The preparation of **14** (94% yield, hygroscopic white solid) was performed according to the general procedure (Method C). ^1^H NMR (D_2_O) δ 8.69 (s, 2H, 2 × H-2), 7.25, 7.10 (2bs, each 2H, 2 × H-4, 2 × H-5), 3.95 (bs, 4H, 2 × C*H*_2_N), 3.61 (s, 6H, 2 × NC*H*_3_), 2.81 (s, 2H, C*H*_2_CO), 1.58 (m, 4H, C*H*_2_C*H*_2_); ^13^C NMR (D_2_O) δ 176.9 (2 × C = O), 135.9 (2 × C-2), 123.6, 121.9 (2 × C-4, 2 × C-5), 48.5 (2 × *C*H_2_N), 47.7 (*C*H_2_CO), 35.6 (2 × N*C*H_3_), 26.0 (*C*H_2_*C*H_2_). ESI-MS: positive mode, [C_4_(MIM)_2_-H]^+^ *m/z* 219.00 (70%), [C_4_(MIM)_2_/Mal + H]^+^ *m/z* 323.04 (30%), ([C_4_(MIM)_2_/Mal]_2_ + H)^+^ *m/z* 645.20 (100%); negative mode, (Mal + H)^−^*m/z* 102.86 (100%), [C_4_(MIM)_2_]/3(Mal + H)^−^*m/z* 528.90 (70%), 2[C_4_(MIM)_2_]/[Mal+ 3(Mal + H)]^−^*m/z* 850.87 (40%), 3[C_4_(MIM)_2_]/[Mal+ 5(Mal + H)]^−^ *m/z* 1277.10 (100%).

**3,3′-(Pentane-1,5-diyl)bis(1-methyl-1*H*-imidazolium) malonate (15)**. The preparation of **15** (95% yield, hygroscopic white solid) was performed according to the general procedure (Method C). ^1^H NMR (D_2_O) δ 8.68 (s, 2H, 2 × H-2), 7.41, 7.39 (2bs, each 2H, 2 × H-4, 2 × H-5), 4.15 (t, 4H, *J* = 7.2 Hz, 2 × C*H*_2_N), 3.84 (s, 6H, 2 × NC*H*_3_), 3.06 (s, 2H, C*H*_2_CO), 1.87 (qui, 4H, *J* = 7.5 Hz, 2 × NCH_2_C*H*_2_), 1.27 (m, 2H, C*H*_2_). ^13^C NMR (D_2_O) δ 177.1 (2 × C = O), 136.0 (2 × C-2), 123.6, 122.2 (2 × C-4, 2 × C-5), 49.2 (2 × *C*H_2_N), 47.4 (*C*H_2_CO), 35.7 (2 × N*C*H_3_), 28.7 (2 × NCH_2_*C*H_2_), 22.2 (*C*H_2_). ESI-MS: positive mode, [C_5_(MIM)_2_-H]^+^ *m/z* 232.90 (100%), [C_5_(MIM)_2_/Mal + H]^+^ *m/z* 336.82 (50%), ([C_5_(MIM)_2_/Mal]_2_ + H)^+^ *m/z* 672.91 (20%); negative mode, (Mal + H)^−^*m/z* 102.86 (100%), [C_5_(MIM)_2_]/3(Mal + H)^−^*m/z* 542.67 (70%), 2[C_5_(MIM)_2_]/[Mal+ 3(Mal + H)]^−^*m/z* 850.87 (40%), 3[C_5_(MIM)_2_]/[Mal+ 5(Mal + H)]^−^ *m/z* 1318.69 (65%).

**3,3′-(Hexane-1,6-diyl)bis(1-methyl-1*H*-imidazolium) malonate (16)**. The preparation of **16** (94% yield, hygroscopic white solid) was performed according to the general procedure (Method C). ^1^H NMR (D_2_O) δ 8.67 (s, 2H, 2 × H-2), 7.41, 7.38 (2m, each 2H, 2 × H-4, 2 × H-5), 4.13 (t, 4H, *J* = 7.1 Hz, 2 × C*H*_2_N), 3.83 (s, 6H, 2 × NC*H*_3_), 3.04 (s, 2H, C*H*_2_CO), 1.81 (m, 4H, 2 × NCH_2_C*H*_2_), 1.38 (m, 4H, C*H*_2_C*H*_2_); ^13^C NMR (D_2_O) δ 176.5 (2 × C = O), 135.3 (2 × C-2), 123.0, 122.6 (2 × C-4, 2 × C-5), 48.8 (2 × *C*H_2_N), 47.2 (2 × *C*H_2_CO), 35.0 (2 × N*C*H_3_), 28.5 (2 × NCH_2_*C*H_2_), 24.3 (*C*H_2_*C*H_2_).). ESI-MS: positive mode, [C_6_(MIM)_2_-H]^+^ *m/z* 246.99 (100%), [C_6_(MIM)_2_/Mal + H]^+^ *m/z* 350.88 (20%); negative mode, (Mal + H)^−^*m/z* 102.96 (100%), [C_6_(MIM)_2_]/3(Mal + H)^−^*m/z* 557.01 (70%), 2[C_6_(MIM)_2_]/[Mal+ 3(Mal + H)]^−^ *m/z* 907.21 (30%).

**3,3′-(Propane-1,4-diyl)bis(1-methyl-1*H*-imidazolium) succinate (17)**. The preparation of **17** (97% yield, hygroscopic white solid) was performed according to the general procedure (Method C). ^1^H NMR (D_2_O) δ 8.75 (s, 2H, 2 × H-2), 7.46, 7.41 (2s, each 2H, 2 × H-4, 2 × H-5), 4.25 (t, 4H, *J* = 7.2 Hz, 2 × C*H*_2_N), 3.83 (s, 6H, 2 × NC*H*_3_), 2.45 (m, 2H, C*H*_2_), 2.26 (m, 4H, C*H*_2_, 2 × C*H*_2_CO); ^13^C NMR (D_2_O) δ 181.2 (2 × C = O), 136.2 (2 × C-2), 124.0, 122.1 (2 × C-4, 2 × C-5), 46.3 (2 × *C*H_2_N), 35.8 (2 × N*C*H_3_), 34.2 (2 × *C*H_2_CO), 29.7 (*C*H_2_). ESI-MS: positive mode, [C_3_(MIM)_2_-H]^+^ *m/z* 205.15 (70%), [C_3_(MIM)_2_/Succ + H]^+^ *m/z* 323.30 (100%), ([C_3_(MIM)_2_/Succ]_2_ + H)^+^ *m/z* 645.34 (70%); negative mode, (Succ + H)^−^*m/z* 117.00 (70%), [C_3_(MIM)_2_]/3(Succ + H)^−^*m/z* 557.10 (100%), 2[C_3_(MIM)_2_]/[Succ + 3(Succ + H)]^−^*m/z* 879.45 (80%).

**3,3′-(Butane-1,4-diyl)bis(1-methyl-1*H*-imidazolium) succinate (18)**. The preparation of **18** (98% yield, hygroscopic white solid) was performed according to the general procedure (Method C). ^1^H NMR (D_2_O) δ 8.45 (s, 2H, 2 × H-2), 7.18, 7.16 (2bs, each 2H, 2 × H-4, 2 × H-5), 3.96 (bs, 4H, 2 × C*H*_2_N), 3.60 (s, 6H, 2 × NC*H*_3_), 2.10 (s, 4H, 2 × C*H*_2_CO), 1.61 (m, 4H, C*H*_2_C*H*_2_); ^13^C NMR (D_2_O) δ 181.7 (2 × C = O), 135.9 (2 × C-2), 123.6, 121.9 (2 × C-4, 2 × C-5), 48.6 (2 × *C*H_2_N), 35.6 (2 × N*C*H_3_), 33.8 (2 × *C*H_2_CO), 26.0 (*C*H_2_*C*H_2_). ESI-MS: positive mode, [C_4_(MIM)_2_-H]^+^ *m/z* 218.80 (55%), [C_4_(MIM)_2_/Succ + H]^+^ *m/z* 336.79 (100%), ([C_4_(MIM)_2_/Succ]_2_ + H)^+^ *m/z* 672.89, (70%); negative mode, (Succ + H)^−^*m/z* 117.00 (78%), [C_4_(MIM)_2_]/3(Succ + H)^−^*m/z* 571.12 (40%), 2[C_4_(MIM)_2_]/[Succ + 3(Succ + H)]^−^ *m/z* 907.02 (100%).

**3,3′-(Pentane-1,5-diyl)bis(1-methyl-1*H*-imidazolium) succinate (19)**. The preparation of **19** (97% yield, hygroscopic white solid) was performed according to the general procedure (Method C). ^1^H NMR (D_2_O) δ 8.54 (s, 2H, 2 × H-2), 7.28, 7.25 (2bs, each 2H, 2 × H-4, 2 × H-5), 4.00 (t, 4H, *J* = 7.2 Hz, 2 × C*H*_2_N), 3.70 (s, 6H, 2 × NC*H*_3_), 2.19 (s, 4H, 2 × C*H*_2_CO), 1.73 (qui, 4H, *J* = 7.5 Hz, 2 × NCH_2_C*H*_2_), 1.23 (m, 2H, C*H*_2_). ^13^C NMR (D_2_O) δ 181.8 (2 × C = O), 135.9 (2 × C-2), 123.6, 122.1 (2 × C-4, 2 × C-5), 49.2 (2 × *C*H_2_N), 35.7 (2 × N*C*H_3_), 34.0 (2 × *C*H_2_CO), 28.7 (2 × NCH_2_*C*H_2_), 22.2 (*C*H_2_). ESI-MS: positive mode, [C_5_(MIM)_2_-H]^+^ *m/z* 233.15 (65%), [C_5_(MIM)_2_/Succ + H]^+^ *m/z* 351.17 (100%), ([C_5_(MIM)_2_/Succ]_2_ + H)^+^ *m/z* 701.56 (70%); negative mode, (Succ + H)^−^*m/z* 117.00 (70%), [C_5_(MIM)_2_]/3(Succ + H)^−^*m/z* 585.32 (100%), 2[C_5_(MIM)_2_]/[Succ + 3(Succ + H)]^−^ *m/z* 935.40 (80%).

**3,3′-(Hexane-1,6-diyl)bis(1-methyl-1*H*-imidazolium) succinate (20)**. The preparation of **20** (hygroscopic white solid) was performed according to the general procedure (Method A: 61% yield; Method B: 98% yield; Method C: 92% yield). ^1^H NMR (D_2_O) δ 8.66 (s, 2H, 2 × H-2), 7.49, 7.37 (2m, each 2H, 2 × H-4, 2 × H-5), 4.13 (t, 4H, *J* = 7.0 Hz, 2 × C*H*_2_N), 3.83 (s, 6H, 2 × NC*H*_3_), 2.24 (s, 4H, 2 × C*H*_2_CO), 1.82 (m, 4H, 2 × NCH_2_C*H*_2_), 1.28 (m, 4H, C*H*_2_C*H*_2_); ^13^C NMR (D_2_O) δ 181.4 (2 × C = O), 135.3 (2 × C-2), 123.0, 121.6 (2 × C-4, 2 × C-5), 48.8 (2 × *C*H_2_N), 35.1 (2 × N*C*H_3_), 33.6 (2 × *C*H_2_CO), 28.5 (2 × NCH_2_*C*H_2_), 24.3 (*C*H_2_*C*H_2_). ESI-MS: positive mode, [C_6_(MIM)_2_-H]^+^ *m/z* 247.30 (65%), [C_6_(MIM)_2_/Succ + H]^+^ *m/z* 365.35 (100%), ([C_6_(MIM)_2_/Succ]_2_ + H)^+^ *m/z* 729.50 (70%); negative mode, (Succ + H)^−^*m/z* 117.00 (70%), [C_6_(MIM)_2_]/3(Succ + H)^−^*m/z* 599.38 (100%), 2[C_6_(MIM)_2_]/[Succ + 3(Succ + H)]^−^*m/z* 963.50 (70%).

**3,3′-(Propane-1,3-diyl)bis(1-methyl-1*H*-imidazolium) glutarate (21)**. The preparation of **21** (99% yield, hygroscopic white solid) was performed according to the general procedure (Method C). ^1^H NMR (D_2_O) δ 8.75 (s, 2H, 2 × H-2), 7.47, 7.44 (2s, each 2H, 2 × H-4, 2 × H-5), 4.28 (t, 4H, *J* = 7.3 Hz, 2 × C*H*_2_N), 3.86 (s, 6H, 2 × NC*H*_3_), 2.48 (qui, 2H, *J* = 7.2 Hz, C*H*_2_), 2.16 (t, 4H, *J* = 7.5 Hz, 2 × C*H*_2_CO), 1.74 (bq, 2H, *J* = 7.6 Hz, C*H*_2_CH_2_CO); ^13^C NMR (D_2_O) δ 183.2 (2 × C = O), 136.2 (2 × C-2), 124.0, 122.1 (2 × C-4, 2 × C-5), 46.1 (2 × *C*H_2_N), 37.3 (2 × *C*H_2_CO), 35.8 (2 × N*C*H_3_), 29.8 (*C*H_2_), 23.0 (*C*H_2_CH_2_CO). ESI-MS: positive mode, [C_3_(MIM)_2_-H]^+^ *m/z* 204.82 (40%), ([C_3_(MIM)_2_/Glut]_2_ + H)^+^ *m/z* 673.02 (100%); negative mode, (Glut + H)^−^*m/z* 131.02 (100%), [C_3_(MIM)_2_]/3(Glut + H)^−^*m/z* 599.17 (70%), 2[C_3_(MIM)_2_]/[Glut+ 3(Glut + H)]^−^ *m/z* 935.19 (70%).

**3,3′-(Butane-1,4-diyl)bis(1-methyl-1*H*-imidazolium) glutarate (22)**. The preparation of **22** (98% yield, hygroscopic white solid) was performed according to the general procedure (Method C). ^1^H NMR (D_2_O) δ 8.69 (s, 2H, 2 × H-2), 7.41, 7.39 (2bs, each 2H, 2 × H-4, 2 × H-5), 4.19 (bs, 4H, 2 × C*H*_2_N), 3.83 (s, 6H, 2 × NC*H*_3_), 2.09 (t, 4H, *J* = 7.4 Hz, 2 × C*H*_2_CO), 1.84 (m, 4H, C*H*_2_C*H*_2_), 1.72 (s, 2H, C*H*_2_CH_2_CO); ^13^C NMR (D_2_O) δ 182.3 (2 × C = O), 135.4 (2 × C-2), 123.2, 121.5 (2 × C-4, 2 × C-5), 48.1 (2 × *C*H_2_N), 36.8 (2 × *C*H_2_CO), 35.6 (2 × N*C*H_3_), 25.6 (*C*H_2_*C*H_2_), 22.5 (*C*H_2_CH_2_CO). ESI-MS: positive mode, [C_4_(MIM)_2_-H]^+^ *m/z* 218.95 (40%), ([C_4_(MIM)_2_/Glut]_2_ + H)^+^ *m/z* 701.05 (100%); negative mode, (Glut + H)^−^*m/z* 131.00 (100%), [C_4_(MIM)_2_]/3(Glut + H)^−^*m/z* 613.15 (70%), 2[C_4_(MIM)_2_]/[Glut+ 3(Glut + H)]^−^ *m/z* 963.50 (60%).

**3,3′-(Pentane-1,5-diyl)bis(1-methyl-1*H*-imidazolium) glutarate (23)**. The preparation of **23** (97% yield, hygroscopic white solid) was performed according to the general procedure (Method C). ^1^H NMR (D_2_O) δ 8.67 (s, 2H, 2 × H-2), 7.41, 7.38 (2bs, each 2H, 2 × H-4, 2 × H-5), 4.14 (t, 4H, *J* = 7.2 Hz, 2 × C*H*_2_N), 3.83 (s, 6H, 2 × NC*H*_3_), 2.11 (t, 4H, *J* = 7.4 Hz, 2 × C*H*_2_CO), 1.86 (m, 4H, 2 × NCH_2_C*H*_2_), 1.70 (m, 2H, C*H*_2_CH_2_CO), 1.23 (m, 2H, C*H*_2_); ^13^C NMR (D_2_O) δ 182.8 (2 × C = O), 135.9 (2 × C-2), 123.6, 122.2 (2 × C-4, 2 × C-5), 49.2 (2 × *C*H_2_N), 37.3 (2 × *C*H_2_CO), 35.7 (2 × N*C*H_3_), 28.8 (2 × NCH_2_*C*H_2_), 23.0 (*C*H_2_CH_2_CO), 22.2 (*C*H_2_). ESI-MS: positive mode, [C_5_(MIM)_2_-H]^+^ *m/z* 233.10 (40%), ([C_5_(MIM)_2_/Glut]_2_ + H)^+^ *m/z* 729.01 (100%); negative mode, (Glut + H)^−^*m/z* 131.00 (100%), [C_5_(MIM)_2_]/3(Glut + H)^−^*m/z* 626.99 (70%), 2[C_5_(MIM)_2_]/[Glut+ 3(Glut + H)]^−^ *m/z* 990.98 (50%).

**3,3′-(Hexane-1,6-diyl)bis(1-methyl-1*H*-imidazolium) glutarate (24)**. The preparation of **24** (98% yield, hygroscopic white solid) was performed according to the general procedure (Method C). ^1^H NMR (D_2_O) δ 8.66 (s, 2H, 2 × H-2), 7.41, 7.38 (2m, each 2H, 2 × H-4, 2 × H-5), 4.12 (t, 4H, *J* = 7.1 Hz, 2 × C*H*_2_N), 3.83 (s, 6H, 2 × NC*H*_3_), 2.12 (t, 4H, *J* = 7.4 Hz, 2 × C*H*_2_CO), 1.83-1.67 (m, 6H, 2 × NCH_2_C*H*_2_, C*H*_2_CH_2_CO), 1.29 (m, 4H, C*H*_2_C*H*_2_); ^13^C NMR (D_2_O) δ 182.1 (2 × C = O), 135.3 (2 × C-2), 123.0, 121.6 (2 × C-4, 2 × C-5), 48.8 (2 × *C*H_2_N), 36.7 (2 × *C*H_2_CO), 35.1 (2 × N*C*H_3_), 28.5 (2 × NCH_2_*C*H_2_), 24.3 (*C*H_2_*C*H_2_), 22.4 (*C*H_2_CH_2_CO).

**3,3′-(Butane-1,4-diyl)bis(1-butyl-1*H*-imidazolium) succinate (25)**. The preparation of **25** (99% yield, hygroscopic white solid) was performed according to the general procedure (Method C). ^1^H NMR (D_2_O) δ 8.77 (s, 2H, 2 × H-2), 7.46, 7.44 (2s, each 2H, 2 × H-4, 2 × H-5), 4.19-4.11 (m, 8H, 4 × C*H*_2_N), 2.34 (s, 4H, 2 × C*H*_2_CO), 1.85-1.76 (m, 8H, 4 × C*H*_2_CH_2_N), 1.25 (sext, 4H, *J* = 7.6 Hz, 2 × C*H*_2_CH_3_), 0.85 (t, 6H, *J* = 7.3 Hz, 2 × CH_3_); ^13^C NMR (D_2_O) δ 181.8 (2 × C = O), 135.1 (2 × C-2), 122.5, 122.0 (2 × C-4, 2 × C-5), 49.2, 48.6 (each 2 × *C*H_2_N), 33.9 (2 × *C*H_2_CO), 31.1 (2 × NCH_2_*C*H_2_C_2_H_5_), 26.1 (NCH_2_*C*H_2_CH_2_N), 18.6 (2 × *C*H_2_CH_3_), 12.5 (2 × *C*H_3_). ESI-MS: positive mode, [C_4_(BIM)_2_-H]^+^ *m/z* 303.20 (70%), [C_4_(BIM)_2_/Succ + H]^+^ *m/z* 421.30 (100%), ([C_4_(BIM)_2_/Succ]_2_ + H)^+^ *m/z* 841.55 (70%); negative mode, (Succ + H)^−^*m/z* 117.00 (80%), [C_4_(BIM)_2_]/3(Succ + H)^−^*m/z* 655.27 (100%), 2[C_4_(HIM)_2_]/[Succ + 3(Succ + H)]^−^*m/z* 1075.40 (85%).

**3,3′-(Butane-1,4-diyl)bis(1-hexyl-1*H*-imidazolium) succinate (26)**. The preparation of **26** (99% yield, hygroscopic white solid) was performed according to the general procedure (Method C). ^1^H NMR (250 MHz, D_2_O) δ ^1^H NMR (D_2_O) δ 8.76 (s, 2H, 2 × H-2), 7.48 (m, 4H, 2 × H-4, 2 × H-5), 4.20-4.10 (m, 8H, 4 × C*H*_2_N), 2.33 (s, 4H, 2 × C*H*_2_CO), 1.83 (m, 8H, 4 × NCH_2_C*H*_2_), 1.21 [s, 12H, 2 × Me(C*H*_2_)_3_], 0.77 (bt, 6H, 2 × C*H*_3_); ^13^C NMR (D_2_O) δ 181.9 (2 × C = O), 135.2 (2 × C-2), 122.6, 122.1 (2 × C-4, 2 × C-5), 49.6, 48.6 (each 2 × *C*H_2_N), 34.0 (2 × *C*H_2_CO), 30.1 (2 × C_3_H_9_*C*H_2_CH_2_N), 29.0, 24.9, 21.7 (2 × Me(*C*H_2_)_3_), 26.2 (*C*H_2_*C*H_2_), 13.1 (2 × *C*H_3_). ESI-MS: positive mode, [C_4_(HIM)_2_-H]^+^ *m/z* 359.03 (90%), [C_4_(HIM)_2_/Succ + H]^+^ *m/z* 476.89 (100%), ([C_4_(HIM)_2_/Succ]_2_ + H)^+^ *m/z* 953.10 (80%); negative mode, (Succ + H)^−^*m/z* 117.00 (70%), [C_4_(HIM)_2_]/3(Succ + H)^−^*m/z* 711.33 (100%), 2[C_4_(HIM)_2_]/[Succ + 3(Succ + H)]^−^ *m/z* 1187.16 (80%).

**General Procedure for the Synthesis of 3-butyl-1-methyl-1*H*-imidazolium carboxylate (27-29)**. To a commercial methanolic solution of 3-butyl-1-methyl-1*H*-imidazolium methylcarbonate (Proionic, 34% w/w), the opportune carboxylic acid (1 equiv) was added. The resulting mixture was stirred at room temperature for 1 h and the solvent was evaporated under reduced pressure to afford a yellow oil in quantitative yield.

**3-Butyl-1-methyl-1*H*-imidazolium malonate (27)**. The preparation of **27** (99% yield, viscous yellow oil) was performed according to the general procedure. ^1^H NMR (D_2_O) δ 8.57 (s, 2H, 2 × H-2), 7.31, 7.27 (2s, each 2H, 2 × H-4, 2 × H-5), 4.03 (t, 4H, *J* = 7.7 Hz, 2 × C*H*_2_N), 3.73 (s, 6H, 2 × NC*H*_3_), 2.94 (bs, 2H, C*H*_2_CO), 1.71-1.65 (m, 4H, 2 × C*H*_2_CH_2_N), 1.19-1.10 (m, 4H, 2 × C*H*_2_CH_3_), 0.75 (t, 6H, *J* = 7.2 Hz, 2 × CH_3_); ^13^C NMR (D_2_O) δ 177.0 (2 × C = O), 135.9 (2 × C-2), 123.5, 122.2 (2 × C-4, 2 × C-5), 49.3 (2 × *C*H_2_N), 47.5 (*C*H_2_CO), 35.6 (2 × N*C*H_3_), 31.3 (2 × NCH_2_*C*H_2_), 18.8 (2 × *C*H_2_CH_3_), 12.7 (2 × *C*H_3_). ESI-MS: positive mode, [BMIM]^+^ *m/z* 139.04 (100%); negative mode, (Mal + H)^−^*m/z* 102.86 (20%), [BMIM/2(Mal + H)]^−^ *m/z* 344.77 (100%).

**3-Butyl-1-methyl-1*H*-imidazolium succinate (28)**. The preparation of **28** (99% yield, viscous yellow oil) was performed according to the general procedure. ^1^H NMR (D_2_O) δ 8.68 (s, 2H, 2 × H-2), 7.42, 7.37 (2s, each 2H, 2 × H-4, 2 × H-5), 4.11 (bt, 4H, 2 × C*H*_2_N), 3.83 (s, 6H, 2 × NC*H*_3_), 2.29 (bs, 4H, 2 × C*H*_2_CO), 1.83-1.71 (m, 4H, 2 × C*H*_2_CH_2_N), 1.28-1.19 (m, 4H, 2 × C*H*_2_CH_3_), 0.85 (bt, 6H, 2 × CH_3_); ^13^C NMR (D_2_O) δ 181.7 (2 × C = O), 135.7 (2 × C-2), 123.3, 122.0 (2 × C-4, 2 × C-5), 49.1 (2 × *C*H_2_N), 35.5(2 × N*C*H_3_), 34.2 (2 × *C*H_2_CO), 31.1 (2 × NCH_2_*C*H_2_), 18.6 (2 × *C*H_2_CH_3_), 12.5 (2 × *C*H_3_). ESI-MS: positive mode, [BMIM]^+^ *m/z* 139.04 (100%); negative mode, (Succ + H)^−^*m/z* 117.00 (50%), [BMIM/2(Succ + H)]^−^ *m/z* 373.00 (100%).

**3-Butyl-1-methyl-1*H*-imidazolium glutarate (29)**. The preparation of **29** (99% yield, viscous yellow oil) was performed according to the general procedure. ^1^H NMR (D_2_O) δ 8.65 (s, 2H, 2 × H-2), 7.40, 7.35 (2s, each 2H, 2 × H-4, 2 × H-5), 4.11 (t, 4H, *J* = 7.0 Hz, 2 × C*H*_2_N), 3.81 (s, 6H, 2 × NC*H*_3_), 2.08 (t, 4H, *J* = 7.5 Hz, 2 × C*H*_2_CO), 1.81-1.62 (m, 6H, 2 × NCH_2_C*H*_2_, C*H*_2_CH_2_CO), 1.30-1.15 (m, 4H, 2 × C*H*_2_CH_3_), 0.83 (t, 6H, *J* = 7.3 Hz, 2 × CH_3_); ^13^C NMR (D_2_O) δ 182.7 (2 × C = O), 135.6 (2 × C-2), 123.2, 122.0 (2 × C-4, 2 × C-5), 49.1 (2 × *C*H_2_N), 37.2 (2 × *C*H_2_CO), 35.5 (2 × N*C*H_3_), 31.0 (2 × NCH_2_*C*H_2_), 22.8 (*C*H_2_CH_2_CO), 18.5 (2 × *C*H_2_CH_3_), 12.4 (2 × *C*H_3_). ESI-MS: positive mode, [BMIM]^+^ *m/z* 139.02 (100%); negative mode, (Glut + H)^−^*m/z* 131.00 (50%), [BMIM/2(Glut + H)]^−^ *m/z* 400.98 (100%).

**3,3′-(Hexane-1,6-diyl)bis(1-methyl-1H-imidazolium) bromide (4) diethylenglycol mixture 1:6**. Derivative **4** (1 equiv) and diethylenglycol (6 equiv), previously dried at rotary evaporator at 80°C for 2 h, were mixed and stirred at 80°C under argon for 1 h. ^1^H NMR (neat at 23°C) δ 8.63 (s, 2H, 2 × H-2), 7.27, 7.18 (2bs, each 2H, 2 × H-4, 2 × H-5), 4.11 (bs, 12H, 12 × OH), 3.71 (bs, 4H, 2 × C*H*_2_N), 3.40 (s, 6H, 2 × NC*H*_3_), 2.97 (bs, 24H, 12 × C*H*_2_O), 2.92 (bs, 24H, 12 × C*H*_2_OH), 1.29 (bs, 4H, 2 × NCH_2_C*H*_2_), 0.76 (bs, 4H, C*H*_2_C*H*_2_); ^13^C NMR (neat at 23°C) δ 135.7 (2 × C-2), 122.8, 121.5 (2 × C-4, 2 × C-5), 71.3 (12 × *C*H_2_O), 59.8 (12 × *C*H_2_OH), 48.2 (2 × *C*H_2_N), 35.3 (2 × N*C*H_3_), 28.6 (2 × NCH_2_*C*H_2_), 24.2 (*C*H_2_*C*H_2_).

**3,3′-(Hexane-1,6-diyl)bis(1-methyl-1H-imidazolium) succinate (20) diethylenglycol mixture 1:2**. Derivative **20** (1 equiv) and diethylenglycol (2 equiv), previously dried at rotary evaporator at 80°C for 2 h, were mixed and stirred at 80°C under argon for 1 h. ^1^H NMR (neat at 63°C) δ 9.21 (s, 2H, 2 × H-2), 7.41, 7.30 (2bs, each 2H, 2 × H-4, 2 × H-5), 6.04 (bs, 4H, 4 × OH), 3.80 (bs, 4H, 2 × C*H*_2_N), 3.80 (s, 6H, 2 × NC*H*_3_), 2.89 (bs, 8H, 4 × C*H*_2_O), 2.80 (bs, 8H, 4 × C*H*_2_OH), 1.69 (s, 4H, 2 × C*H*_2_CO), 1.21 (bs, 4H, 2 × NCH_2_C*H*_2_), 0.68 (bs, 4H, C*H*_2_C*H*_2_); ^13^C NMR (neat at 63°C) δ 177.4 (2 × C = O), 136.5 (2 × C-2), 122.7, 121.5 (2 × C-4, 2 × C-5), 71.3 (4 × *C*H_2_O), 59.3 (4 × *C*H_2_OH), 47.7 (2 × *C*H_2_N), 34.5 (2 × *C*H_2_CO, 2 × N*C*H_3_), 28.3 (2 × NCH_2_*C*H_2_), 23.8 (*C*H_2_*C*H_2_).

**3,3′-(Hexane-1,6-diyl)bis(1-methyl-1H-imidazolium) bromide (4) glycerol mixture 1:3**. Derivative **4** (1 equiv) and glycerol (3 equiv), previously dried at rotary evaporator at 80°C for 2 h, were mixed and stirred at 80°C under argon for 1 h. ^1^H NMR (neat at 23°C) δ 8.34 (s, 2H, 2 × H-2), 6.98, 6.90 (2bs, each 2H, 2 × H-4, 2 × H-5), 3.75-3.60 (m, 9H, 9 × OH), 3.41 (bt, 4H, 2 × C*H*_2_N), 3.13 (s, 6H, 2 × NC*H*_3_), 2.79 (bs, 3H, 12 × C*H*OH), 2.62 (bs, 12H, 6 × C*H*_2_OH), 0.98 (bs, 4H, 2 × NCH_2_C*H*_2_), 0.44 (bs, 4H, C*H*_2_C*H*_2_); ^13^C NMR (neat at 23°C) δ 135.4 (2 × C-2), 122.5, 121.2 (2 × C-4, 2 × C-5), 71.4 (3 × *C*HO), 61.9 (6 × *C*H_2_OH), 48.1 (2 × *C*H_2_N), 35.4 (2 × N*C*H_3_), 28.3 (2 × NCH_2_*C*H_2_), 23.9 (*C*H_2_*C*H_2_).

**3,3′-(Hexane-1,6-diyl)bis(1-methyl-1H-imidazolium) succinate (20) glycerol mixture 1:2**. Derivative **20** (1 equiv) and glycerol (2 equiv), previously dried at rotary evaporator at 80°C for 2 h, were mixed and stirred at 80°C under argon for 1 h. ^1^H NMR (neat at 63°C) δ 8.75 (s, 2H, 2 × H-2), 7.14, 7.05 (2bs, each 2H, 2 × H-4, 2 × H-5), 5.25 (bs, 6H, 6 × OH), 3.53 (bs, 4H, 2 × C*H*_2_N), 3.24 (s, 6H, 2 × NC*H*_3_), 2.84-2.60 (m, 10H, 2 × C*H*O, 4 × C*H*_2_OH), 1.58 (s, 4H, 2 × C*H*_2_CO), 1.11 (bs, 4H, 2 × NCH_2_C*H*_2_), 0.59 (bs, 4H, C*H*_2_C*H*_2_); ^13^C NMR (neat at 63°C) δ 177.8 (2 × C = O), 135.9 (2 × C-2), 122.6, 121.3 (2 × C-4, 2 × C-5), 71.3 (2 × *C*HOH), 62.2 (4 × *C*H_2_OH), 47.8 (2 × *C*H_2_N), 34.5 (2 × N*C*H_3_), 33.8 (2 × *C*H_2_CO), 28.1 (2 × NCH_2_*C*H_2_), 23.8 (*C*H_2_*C*H_2_).

## Results and Discussion

### Synthesis and Characterization of the Investigated DILs

**Figure 1:**
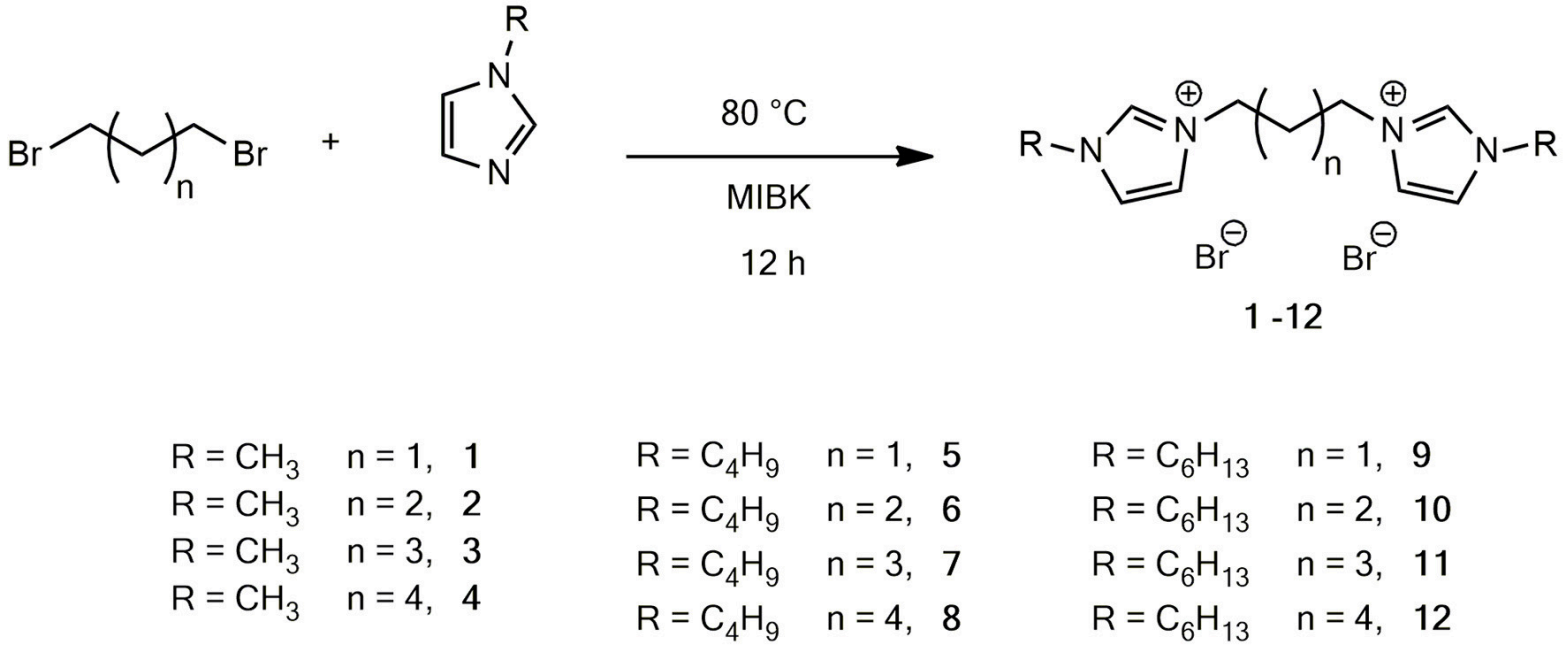
Structures of the synthetized dibromide DILs.

Initially, a series of imidazolium bromide based DILs, four of which have never been reported before (**7, 10-12**), varying both in the alkyl linker length (from C3 to C6) and in the length of the substituent on the imidazolium ring (1-methyl-, 1-butyl and 1-hexyl), were synthesized (Figure 1).

The generally used Menshutkin reaction between the selected 1-alkylimidazole and the proper 1,*n*-dibromoalkane was carried out in toluene in the first experiments, following previously reported procedures (Kishore and Das, 2012). However, toluene was subsequently substituted with 4-methyl-2-pentanone (MIBK), following a protocol already tested in the case of monocationic ILs (Chiappe et al., 2016), without observing any drop of yield. MIBK has lower aquatic and air impacts than toluene according to the GSK's Solvent Sustainability Guide (Alder et al., 2016).

**Figure 2:**
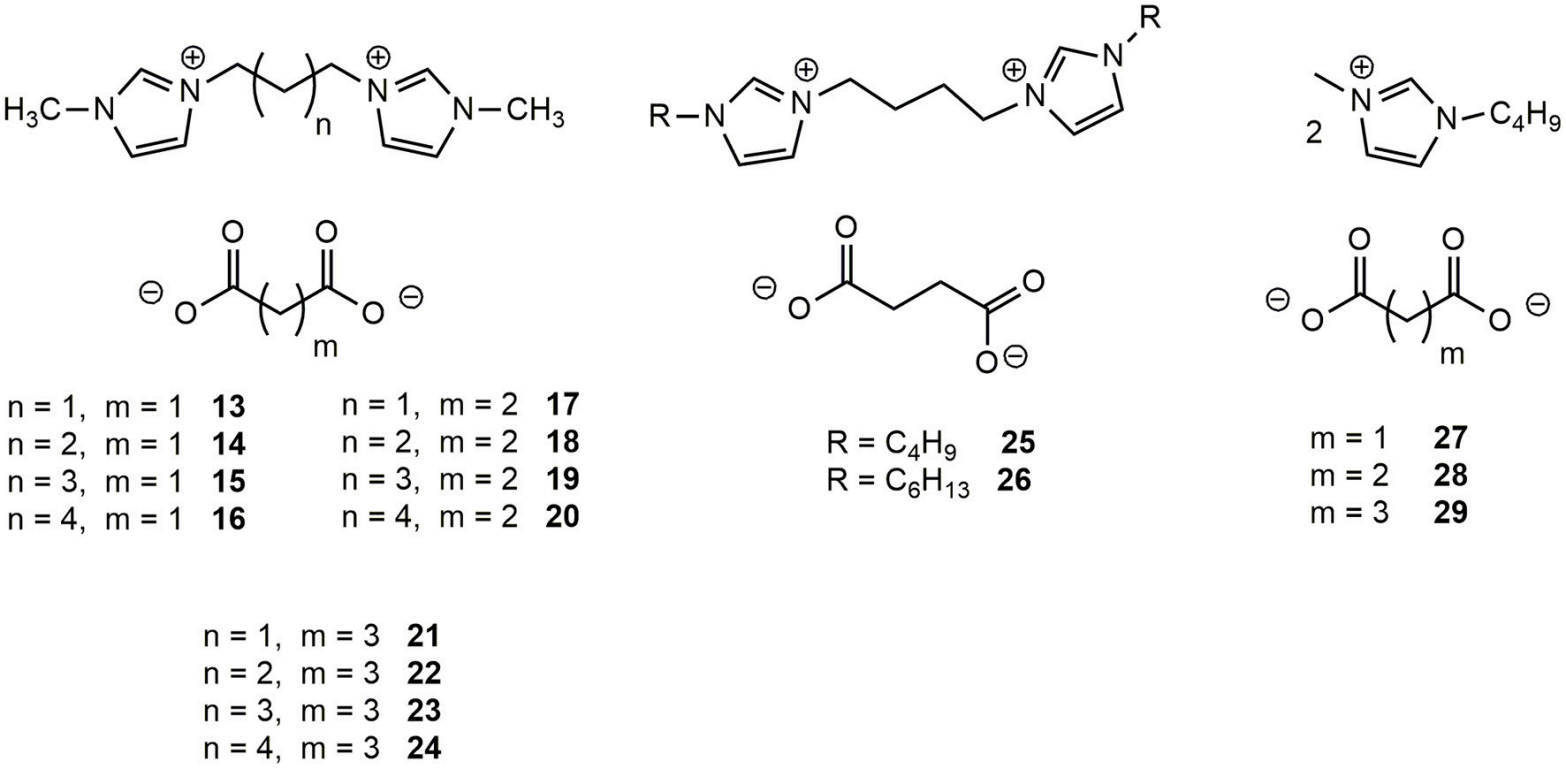
Structures of the synthetized dicarboxylated dicationic and monocationic ILs.

The four 1-methylimidazolium bromides DILs **1**-**4**, differing for the linker length, were transformed into the corresponding dicarboxylate salts (Figure 2), namely malonate, succinate, and glutarate whereas salts **6** and **10** were converted into the corresponding succinates DILs (**25** and **26**). 1-Methyl-3-butylimidazolium methyl carbonate was also used as starting material to prepare the corresponding malonate, succinate and glutarate salts (**27-29**) following known procedures (Mezzetta et al., 2017b). With the exception of **24** (Bortolini et al., 2014), all the prepared dicarboxylate salts have not yet been reported in the Literature.

**Figure 3:**
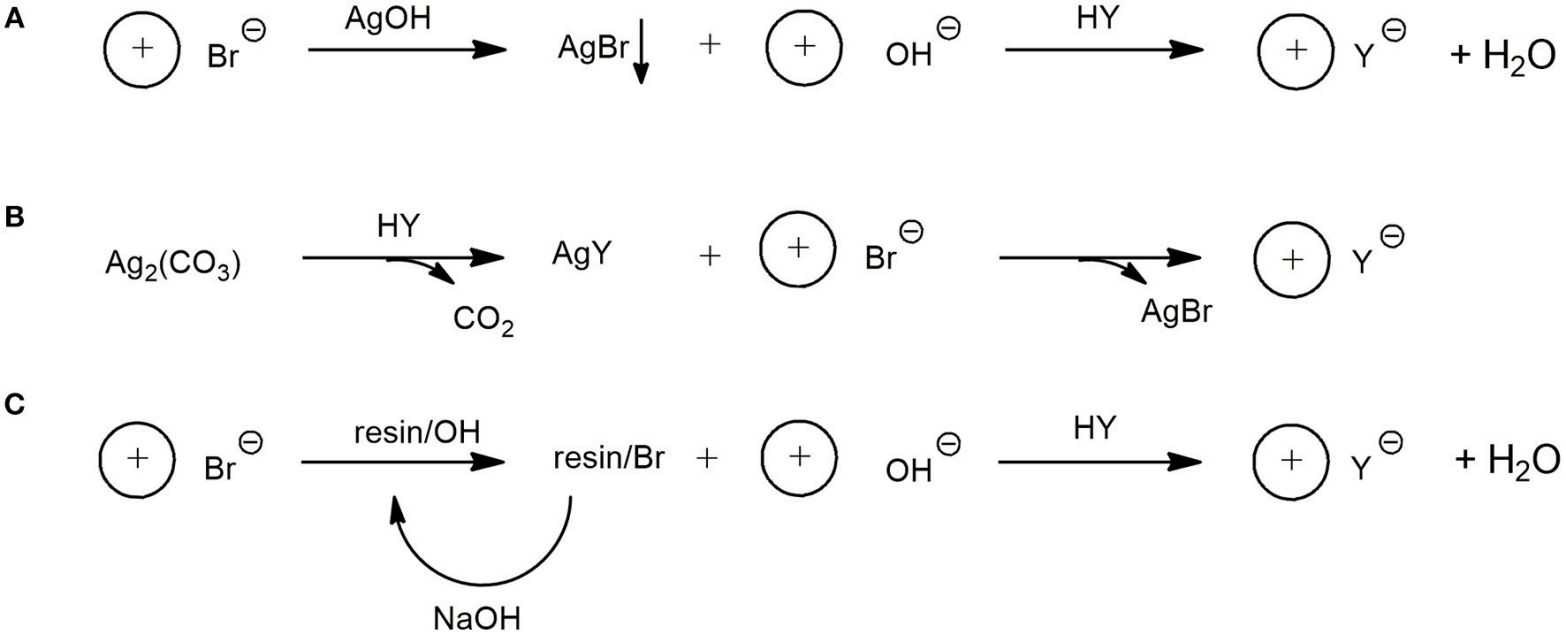
Synthetic procedures for the preparation of dicarboxylate DILs using: AgOH **(A)**; Ag_2_CO_3_ **(B)**; Ion Exchange Resin **(C)**.

To ascertain the most convenient approach for the preparation of the target dicarboxylates in high yield and degree of purity, three different largely used synthetic procedures (Figure 3) were tested employing **4** as the substrate for the synthesis of **20**.

In particular, as schematically shown in Figure 3 path A, AgOH was employed for the bromide-hydroxide anion exchange. Subsequently, after removal of AgBr by filtration, the target product was obtained by addition of a stoichiometric amount of succinic acid to the resulting clear water solution. Unfortunately, poor results in terms of recovered material and purity were reached through this approach, probably due to the formation in the relatively strong basic medium of silver imidazole carbene complexes.

Since this approach is normally used successfully to prepare water soluble ILs starting from bromide or chloride based ILs, the chelating properties of the bis-imidazole systems toward silver could probably explain the different behavior of mono and dicationic ILs in this reaction. The carbene species formed from dicationic DILs can find a stabilization despite the aqueous medium in the formation of the chelated silver complex. Thus, to avoid imidazolium ring deprotonation the less basic silver carbonate was employed instead of silver hydroxide (path B). After the formation of the dicarboxylate silver salt by reacting silver carbonate with a stochiometric amount of succinic acid, the metathesis reaction was performed by adding the bromide DIL, and the desired product was obtained in practically quantitative yield.

Finally, an ion exchange resin-based procedure was tried (path C). The resin, in its hydroxy form, was used to perform the complete bromide-hydroxy substitution and the desired product, after succinic acid addition, was obtained also in this case in quantitative yield.

This latter procedure was applied to obtain the dicarboxylate based DILs **13**-**26**. At least for an application at lab scale, the relatively high cost of the silver carbonate makes the more time requiring exchange resin-based procedure competitive.

### Thermal Analysis

**Figure 4:**
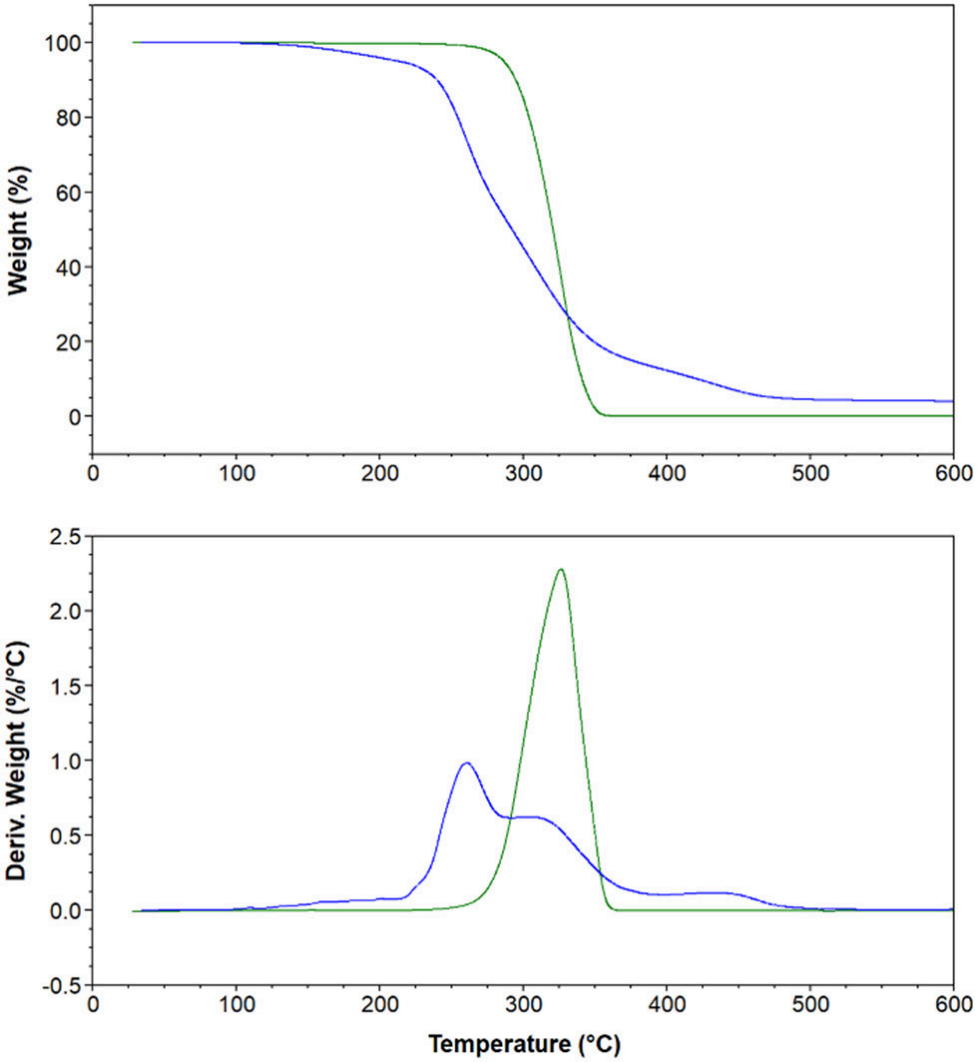
Thermal gravimetric analysis (top) and derivative curves (bottom) of **4** [C_6_(MIM)_2_/2Br] (Green) and **20** [C_6_(MIM)_2_/Succinate] (Blue).

**Figure 5:**
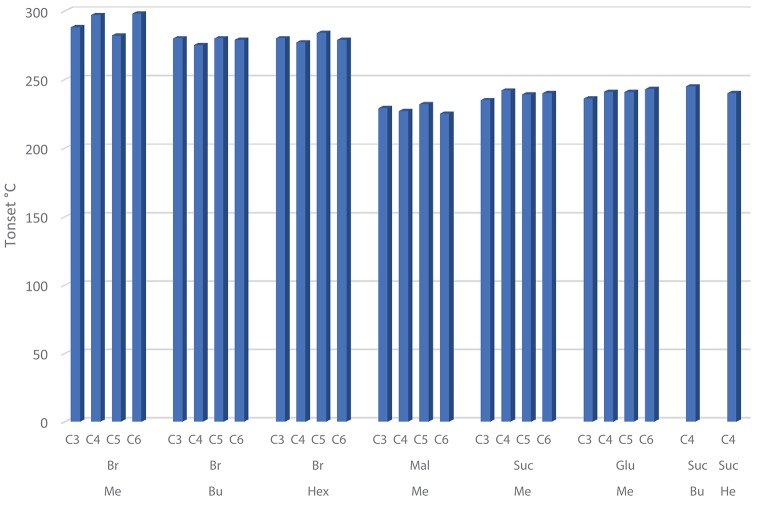
T_*onset*_ values plotted against the linker alkyl chain length, grouped on the basis of the imidazole substituent (methyl, butyl or hexyl) for both bromide (Br) and dicarboxylate (Mal, Suc, Glu) DILs.

The short-term stability of all synthesized DILs was determined by using dynamic TGA. Thus, DILs were exposed to a continuous linear increase of temperature and the mass loss was monitored. Furthermore, before the analysis all samples, although previously accurately dried, were subjected to a further drying cycle consisting in an isothermal run at 50°C for 30 min. Dynamic TGA curves were registered also for DILs previously investigated to avoid any effect related to the experimental conditions. From the thermographs, three characteristic temperatures of the short-term thermal stability were evaluated for each investigated DIL, T_*start*_, T_*onset*_, and T_*peak*_ (Table 1). It should be noted that when the decomposition process occurs in more steps and the corresponding DTG curves are therefore characterized by two or more peaks, only the decomposition temperature corresponding to the highest peak was considered. Figure 4 shows the dynamic curves for two typical DILs, while all the others are reported as Supplementary Information. For a more effective comparison, Figure 5 shows the T_*onset*_ of all investigated DILs.

**Table 1:** T_*start*_, T_*onset*_, and T_peak_ of the investigated ionic liquids (DILs and ILs) measured under a nitrogen atmosphere and with a heating rate of 10°C/min.

		**T*_***start***_* (**°**C)**	**T*_***onset***_* (**°**C)**	**T*_***peak***_* (**°**C)**	**Lit**.
**1**	C_3_(MIM)_2_/2Br	271	288	308	T_start_ = 283°C^a^
					290°C^b^
					T*_*onset*_* = 311°C^a^
**2**	C_4_(MIM)_2_/2Br	285	297	332	T_start_ = 318°C^c^
					281°C^a^
					250°C^d^
					T*_*onset*_* = 301°C^a^
**3**	C_5_(MIM)_2_/2Br	272	282	303	
**4**	C_6_(MIM)_2_/2Br	287	298	326	
**5**	C_3_(BIM)_2_/2Br	251	280	315	T*_*start*_* = 250°C^b^
**6**	C_4_(BIM)_2_/2Br	260	275	304	
**7**	C_5_(BIM)_2_/2Br	263	280	310	
**8**	C_6_(BIM)_2_/2Br	264	279	307	T*_*onset*_* = 297°C^e^
**9**	C_3_(HIM)_2_/2Br	265	280	309	T*_*start*_* = 242°C^f^
**10**	C_4_(HIM)_2_/2Br	260	277	308	
**11**	C_5_(HIM)_2_/2Br	259	284	314	
**12**	C_6_(HIM)_2_/2Br	260	279	309	
**13**	C_3_(MIM)_2_/Mal	190	229	245	
**14**	C_4_(MIM)_2_/Mal	144	227	253	
**15**	C_5_(MIM)_2_/Mal	175	232	255	
**16**	C_6_(MIM)_2_/Mal	201	225	256	
**17**	C_3_(MIM)_2_/Succ	166	235	261	
**18**	C_4_(MIM)_2_/Succ	156	242	262	
**25**	C_4_(BIM)_2_/Succ	174	245	280	
**26**	C_4_(HIM)_2_/Succ	208	240	270	
**19**	C_5_(MIM)_2_/Succ	187	239	260	
**20**	C_6_(MIM)_2_/Succ	215	240	261	
**21**	C_3_(MIM)_2_/Glut	143	236	252	
**22**	C_4_(MIM)_2_/Glut	209	241	261	
**23**	C_5_(MIM)_2_/Glut	226	241	261	
**24**	C_6_(MIM)_2_/Glut	238	243	262	
**27**	BMIM/ Mal	170	195	214	
**28**	BMIM/Succ	208	223	257	
**29**	BMIM/Glut	162	221	246	

a *Youan et al. (2017)*.

b *Priede et al. (2014)*.

c *Zhang et al. (2018)*.

d *Lee et al. (2010)*.

e *Yang et al. (2014)*.

f *Lee et al. (2011)*.

All bromide based DILs display a similar thermal stability, with the onset degradation temperature ranging between 275°C, **6** [C_4_(MIM)_2_/2Br], and 298°C, **4** [C_6_(MIM)_2_/2Br]. Thus, in agreement with the Cao and Mu (Cao and Mu, 2014) classification, identifying five different classes of ionic liquids on the basis of their *T*_onest_, these DILs can be defined as “less stable.” The five classes are indeed the following: least stable (200°C < T_*onest*_ < 250°C), less stable (250°C < T_*onest*_ < 300°C), moderate (300°C < T_*onest*_ < 350°C), more stable (350°C < T_*onest*_ < 400°C), most stable (400°C < T_*onest*_ < 450°C). Furthermore, based on the T_*start*_, T_*peak*_, and T_*onset*_ values, the thermal stability of the investigated DILs having bromide as counteranion results affected (moderately) by the length of the lateral alkyl chain: DILs based on the 1-methylimidazolium cation are more stable than the corresponding salts based on 1-n-butyl and 1-n-hexyl imidazolium cations. The linker length instead appears practically unable to influence the thermal stability, only in the case of the 1-methyl imidazolium DIL subfamily, a small difference in the degradation temperatures between the alkyl linkers with an even or odd number of carbon atoms can be observed. DILs with an even number of carbon atoms show a degradation temperature higher than subsequent odd ones. This peculiar behavior was not observed in DILs with butyl or hexyl alkyl chain.

On the other hand, all the examined dicarboxylate based DILs show a lower thermal stability than the corresponding dibromides, with the onset degradation temperatures ranging between 225 and 245°C. These DILs belongs to the least stable class. Thus, comparing all the reported data, it is possible to state that the thermal stability of the investigated DILs depends primarily on the anion nature, bromides are significantly more stable than dicarboxylates, and a small effect can be also observed inside this latter class (Succinate ≈Glutarate>Malonate), whereas substituents on cation have a lower significant influence on the degradation temperature. It is also noteworthy that dicationic-dicarboxylated salts show a slightly higher thermal stability than the corresponding monocation dicarboxylated ILs (**27**-**29**). Finally, the dynamic TGA curves of the investigated dicarboxylate based DILs (Figure 1 and Supplementary Information) evidence that the thermal decomposition of these salts generally takes place in multiple steps, however, further studies are required to properly understand the contribution of the different dicarboxylate frame to the decomposition events of the IL.

**Figure 6:**
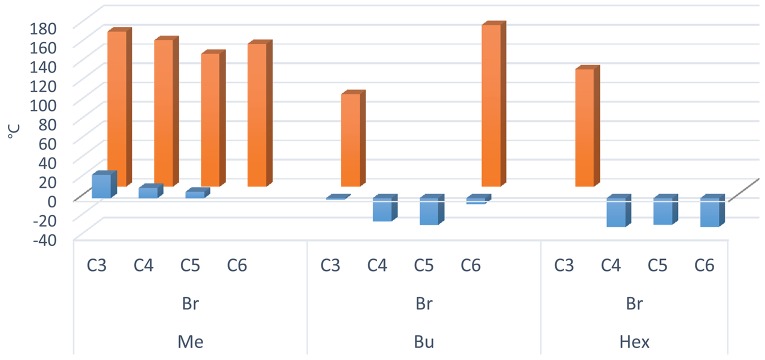
T_m_ (orange) and T_g_ (blue) values plotted against the linker alkyl chain length, grouped on the basis of the imidazole substituent (methyl, butyl or hexyl) for bromide DILs.

While TGA analysis does not show any prominent discernible trend throughout the DILs, DSC curves allowed for their division into distinct groups. To favor comparison, also in this case the T_*g*_ and T_*m*_ have been plotted against the linker alkyl chain length (Figure 6).

It should be noted that compounds with an internal C3 alkyl linker and/or 1-methylimidazolium cation with dicarboxylate or Br anions are generally white solids at room temperature. For these DILs, all the observed transitions are reversible, unless the salt is heated to a temperature which determines thermal decomposition. The other dibromide DILs with n-butyl and n-hexyl lateral alkyl chains and C4, C5, and C6 alkyl linker are generally obtained as very viscous liquids at room temperature, with the exception of **8** [C_6_(BIM)_2_/2Br] which is a white solid. The phase transitions (DSC) of the synthesized bromide DILs are summarized in Table 2.

**Table 2:** Crystallization (T_*c*_), glass transition (T_*g*_), cold crystallization (T_*cc*_), solid-solid transition (T_*ss*_), and melting (T_*m*_) temperatures for the synthesized bromide DILs.

	**2nd Cycle**									
		**Cooling**			**Heating**					
**ILs**	**T*_***c***_* (**°**C)**	**T*_***ss***_* (**°**C)**	**T*_***g***_* (**°**C)**	**T_**g**_ (**°**C)**	**T*_***cc***_* (**°**C)**	**T_**ss**_ (**°**C)**	**T_**m**_ (**°**C)**	**T_**m**_ Lit. (**°**C)**	**T_**g**_ Lit. (**°**C)**	
**1**	C_3_(MIM)_2_/2Br			20.25	24.4	66.7	97.4(exo)	161.0; 164.5^a^		
**2**	C_4_(MIM)_2_/2Br				10.6	82.5	127.5 (endo)	152.5; 88.69^b^	115.45^c^	
**3**	C_5_(MIM)_2_/2Br				6.71	90.4		137.8		
**4**	C_6_(MIM)_2_/2Br	123.9							148.4; 158.1^a^	
**5**	C_3_(BIM)_2_/2Br					−1.40	47.2		96.2	
**6**	C_4_(BIM)_2_/2Br					−24.2				
**7**	C_5_(BIM)_2_/2Br			−13.9	−27.7, −11.1					
**8**	C_6_(BIM)_2_/2Br	79.0			−6.0	40.5		167.9; 175.0^a^	151^d^	
**9**	C_3_(HIM)_2_/2Br	116.0	24.1				23.3	122.6; 26.5^a^	104^e^	−38^e^
**10**	C_4_(HIM)_2_/2Br					−30.1				
**11**	C_5_(HIM)_2_/2Br					−27.6			41.0^b^	
**12**	C_6_(HIM)_2_/2Br				−29.8					

a *Data from 1st heating-cooling cycle*.

b *Melting of dicationic ionic liquids hydrate form*.

c *Zhang et al. (2018)*.

d *Yang et al. (2014) (supplementary)*.

e *Lee et al. (2011)*.

In agreement with the general trends reported by Gómez et al. (2015), three thermal behaviors have been observed for the investigated DILs: (i) DILs showing only glass transition temperatures (type I); (ii) DILs showing freezing transitions (giving crystals) upon cooling and a melting transition upon heating (type II); (iii) DILs that don't show a crystallization in the cooling run but exhibit a cold crystallization in the heating run (type III).

**Figure 7:**
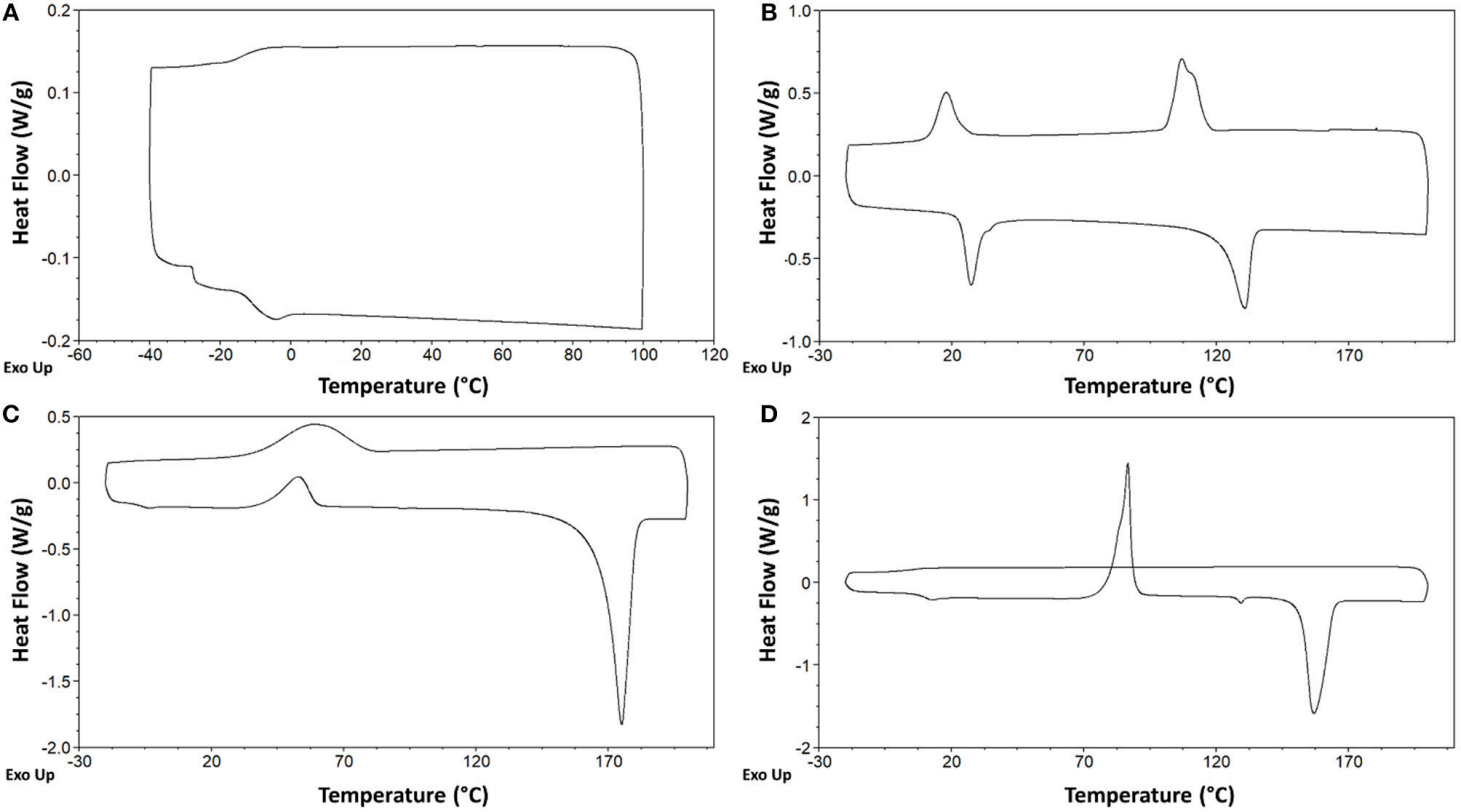
Differential scanning calorimetry (DSC) of compounds **7** [C_5_(BIM)_2_/2Br] **(A)**, **9** [C_3_(HIM)_2_/2Br] **(B)**, **8** [C_6_(BIM)_2_/2Br] **(C)**, and **2** [C_4_(MIM)_2_/2Br] **(D)**.

In particular, the five DILs which are liquid at room temperature, exhibit only glass transition(s) at low temperature without any phase transition, thus belonging to the type I. DIL **7** [C_5_(BIM)_2_/2Br] presents two different glass transitions, respectively at −27.7°C, −11.1°C in the heating run, and one at −13.9°C in the cooling run (Figure 7A). The other four salts, **6** [C_4_(BIM)_2_/2Br], **10** [C_4_(HIM)_2_/2Br], **11** [C_5_(HIM)_2_/2Br], and **12** [C_6_(HIM)_2_/2Br], show a single glass transition at −24.2°C, −30.1°C, −27.6°C, and −29.8°C, respectively. On the other hand, **4** [C_6_(MIM)_2_/2Br] and **9** [C_3_(HIM)_2_/2Br] behave as low melting salts; they crystallize in the cooling run giving a sharp crystallization peak and a comparable melting peak in the heating run (type II). For **4** [C_6_(MIM)_2_/2Br] the single crystallization and melting events have been observed at 123.9 and 148.4°C, respectively. It is noteworthy that this latter DIL displays a higher melting point during the first heating-cooling cycle (158.1°C), a behavior which is generally attributed to the thermal history of the sample. DIL **9** [C_3_(HIM)_2_/2Br] instead presents two different exothermic transitions in the cooling run (116.0°C and 24.1°C) followed by two endothermic transitions in the heating run (23.3°C and 122.6°C), Figure 7B. Since this salt presents a melting point of 123°C, when determined using a Kofler hot bench apparatus, the endothermic transition at the lower temperature can be attributed to a solid-solid transition. Finally, DILs **1** [C_3_(MIM)_2_/2Br], **2** [C_4_(MIM)_2_/2Br], **3** [C_5_(MIM)_2_/2Br], **5** [C_3_(BIM)_2_/2Br], and **8** [C_6_(BIM)_2_/2Br] exhibit an exothermic cold crystallization peak followed by a melting transition during the heating run (type III), Figure 7D. More in detail, **1** and **2** present also an exothermic and an endothermic solid-solid transition at 97.4°C and 127.5°C, respectively, whereas **8** is characterized by an even more peculiar behavior, Figure 7C. This salt shows indeed a partial crystallization at 79°C in the cooling run, followed by a small glass transition at −6.0°C, probably due to the residual amorphous part which undergoes to a cold crystallization in the heating run.

Finally, DILs based on dicarboxylated anions display simpler thermal behaviors as no transitions were observed either in the heating or in the cooling run in any of the three heating-cooling cycles. The sole transitions observed at higher temperatures can be attributed to degradation phenomena on the basis of the TGA analyses (Figure S42–S53 see Supporting Materials).

### DESs Preparation and Analyses

Following the generally reported protocol (Florindo et al., 2014), DESs were prepared by gently heating the selected components at 80°C, under stirring. Furthermore, since water can act as HBD and contribute to the formation of DES but, contemporaneously, it is able to modify all the physico-chemical properties of the resulting mixtures, all the components were accurately dried before use, and the contact with humidity was avoided during synthesis and storage. Liquid systems able to maintain this condition also during long term storage in the absence of humidity were obtained by mixing salts **4** and **20** with glycerol or diethylene glycol at the molar ratios reported in Table 3.

**Table 3:** Glass transition (T_*g*_) temperatures for the synthesized DESs.

		**T*_***gheating***_* (**°**C)**	**T*_***g***__***cooling***_*(**°**C)**
**1**	C_6_(MIM)_2_/2Br:Glycerol 1:3	−79.7	
**2**	C_6_(MIM)_2_/2Br:DiEG 1:6	−86.6	
**3**	C_6_(MIM)_2_/Succ:Glycerol 1:2	−51.1	−54.8
**4**	C_6_(MIM)_2_/Succ:DiEG 1:2	−54.1	−50.0

After further accurate drying, the four DESs studied in this work were analyzed by NMR as neat liquids to confirm that no reaction occurred under the employed conditions.

Interestingly, the ^1^H-NMR spectra registered on the neat sample (coaxial tube) at 23°C in the case of C_6_(MIM)_2_/2Br:Glycerol 1:3 and C_6_(MIM)_2_/2Br:DiEG 1:6 and at 63°C in the case of C_6_(MIM)_2_/Succ:Glycerol 1:2 and C_6_(MIM)_2_/Succ:DiEG 1:2, due to the high viscosity of these latter DESs, show significative shifts of the signals of the OH groups of both employed HBD (Figures S55–S58 see Supporting Materials). In particular, in the case of bromide based DESs an upfield shift of these signals (0.92 ppm for both OH groups of C_6_(MIM)_2_/2Br:Glycerol and 0.56 ppm for C_6_(MIM)_2_/2Br:DiEG) has been observed, suggesting a decreased hydrogen bonding on going from pure glycols to DESs. On the other hand, downfield shifts characterized the same signals in the case of succinate based DESs (1.12 ppm for C_6_(MIM)_2_/Succ:Glycerol and 1.81 ppm for C_6_(MIM)_2_/Succ:DiEG), in agreement with the higher hydrogen bond acceptor ability of the carboxylates when compared with the bromide anion. Unfortunately, the NMR spectra of pure DILs cannot be obtained, however, as upfield shift of signals of the C(2)-H imidazolium protons can be estimated considering as reference values the chemical shift measured in aprotic solvents (C_6_(MIM)_2_/Succ 9.21 ppm in CDCl_3_; C_6_(MIM)_2_/2Br 9.80 ppm in CDCl_3_ or 9.30 ppm DMSO-d_6_). This shift is attributable to decreased hydrogen bonding acceptor ability on going from bromide or succinate to the oxygen of glycols. These data strongly support the formation of DESs.

Subsequently, the thermal behavior of the obtained liquids was investigated by TGA and DSC.

Melting point and decomposition temperature are generally two important properties of DESs, particularly for the possible use of these liquids as solvents. Moreover, it was also interesting to evaluate if the thermal stability of the DILs might positively affect the decomposition temperature of the resulting DESs. The thermal properties are reported in Tables 3, 4.

**Table 4:** T_*start*_, T_*onset*_, and T_peak_ of the investigated DESs measured under a nitrogen atmosphere and with a heating rate of 10°C/min.

		**T*_***start***_* (**°**C)**	**T*_***onset***_* (**°**C)**	**T*_***peak***_* (**°**C)**
**1**	C_6_(MIM)_2_/2Br: Glycerol 1:3	198.3	202.0	252.8
			337.0	357.8
**2**	C_6_(MIM)_2_/2Br: DiEG 1:6	121.2	133.7	173.6
			325.6	347.8
**3**	C_6_(MIM)_2_/Succ: Glycerol 1:2	210.0	274.8	324.3
**4**	C_6_(MIM)_2_/Succ: DiEG 1:2	167.6	168.2	212.2
			274.1	292.2
				319.4

**Figure 8:**
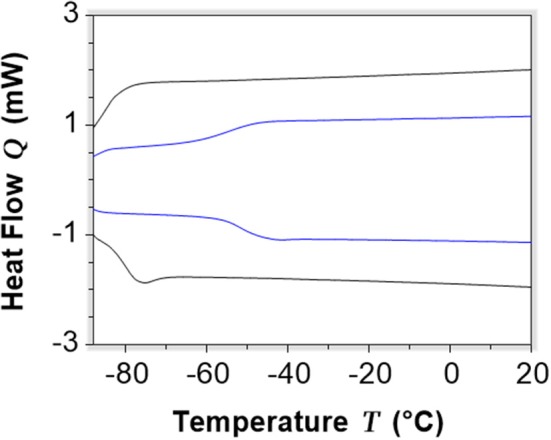
DSC curves of selected DESs [C_6_(MIM)_2_/2Br - Glycerol 1:3 in blue, C_6_(MIM)_2_/succinate - Glycerol 1:2 in black).

The investigated DESs only present a glass transition at −79.7°C, −86.6°C, −51.1°C, and −54.1°C, respectively (Table 3; Figure 8), which confirm that these systems are supramolecular complexes with a liquid state over a wide range of temperatures.

**Figure 9:**
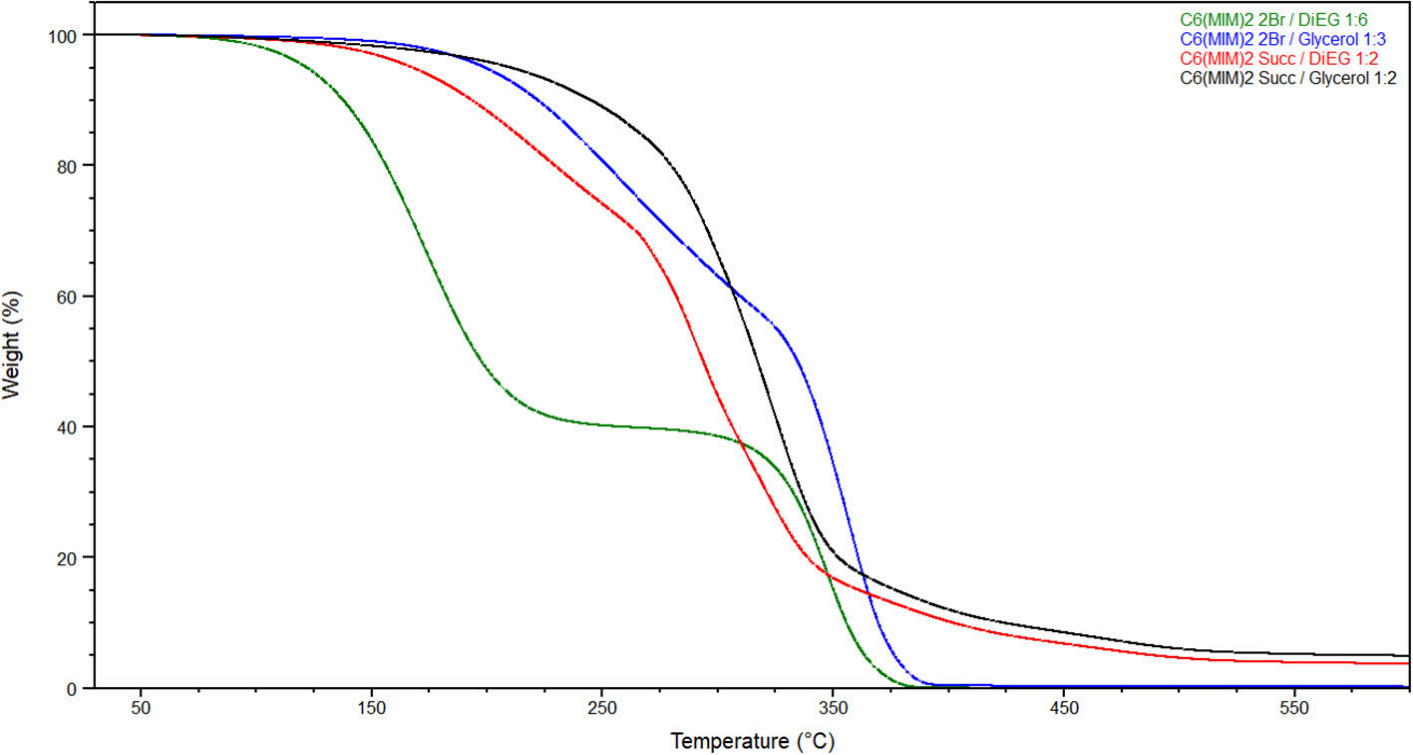
Thermal gravimetric analysis (TGA) of prepared DESs with heating rate of 10°C/min.

For all the investigated DESs, the first decomposition event is strictly related to the more volatile component (Figure 9). Indeed, the lower decomposition temperatures were observed when diethylene glycol was used as the hydrogen bond donor for both DILs (133.7°C for C_6_(MIM)_2_/2Br:DiEG 1:6 and 168.2°C for C_6_(MIM)_2_/Succ:DiEG 1:2). It is also interesting to note how, for the same HBD, bromide dicationic based DESs resulted in being less stable than the succinate based DESs, which is the opposite of what was observed for pure DILs. This is probably due to either the stronger interaction between the carboxylate anion and the HBD than between the bromide anion and the HBD or to the higher amount of HBD required by dicationic bromide to form the DES or by a combination of the two.

## Conclusions

A homogeneous class of 12 dibromide DILs has been prepared by performing the Menshutkin reaction in MIBK, a greener solvent than the frequently used toluene, between alkyl imidazoles and 1,n-dibromo alkanes. Methyl imidazolium bromide DILs, characterized by a different linker length between the cationic moieties (C3-C6), were converted into malonate, succinate, and glutarate dicarboxylate salts. The most common bromide-carboxylate anion exchange protocols were compared. In our hands, the resin exchange procedure performed the best, and allowed for the preparation of all the desired dicarboxylate salts in high yield and purity. The thermal behaviors of the dibromide and dicarboxylate DILs were then investigated by TGA and DSC. For the dibromide DILs, TGA analysis showed an effect of the lateral alkyl substituent of the imidazolium cation on the thermal stability, while the internal linker seems to play a negligible role. DSC analysis revealed a quite complex picture of the thermal behavior, which confirmed how subtle structural changes affect phase transitions of ILs. As for the dicarboxylated DILs, lower thermal stabilities were found for all the prepared salts.

Two reference compounds, solids at room temperature, were also used as hydrogen bond acceptor (HBA) components for the preparation of DESs with either glycerol or diethylene glycol. The four liquid systems obtained were analyzed by ^1^H NMR, which ascertained the formation of hydrogen bonds between the DES components, and by TGA and DSC, which showed the influence on the thermal stability of both the HBD and of the anion of the DIL. The systematic investigation of DILs thermal behavior as well as their use as potential DES components should allow for a future rational design of new liquid systems (DILs and DES) and their use in potential new applications. Studies to ascertain the (eco)toxicity of the proposed DILs are currently underway and will be presented in the due course.

## Author Contributions

LGug and AM worked on the presented work under the guide of LGua, CP, and FD. The manuscript was written by CC with the input from all authors.

### Conflict of Interest Statement

The authors declare that the research was conducted in the absence of any commercial or financial relationships that could be construed as a potential conflict of interest.
