# Supplementary material for: Systematic Synthesis and Properties Evaluation of Dicationic Ionic Liquids, and a Glance Into a Potential New Field
Source: Front Chem. 2018 Dec 12;6:612. doi: 10.3389/fchem.2018.00612 (PMC6299102; doi:10.3389/fchem.2018.00612)

***Supporting Information***

**Systematic synthesis and properties evaluation of** [**Dicationic**](http://www.frontiersin.org/Green_and_Sustainable_Chemistry/researchtopics/Ionic_Liquids_Properties_and_Applications/7972) **Ionic Liquids, and a glance into a potential new field.**

Luca Guglielmero, Andrea Mezzetta, Lorenzo Guazzelli, Christian S. Pomelli, Felicia D’Andrea, Cinzia Chiappe

*Department of Pharmacy, University of Pisa, 56126 Pisa*

***Table of contents***

Thermal gravimetric analysis (TGA) of compounds **1**-**29** page S2-S30.

Differential scanning calorimetry (DSC) of compounds **1**-**12** pages S31-S42.

Degradation of compound **13-24** pages S43-S54.

^1^H NMR spectra of DESs pages S55-S58**.**

**Figure S1**. Thermal gravimetric analysis of compound C_3_(MIM)_2_/2Br (**1**)**.**

**
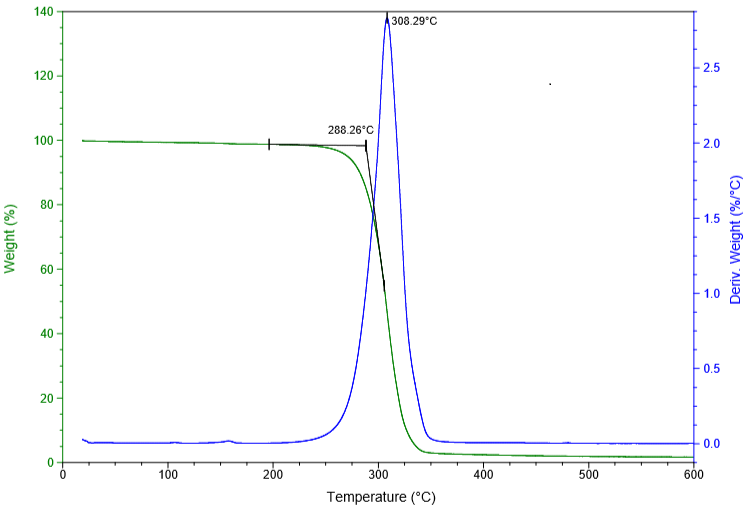
**

**Figure S2**. Thermal gravimetric analysis of compound C_4_(MIM)_2_/2Br (**2**)**.**

**
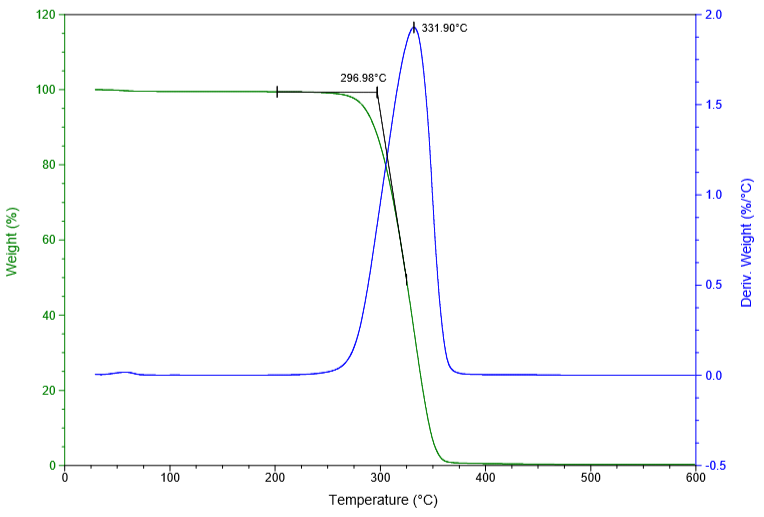
**

**Figure S3**. Thermal gravimetric analysis of compound C_5_(MIM)_2_/2Br (**3**)**.**

**
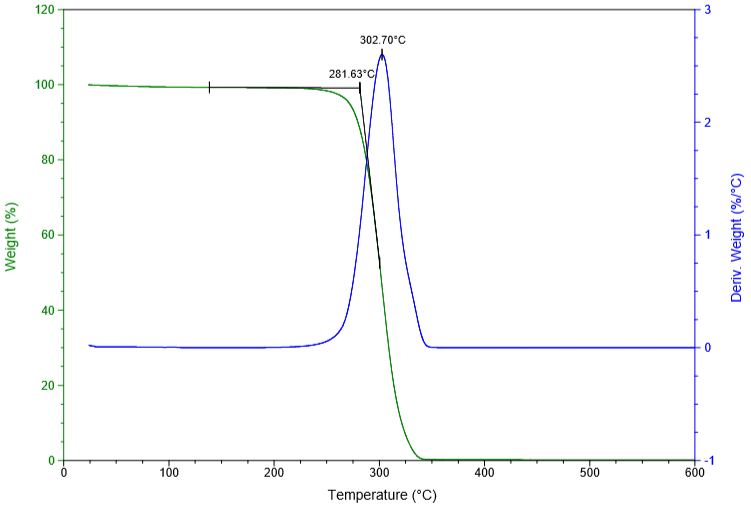
**

**Figure S4**. Thermal gravimetric analysis of compound C_6_(MIM)_2_/2Br (**4**)**.**

**
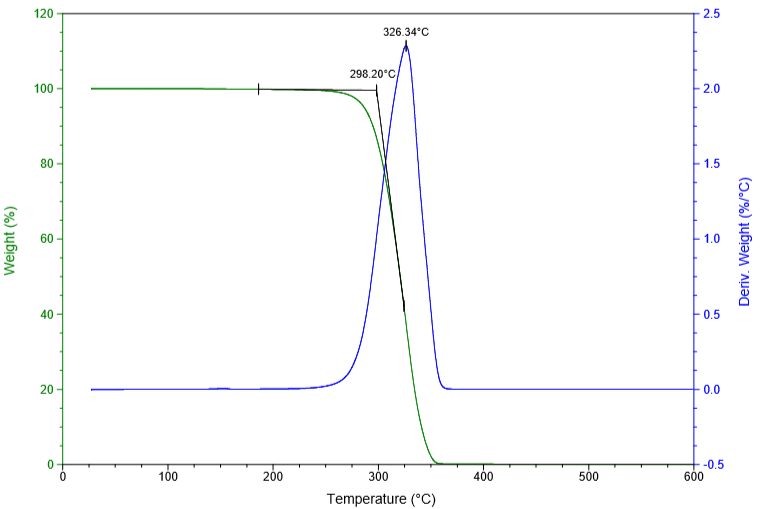
**

**Figure S5**. Thermal gravimetric analysis of compound C_3_(BIM)_2_/2Br (**5**)**.**


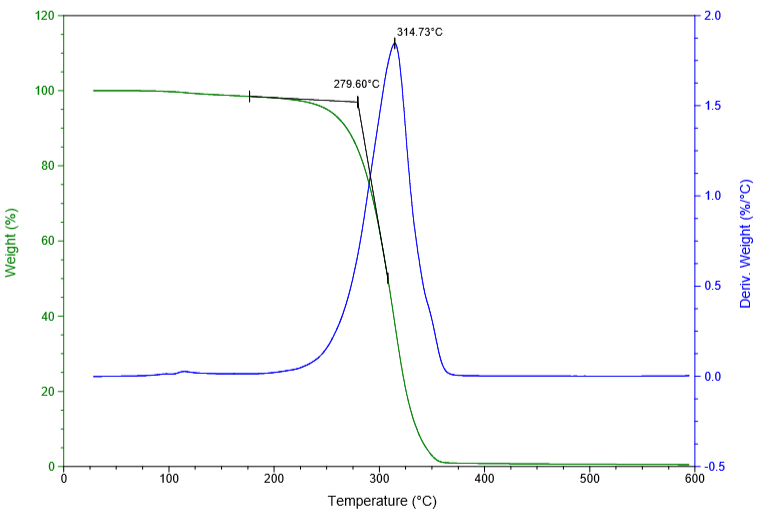


**Figure S6**. Thermal gravimetric analysis of compound C_4_(BIM)_2_/2Br (**6**)**.**

**
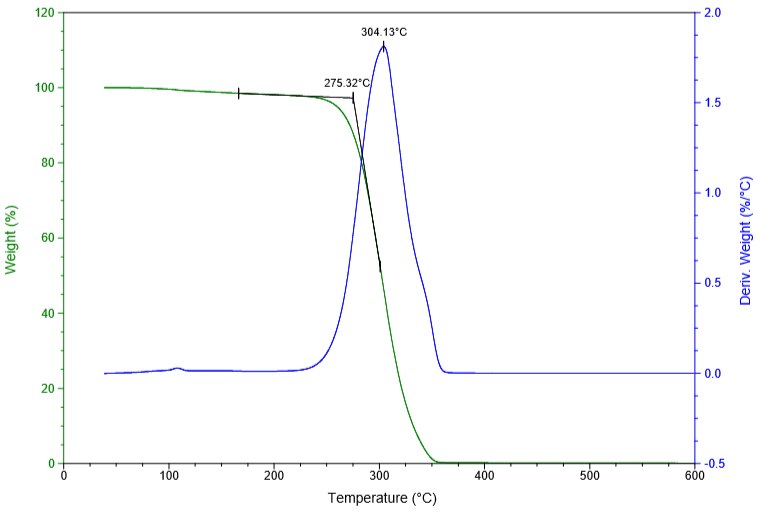
**

**Figure S7**. Thermal gravimetric analysis of compound C_5_(BIM)_2_/2Br (**7**)**.**

**
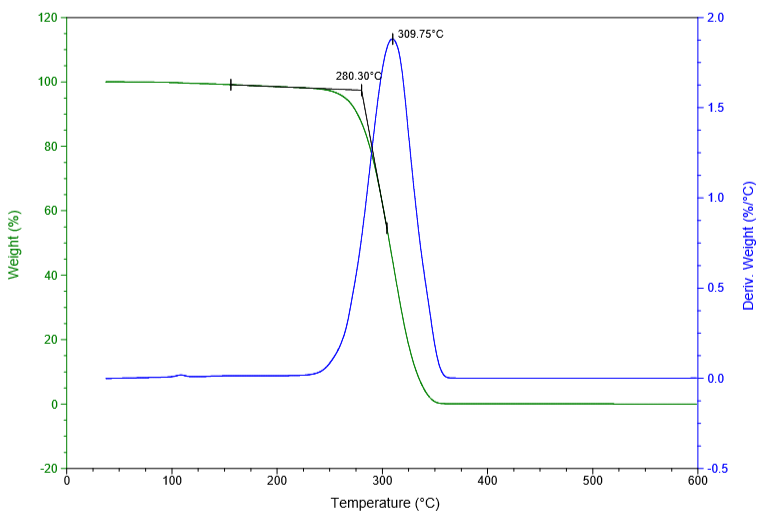
**

**Figure S8**. Thermal gravimetric analysis of compound C_6_(BIM)_2_/2Br (**8**)**.**

**
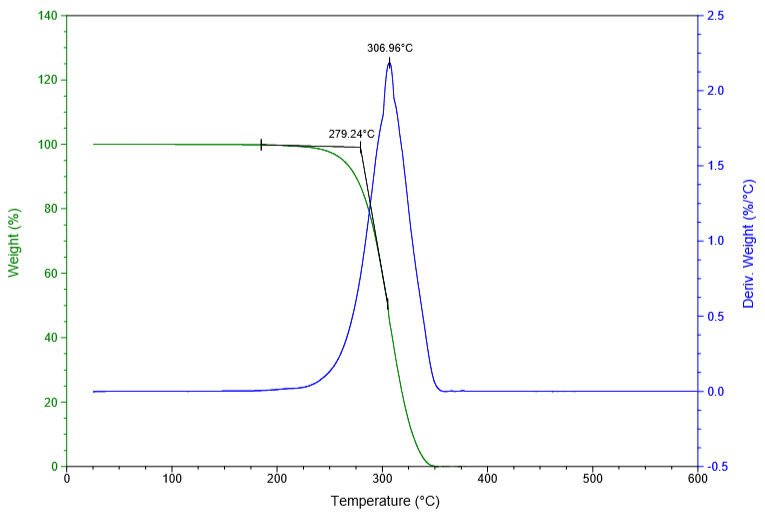
**

**Figure S9**. Thermal gravimetric analysis of compound C_3_(HIM)_2_/2Br (**9**)**.**

**
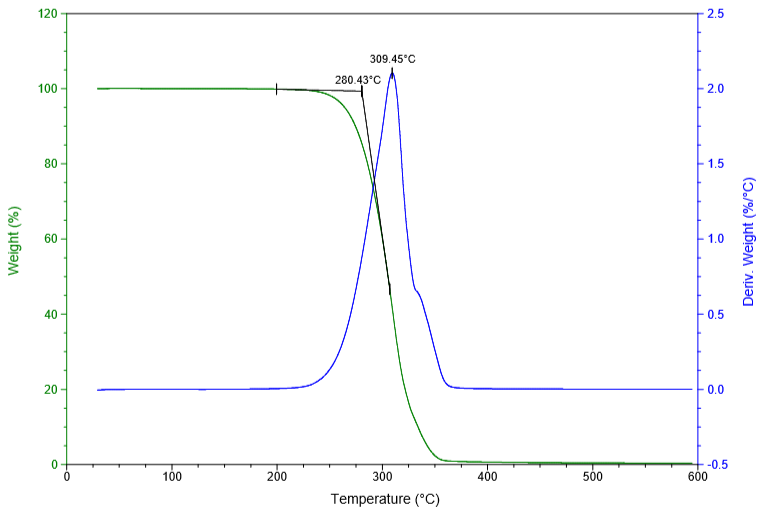
**

**Figure S10**. Thermal gravimetric analysis of compound C_4_(HIM)_2_/2Br (**10**)**.**

**
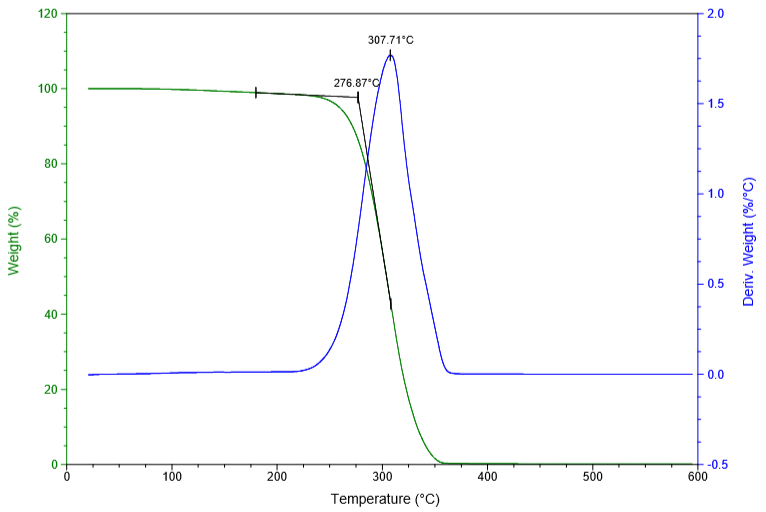
**

**Figure S11**. Thermal gravimetric analysis of compound C_5_(HIM)_2_/2Br (**11**)**.**

**
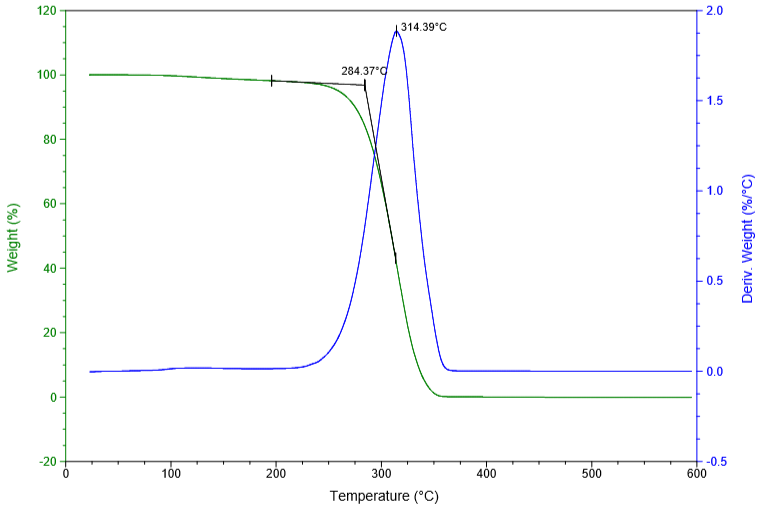
**

**Figure S12**. Thermal gravimetric analysis of compound C_6_(HIM)_2_/2Br (**12**)**.**

**
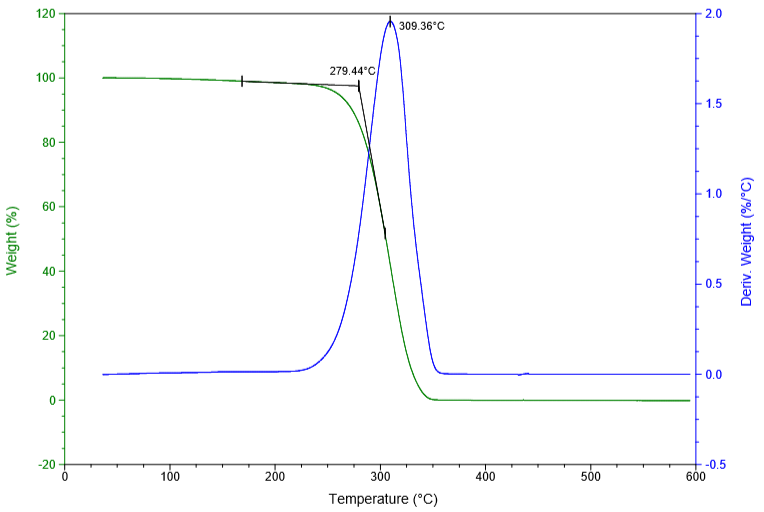
**

**Figure S13**. Thermal gravimetric analysis of compound C_3_(MIM)_2_/Mal (**13**)**.**

**
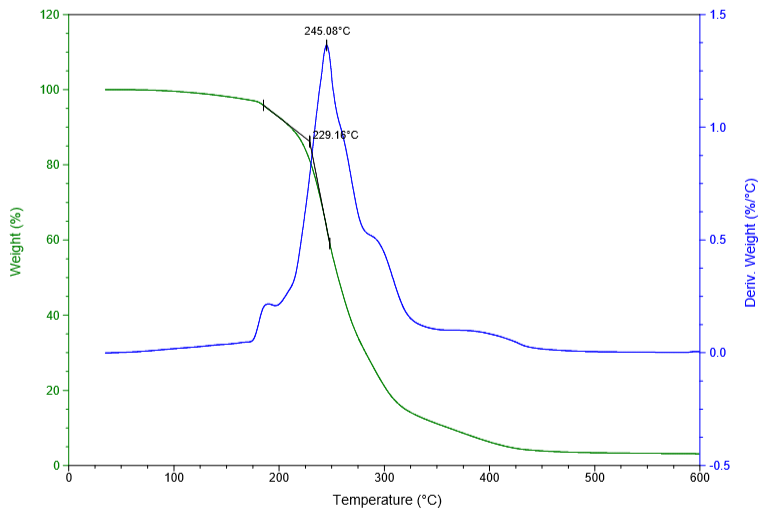
**

**Figure S14**. Thermal gravimetric analysis of compound C_4_(MIM)_2_/Mal (**14**)**.**

**
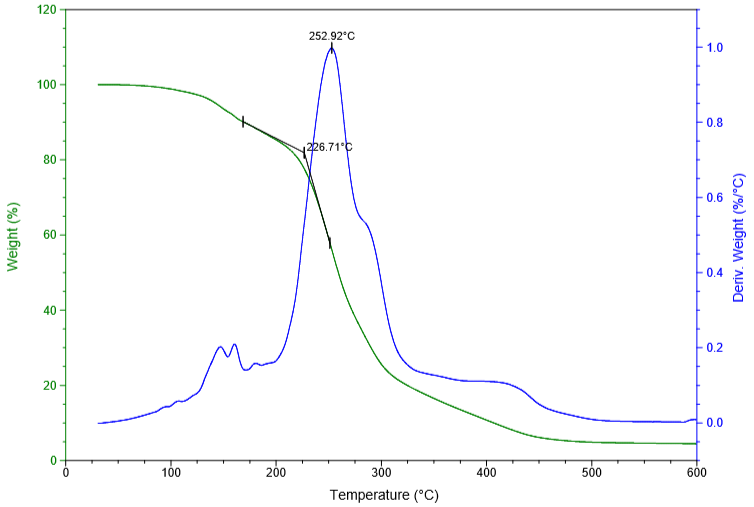
**

**Figure S15**. Thermal gravimetric analysis of compound C_5_(MIM)_2_/Mal (**15**)**.**

**
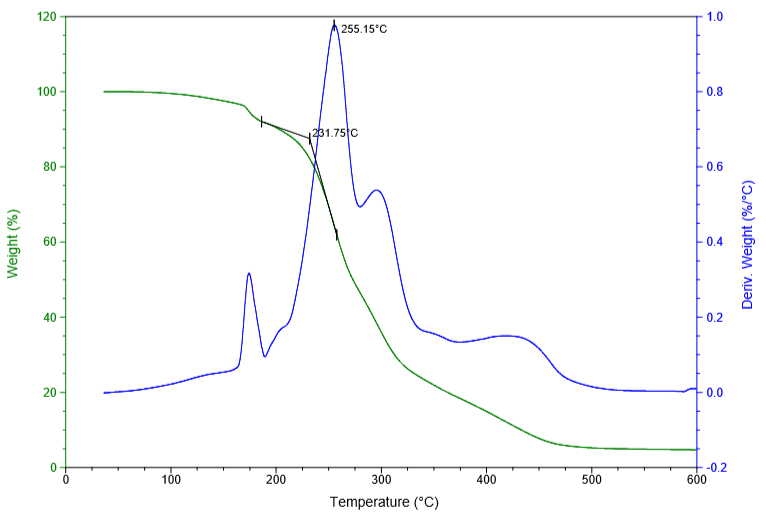
**

**Figure S16**. Thermal gravimetric analysis of compound C_6_(MIM)_2_/Mal (**16**)**.**

**
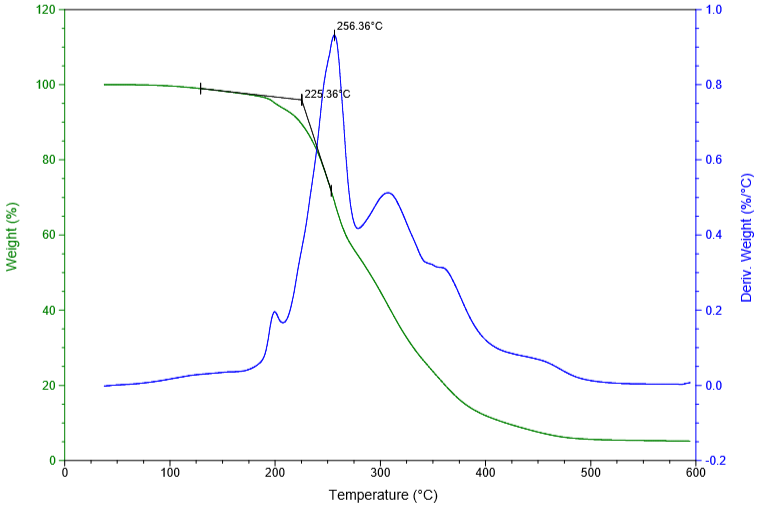
**

**Figure S17**. Thermal gravimetric analysis of compound C_3_(MIM)_2_/Succ (**17**)**.**

**
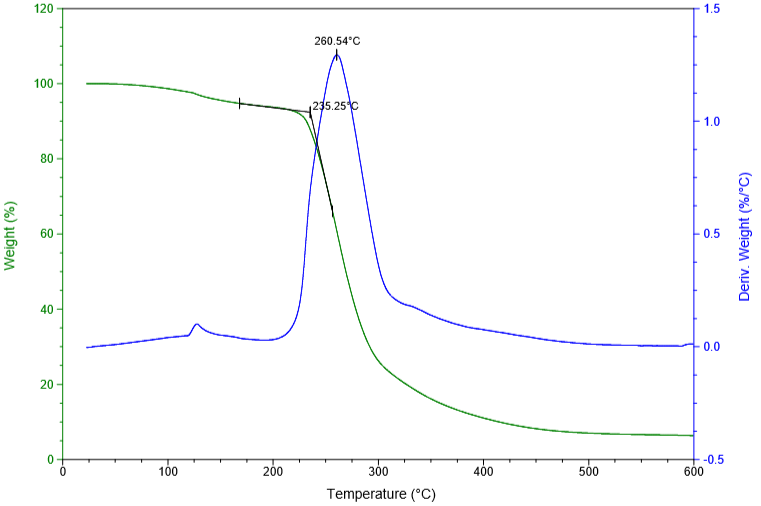
**

**Figure S18**. Thermal gravimetric analysis of compound C_4_(MIM)_2_/Succ (**18**)**.**

**
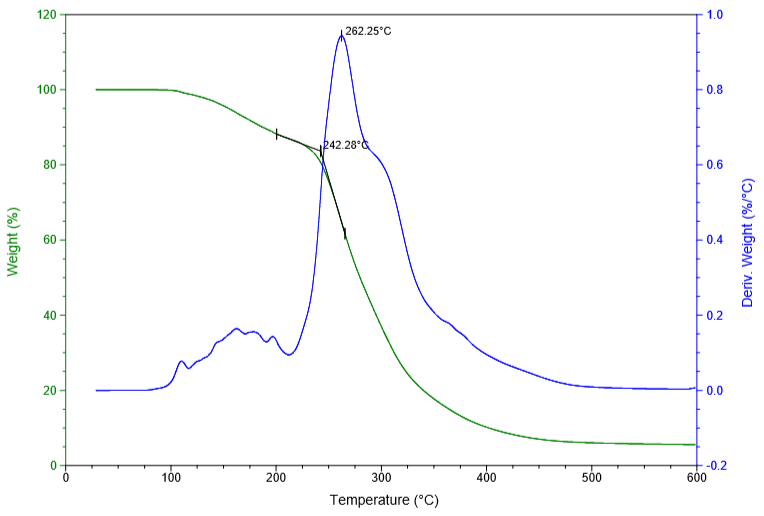
**

**Figure S19**. Thermal gravimetric analysis of compound C_5_(MIM)_2_/Succ (**19**)**.**

**
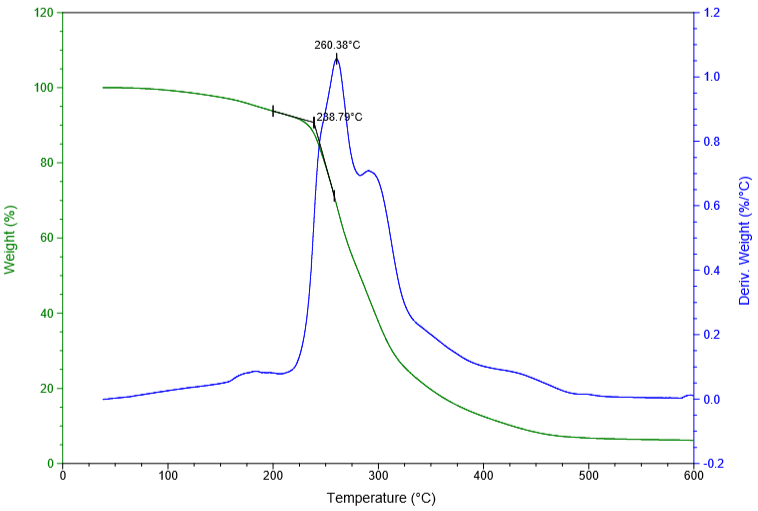
**

**Figure S20**. Thermal gravimetric analysis of compound C_6_(MIM)_2_/Succ (**20**)**.**

**
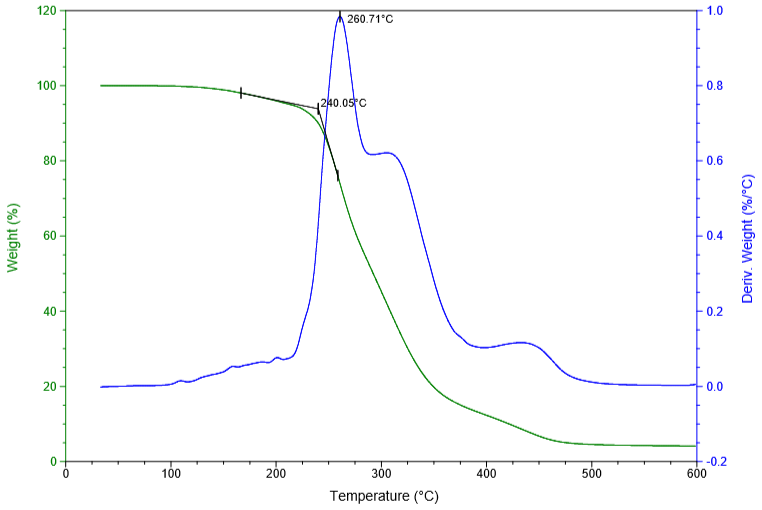
**

**Figure S21**. Thermal gravimetric analysis of compound C_3_(MIM)_2_/Glut (**21**)**.**

**
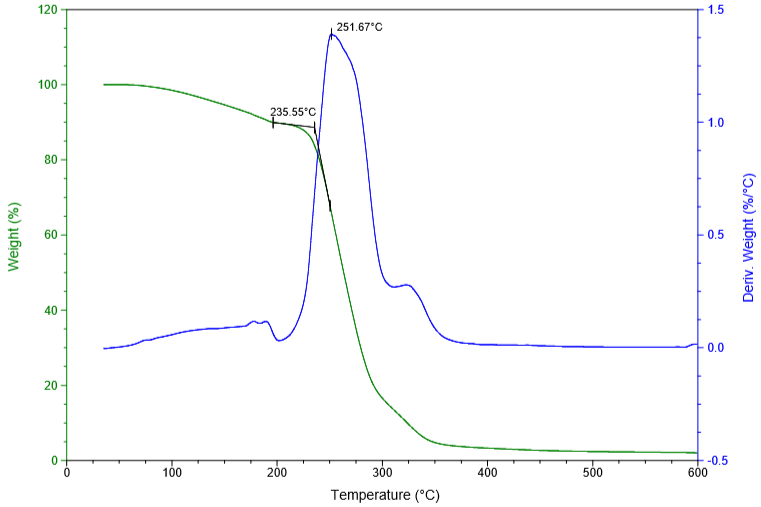
**

**Figure S22**. Thermal gravimetric analysis of compound C_4_(MIM)_2_/Glut (**22**)**.**

**
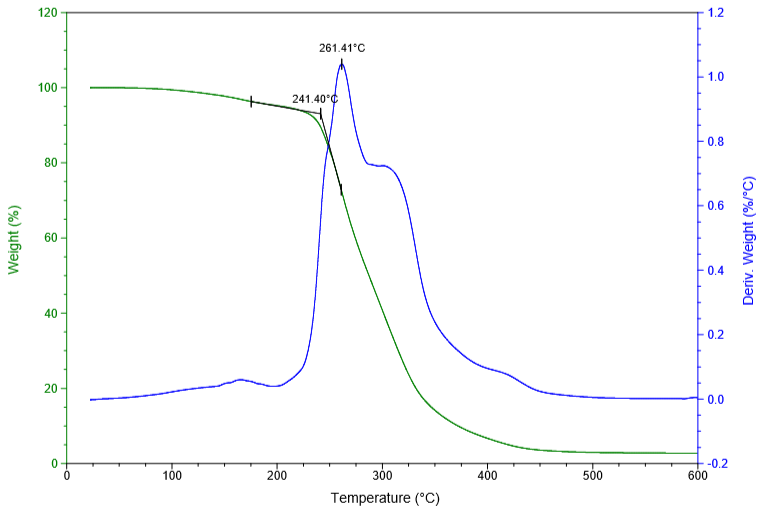
**

**Figure S23**. Thermal gravimetric analysis of compound C_5_(MIM)_2_/Glut (**23**)**.**

**
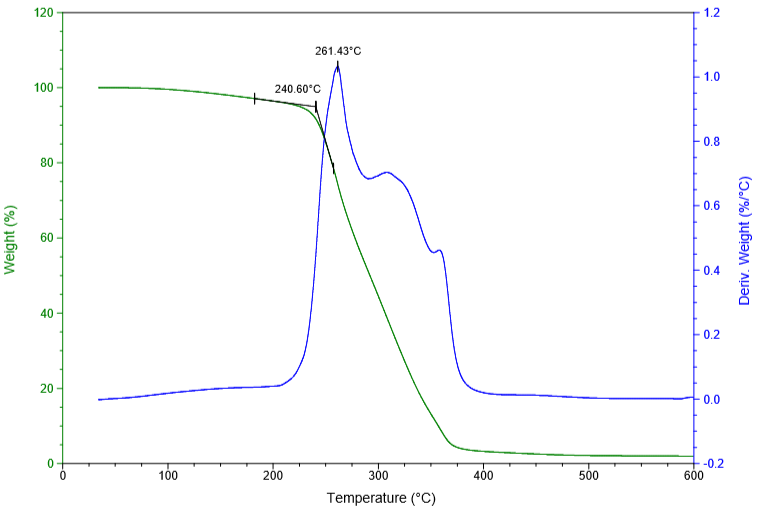
**

**Figure S24**. Thermal gravimetric analysis of compound C_6_(MIM)_2_/Glut (**24**)**.**

**
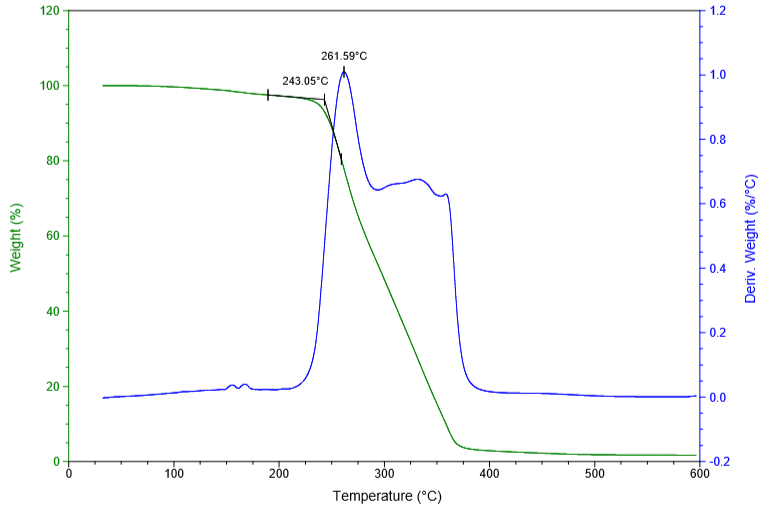
**

**Figure S25**. Thermal gravimetric analysis of compound C_4_(BIM)_2_/Succ (**25**)**.**

**
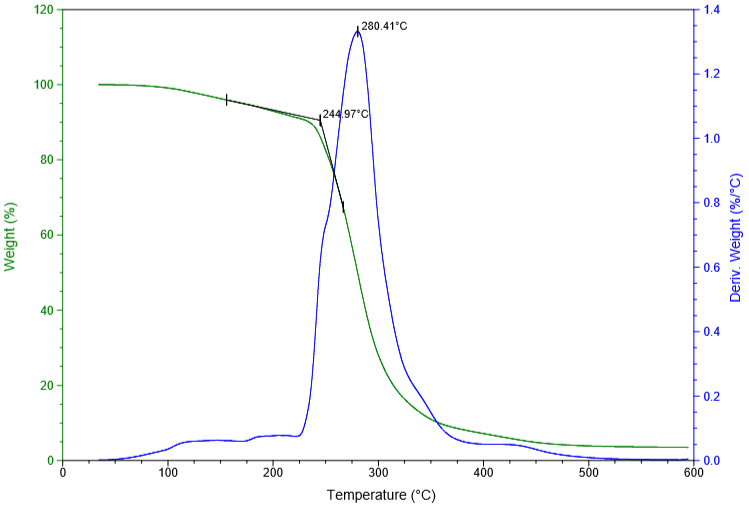
**

**Figure S26**. Thermal gravimetric analysis of compound C_4_(HIM)_2_/Succ (**26**)**.**

**
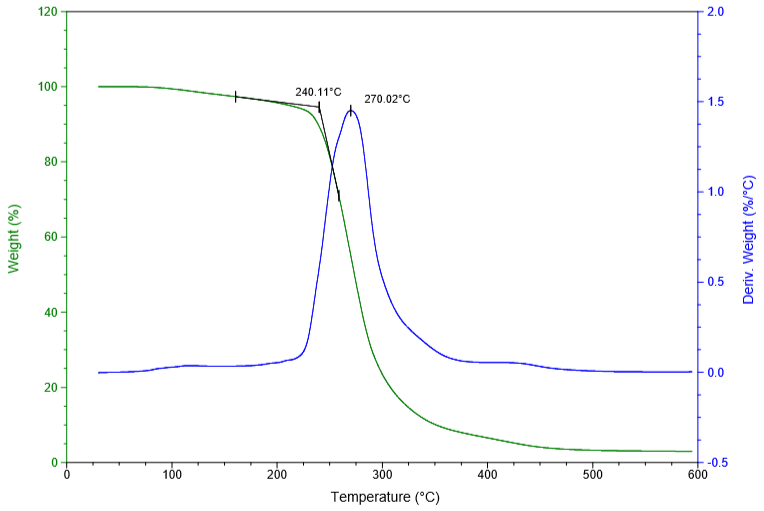
**

**Figure S27**. Thermal gravimetric analysis of compound BMIM/Mal (**27**)**.**

**
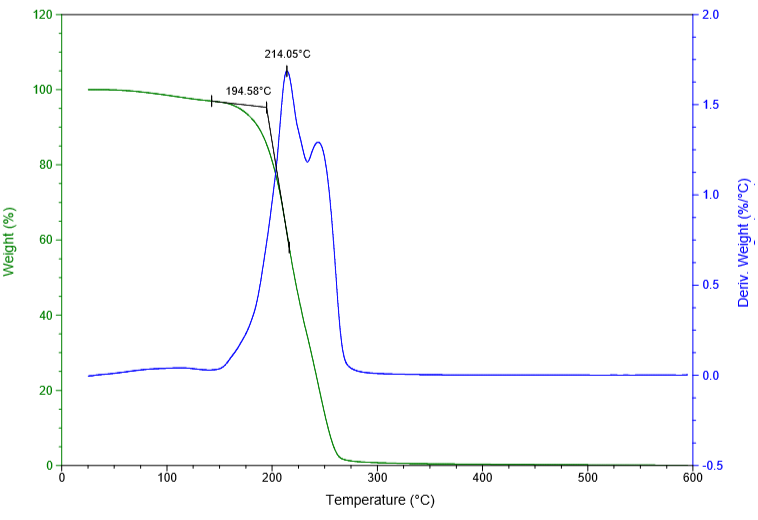
**

**Figure S28**. Thermal gravimetric analysis of compound BMIM/Succ (**28**)**.**

**
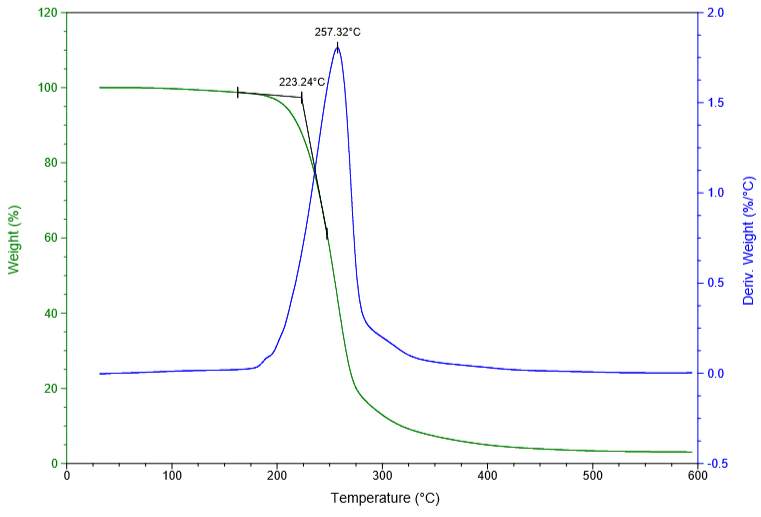
**

**Figure S29**. Thermal gravimetric analysis of compound BMIM/Glut (**29**)**.**

**
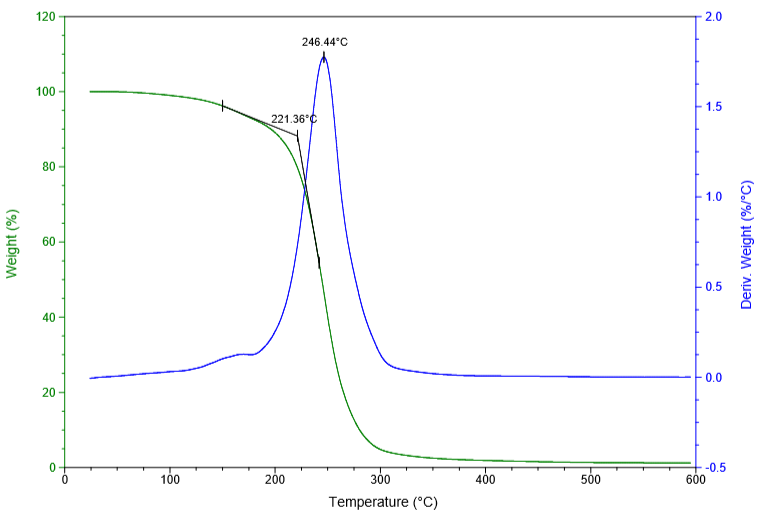
**

**Figure S30.** Differential scanning calorimetry (DSC) of compounds C_3_(MIM)_2_/2Br (**1**).

**
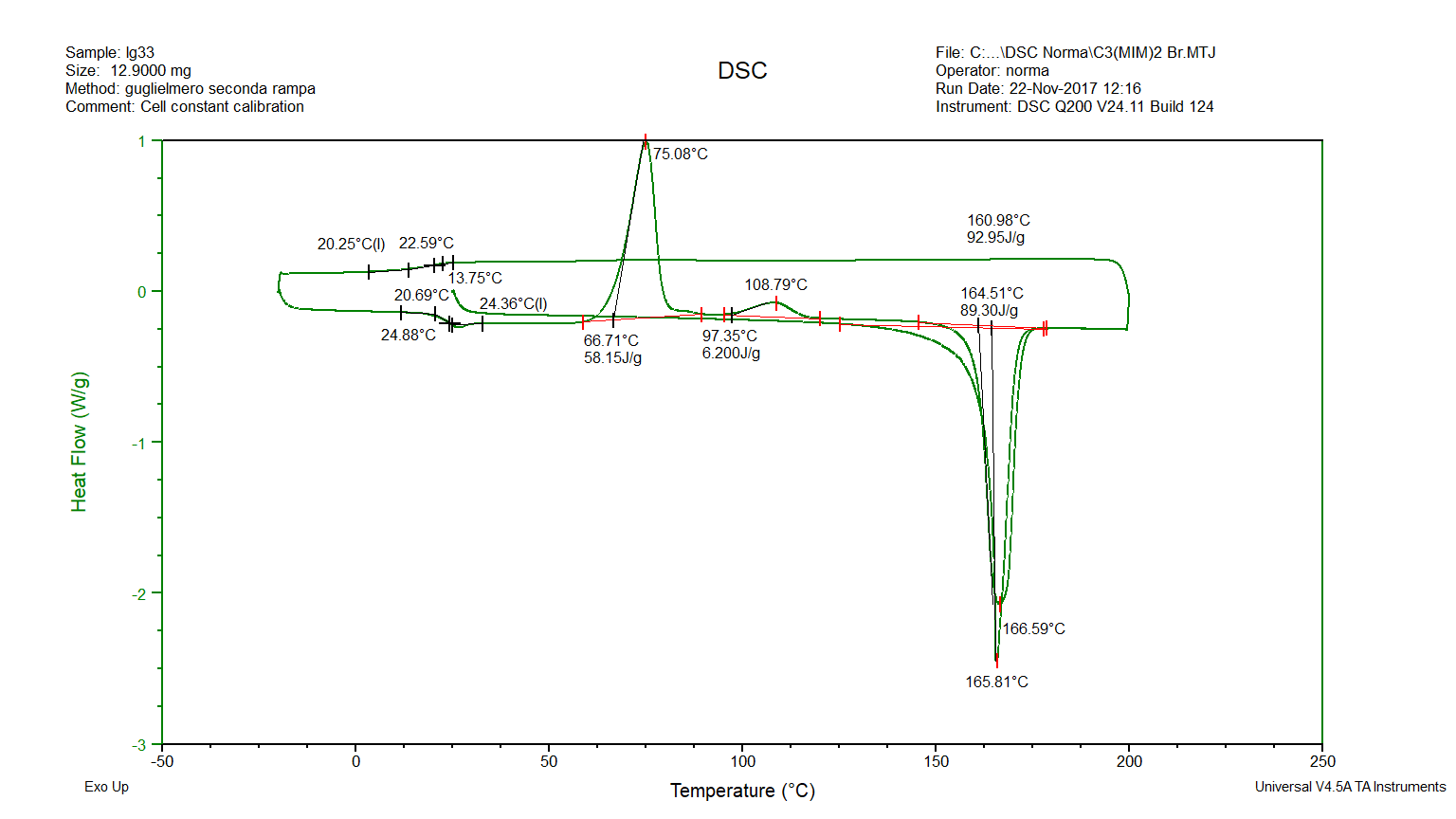
**

**Figure S31.** Differential scanning calorimetry (DSC) of compounds C_4_(MIM)_2_/2Br (**2**).

**
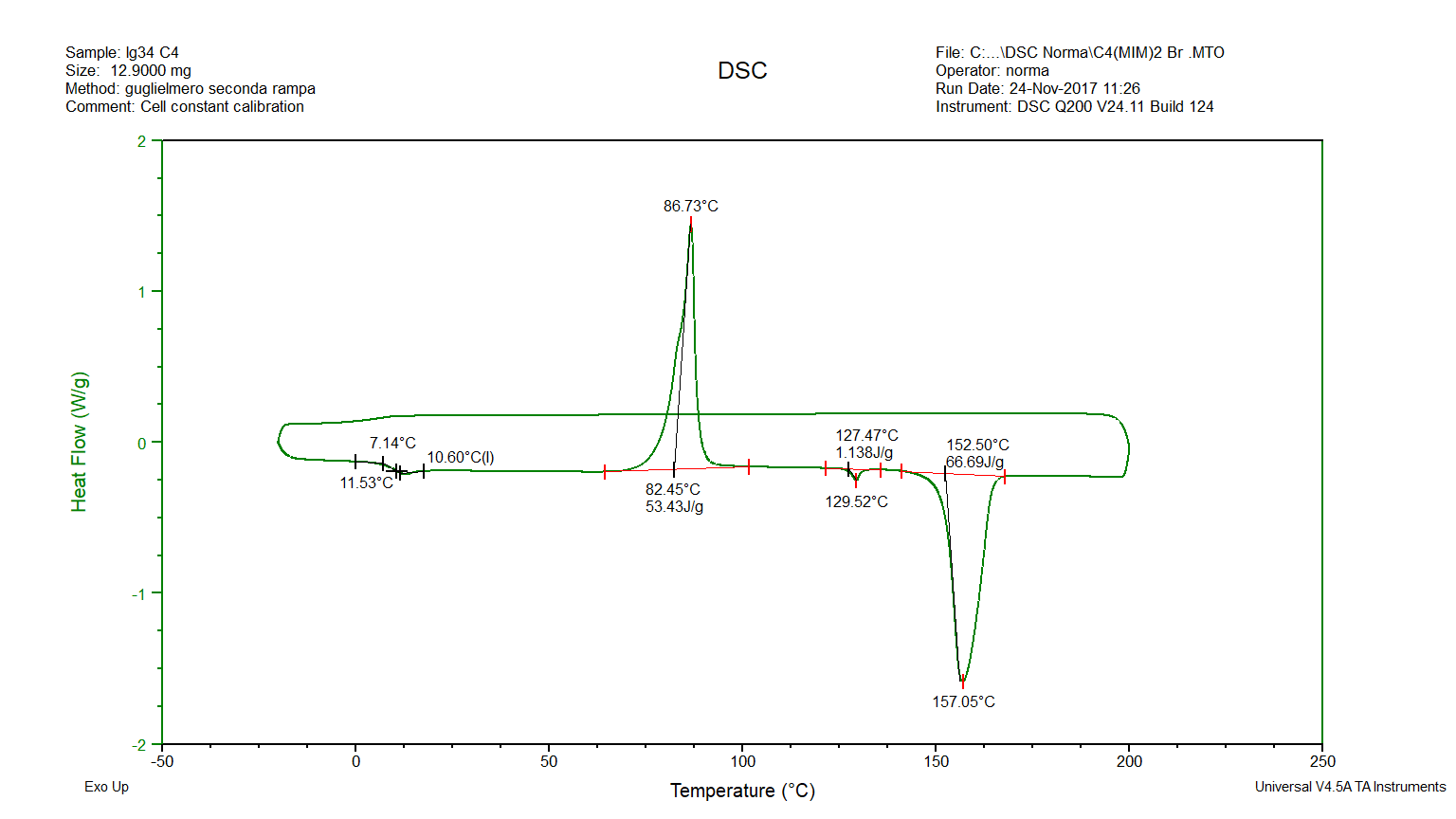
**

**Figure S32.** Differential scanning calorimetry (DSC) of compounds C_5_(MIM)_2_/2Br (**3**).


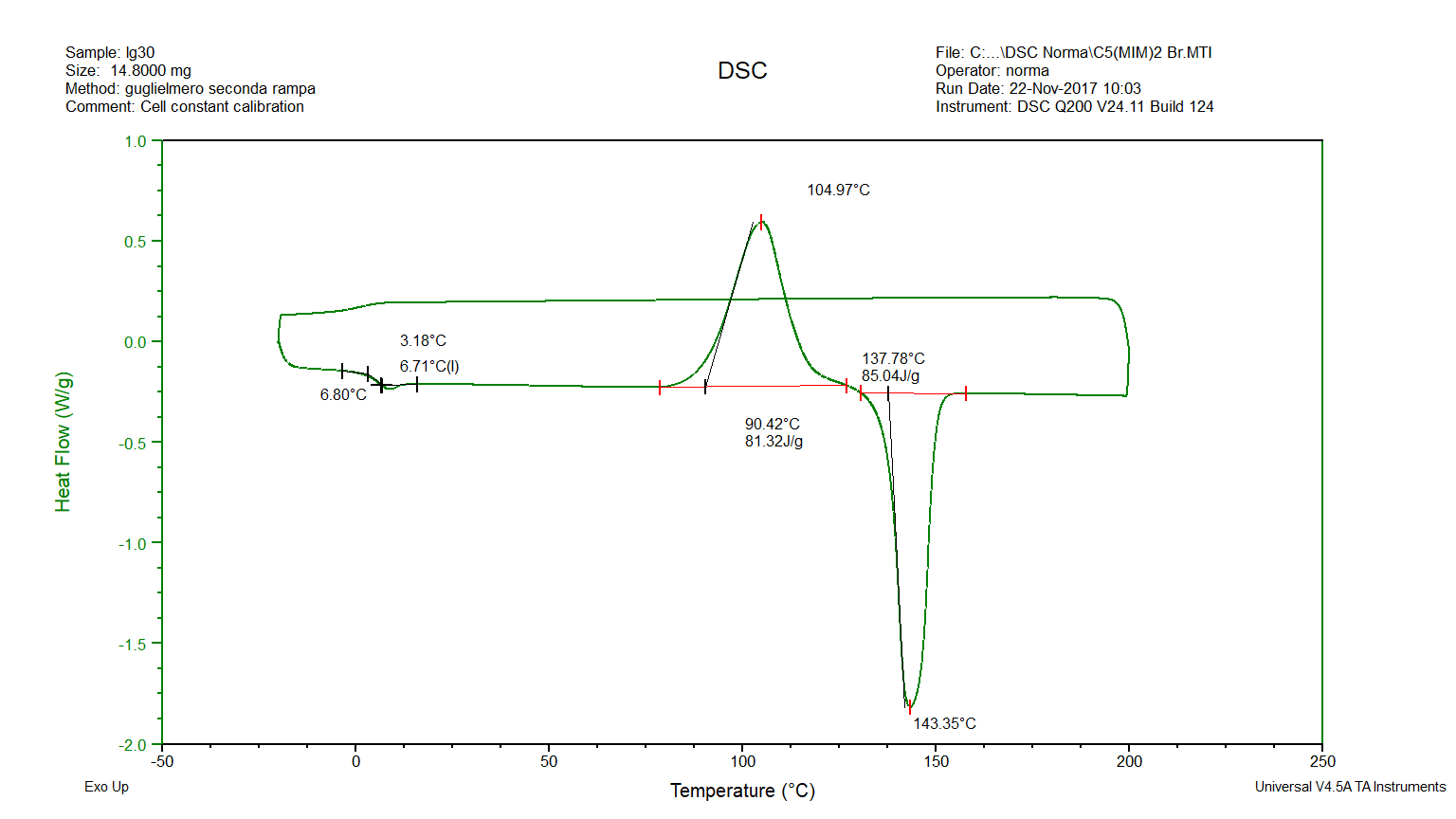


**Figure S33.** Differential scanning calorimetry (DSC) of compounds C_6_(MIM)_2_/2Br (**4**).


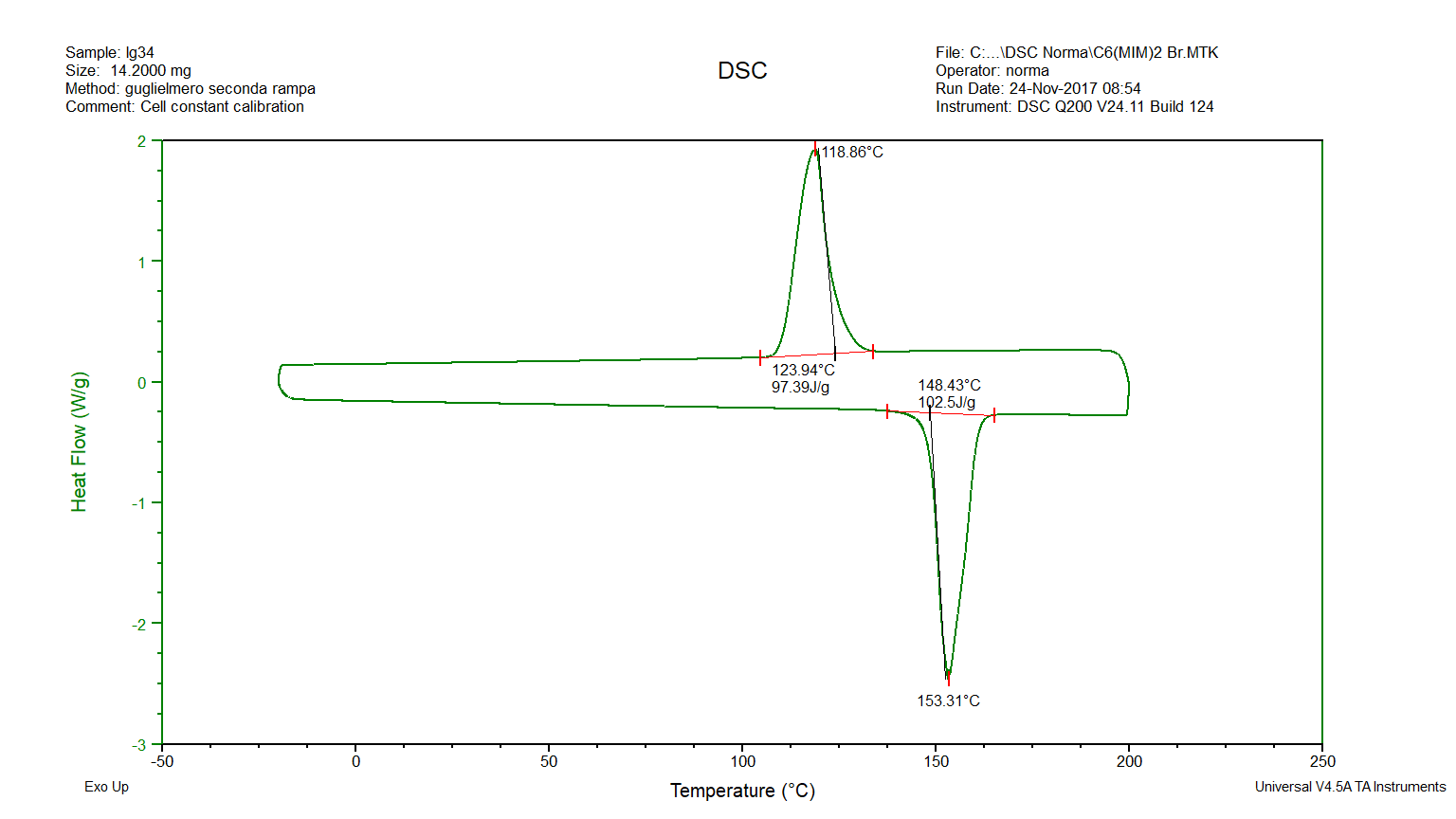


**Figure S34.** Differential scanning calorimetry (DSC) of compounds C_3_(BIM)_2_/2Br (**5**).


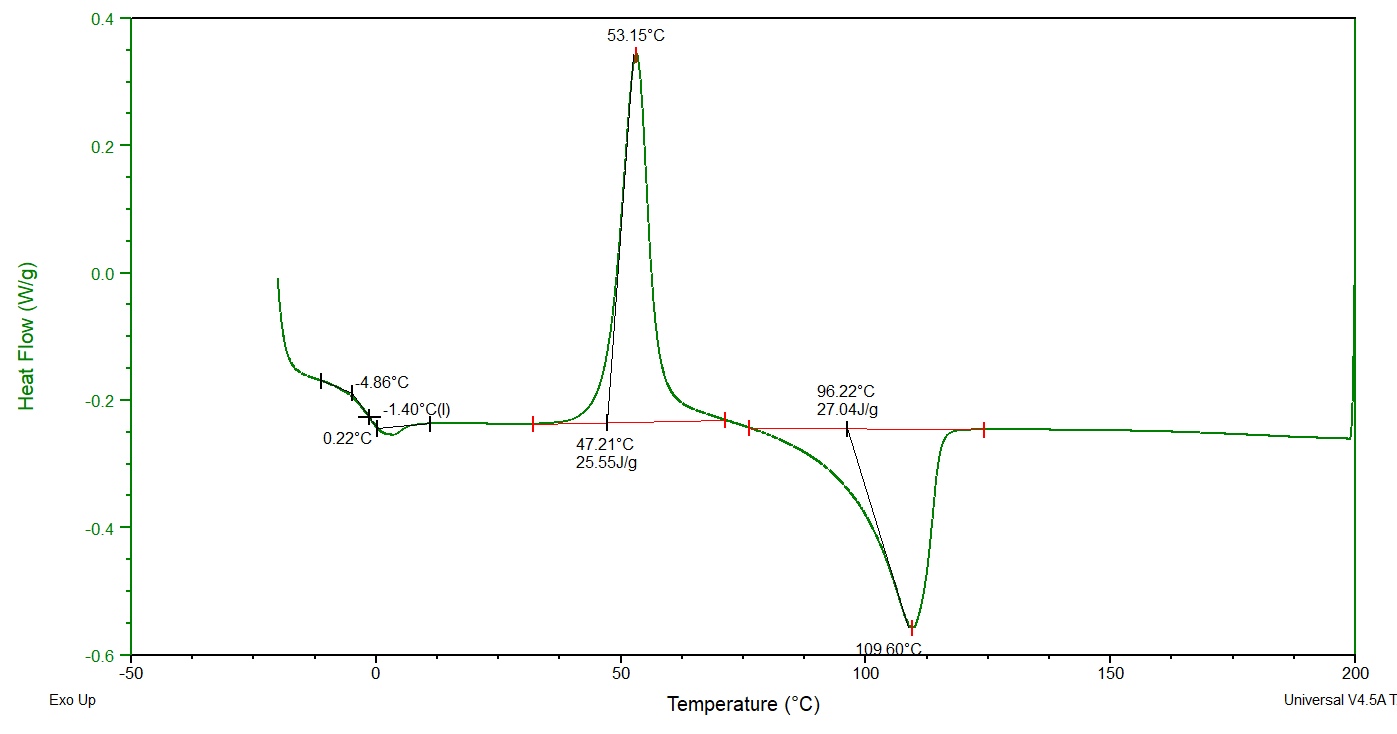


**Figure S35.** Differential scanning calorimetry (DSC) of compounds C_4_(BIM)_2_/2Br (**6**).


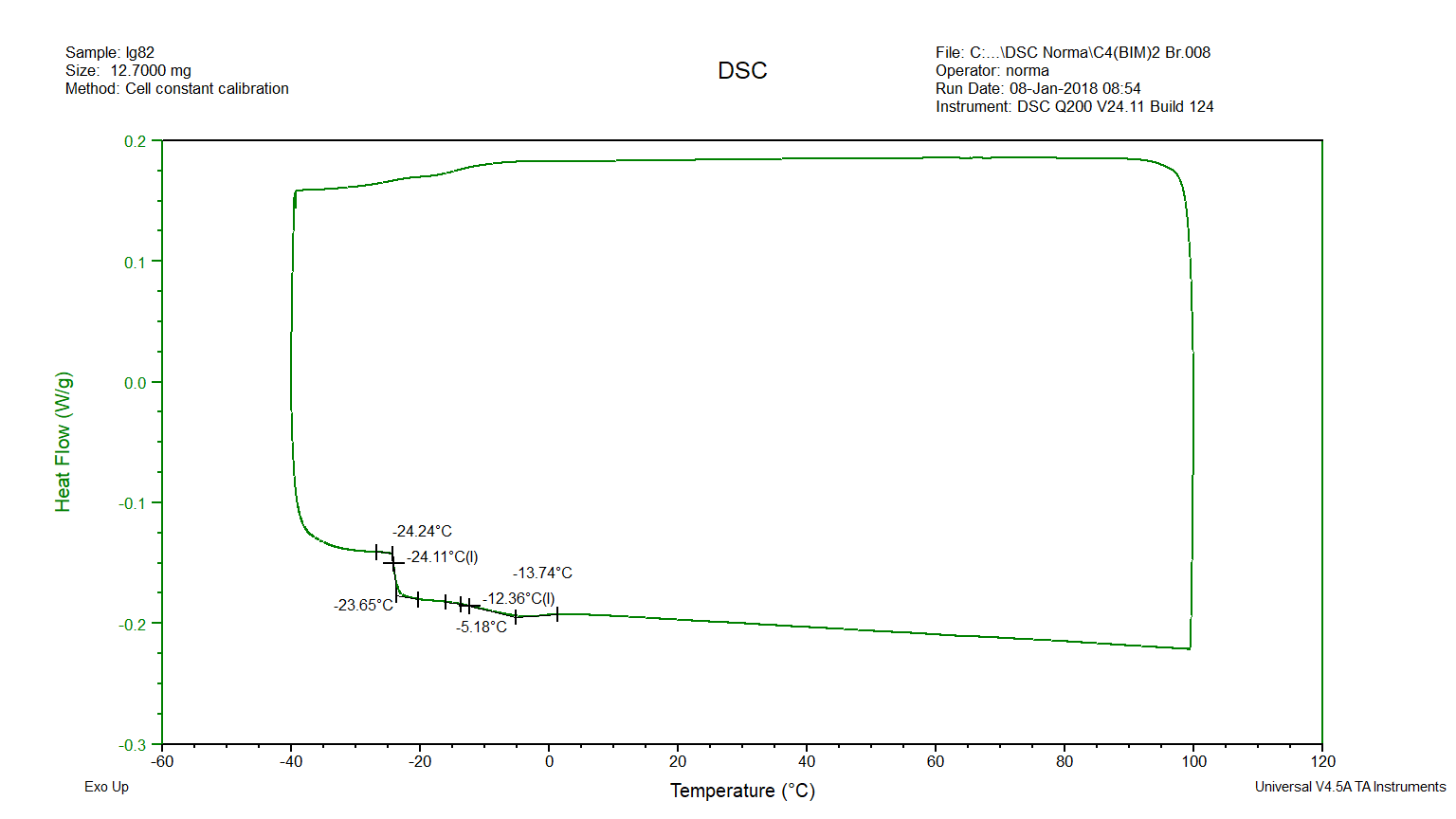


**Figure S36.** Differential scanning calorimetry (DSC) of compounds C_5_(BIM)_2_/2Br (**7**).


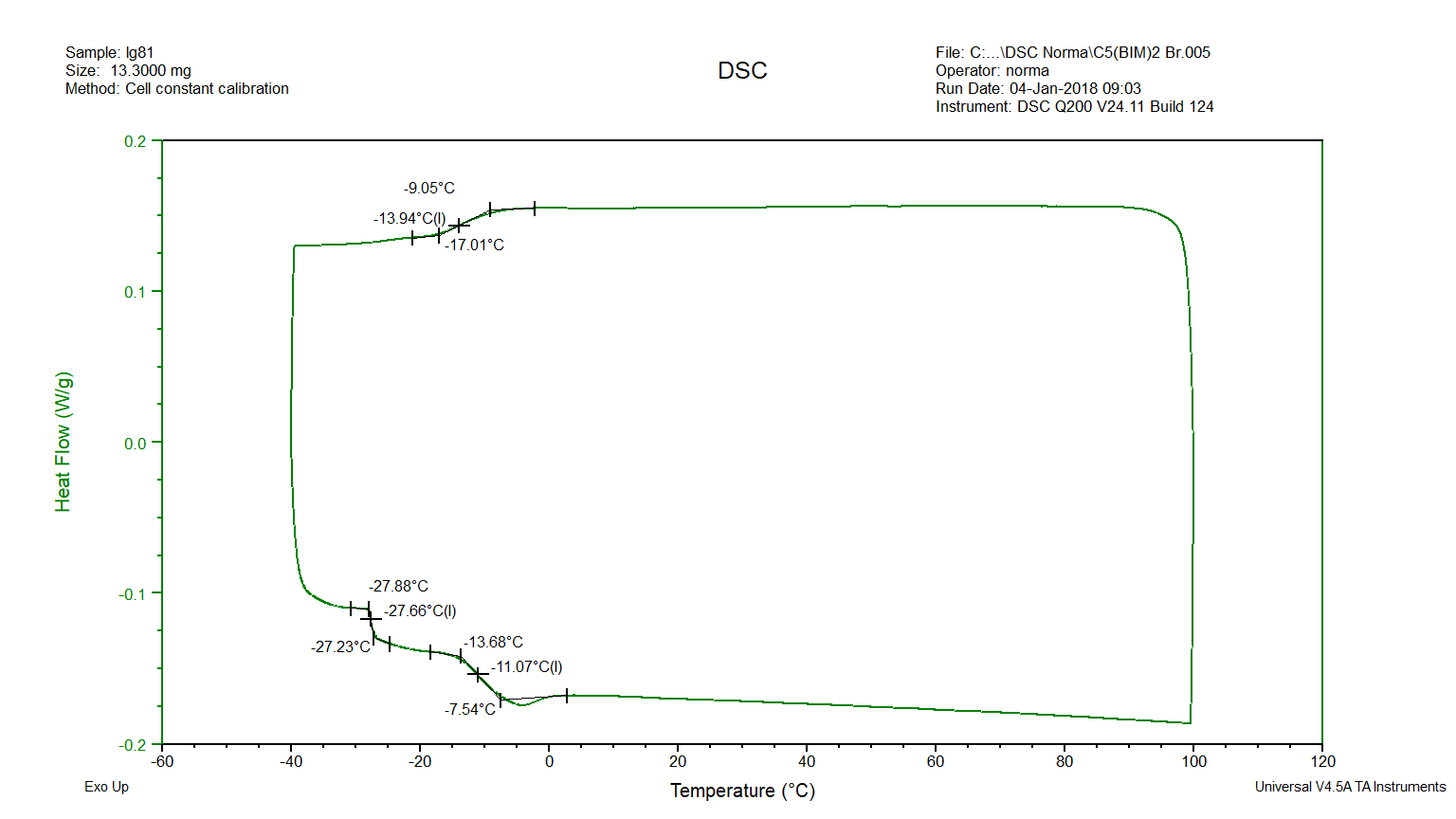


**Figure S37.** Differential scanning calorimetry (DSC) of compounds C_6_(BIM)_2_/2Br (**8**).


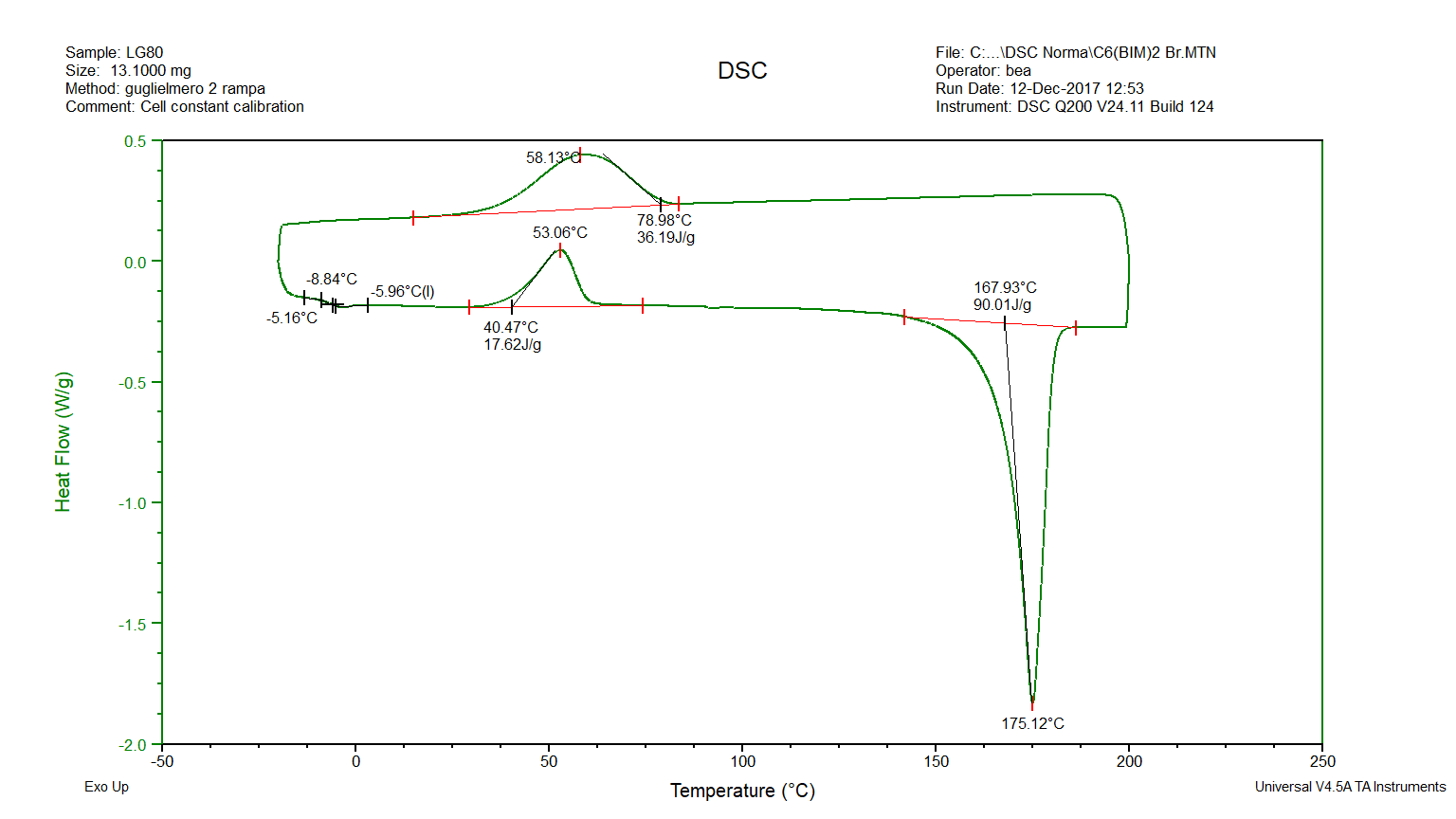


**Figure S38.** Differential scanning calorimetry (DSC) of compounds C_3_(HIM)_2_/2Br (**9**).


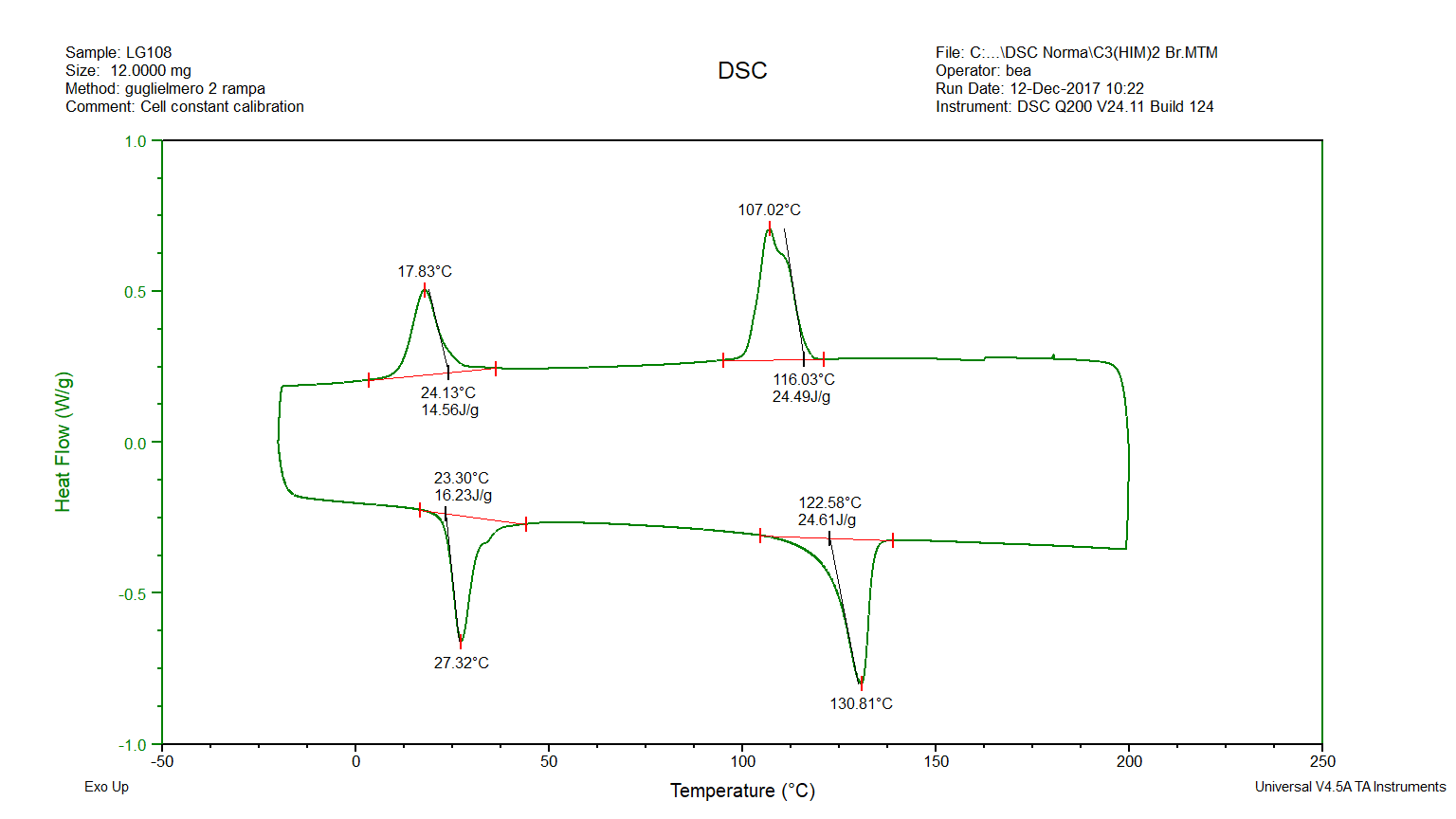


**Figure S39.** Differential scanning calorimetry (DSC) of compounds C_4_(HIM)_2_/2Br (**10**).


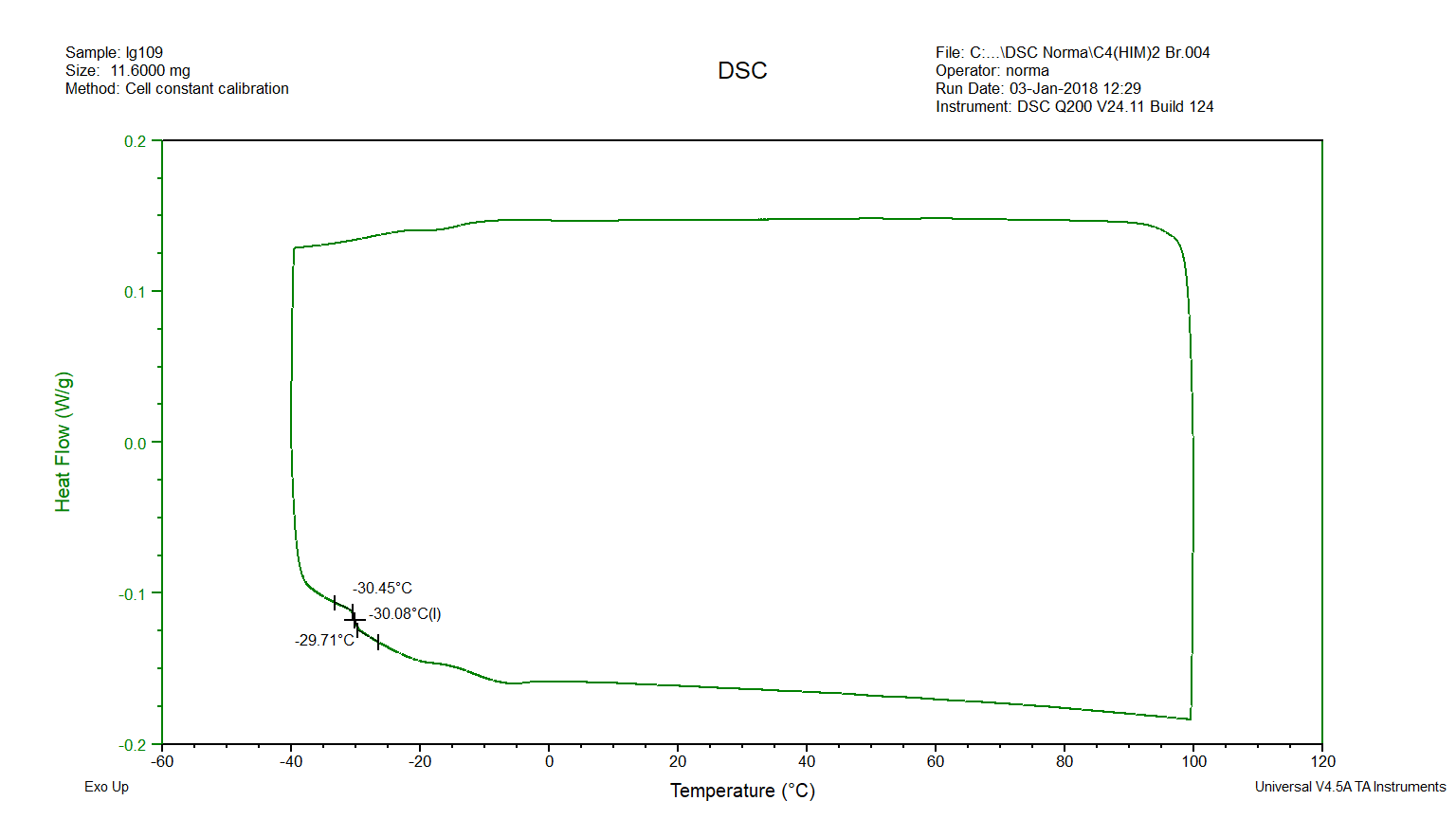


**Figure S40.** Differential scanning calorimetry (DSC) of compounds C_5_(HIM)_2_/2Br (**11**).

**
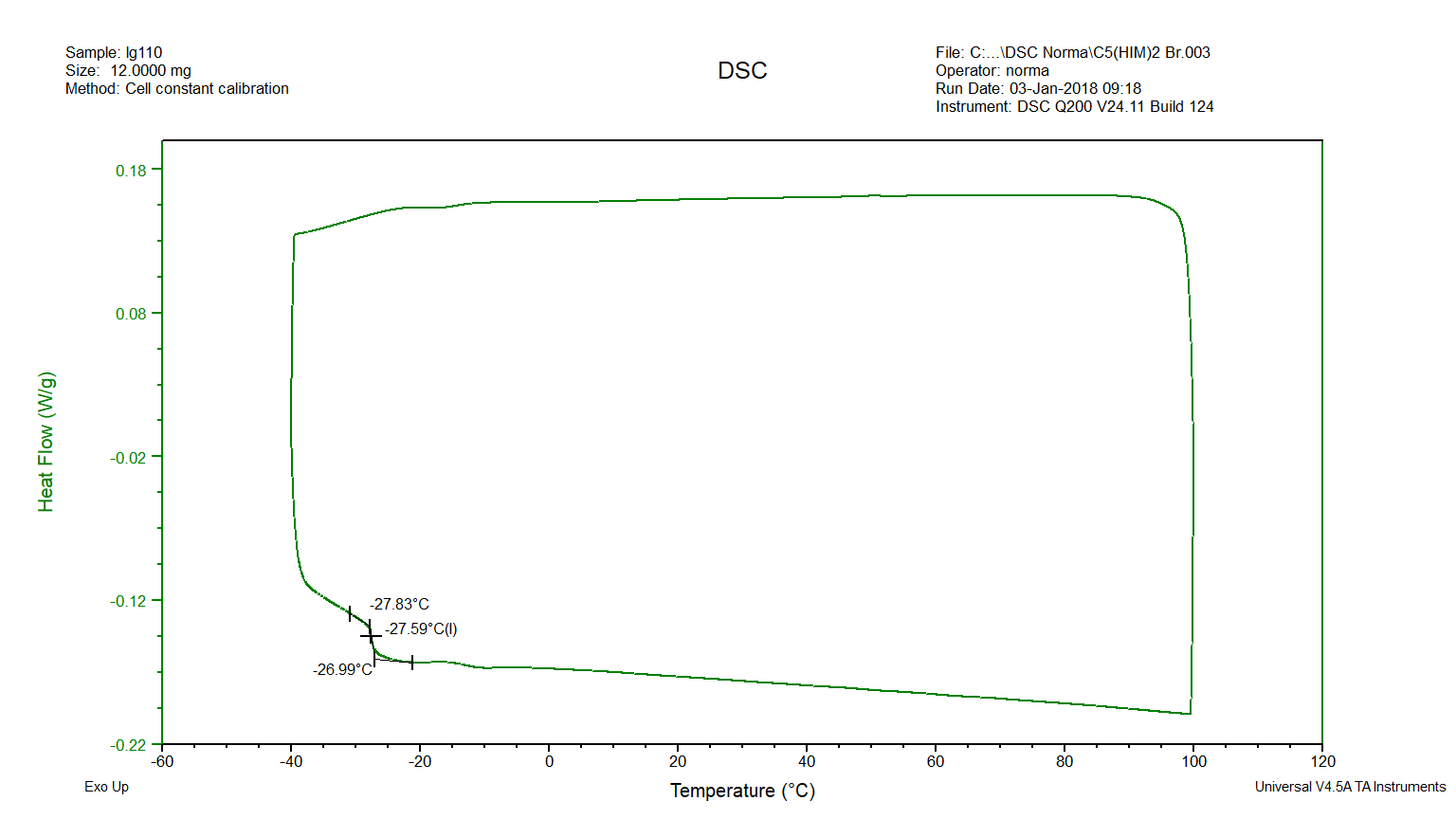
**

**Figure S41.** Differential scanning calorimetry (DSC) of compounds C_6_(HIM)_2_/2Br (**12**).


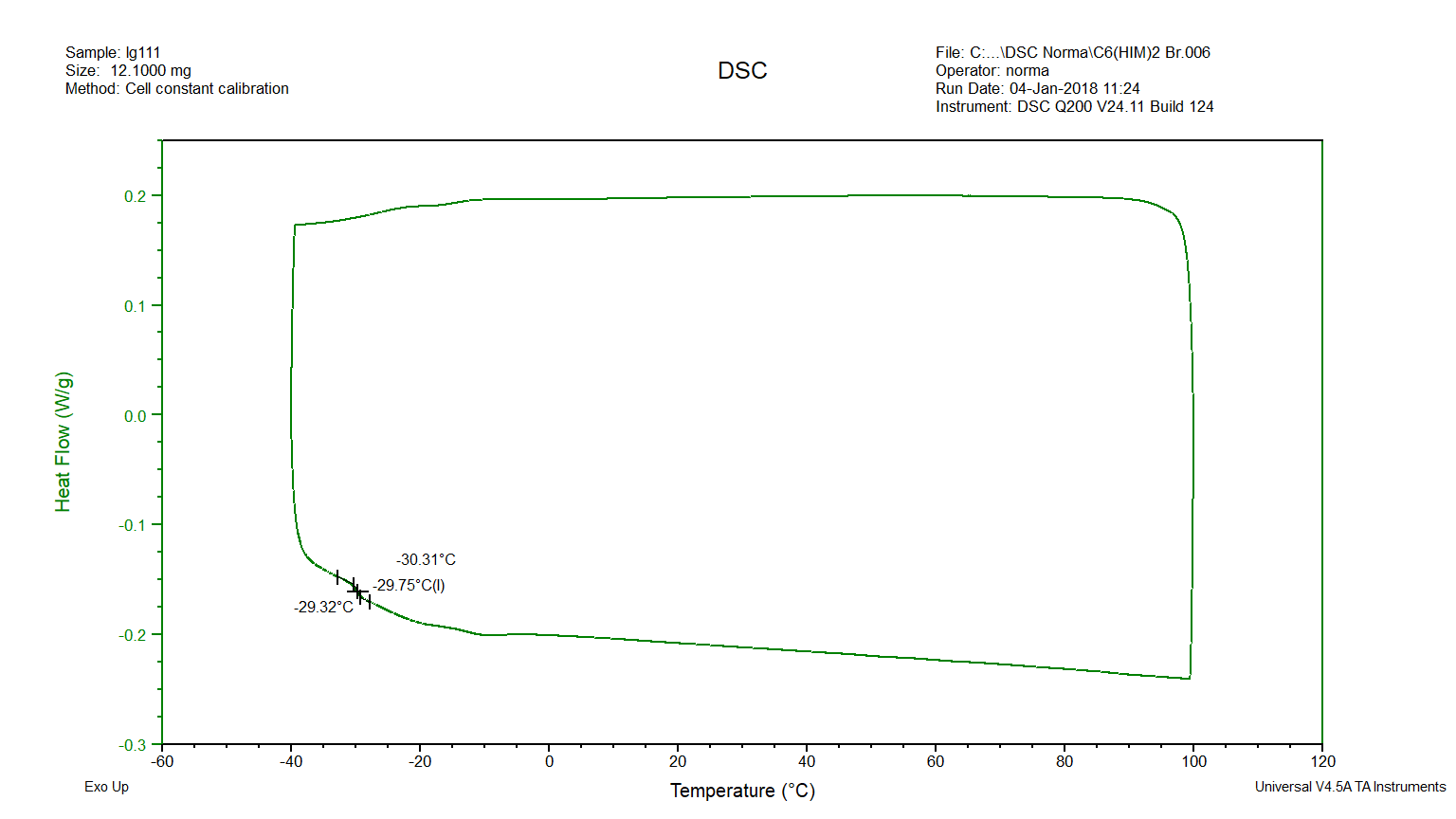


**Figure S42.** DSC and TGA of compound C_3_(MIM)_2_/Mal (**13**).


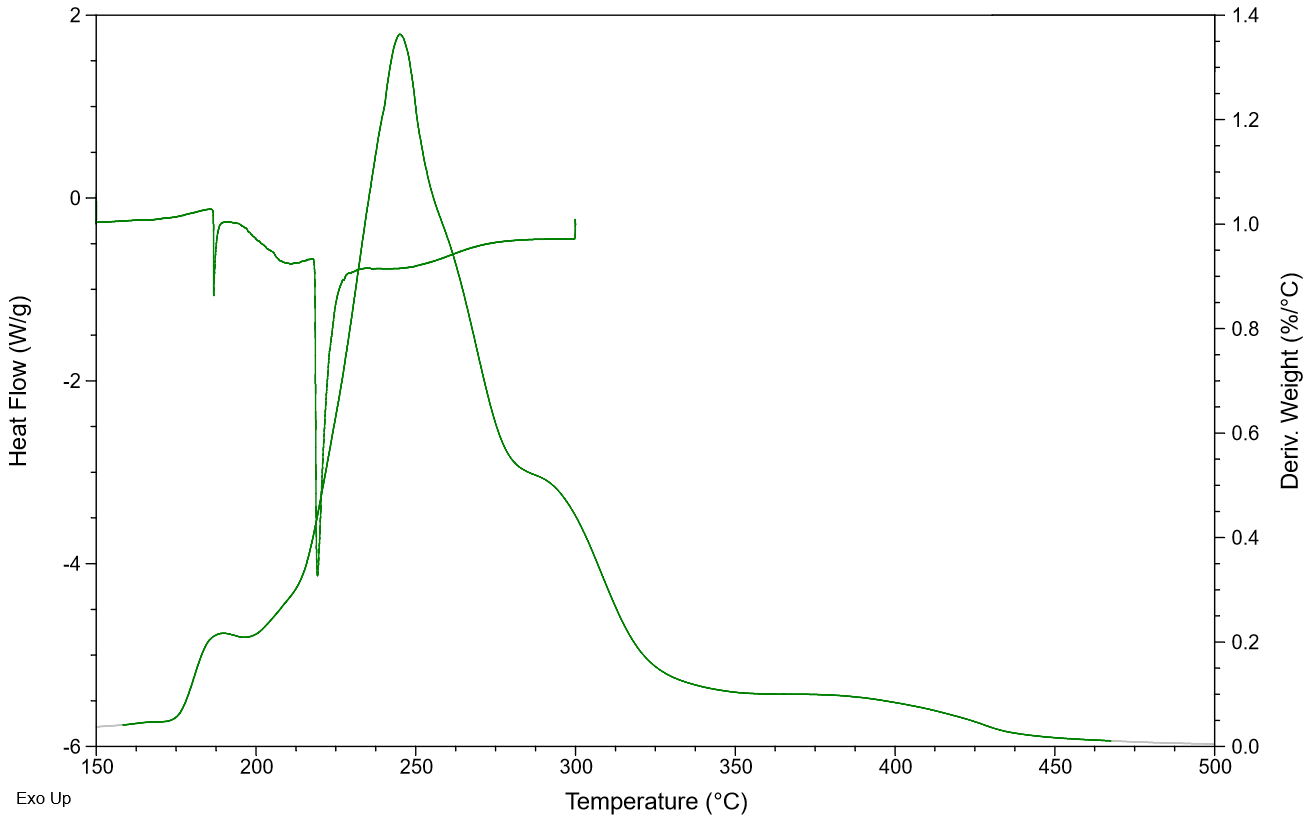


**Figure S43.** DSC and TGA of compound C_4_(MIM)_2_/Mal (**14**).

**
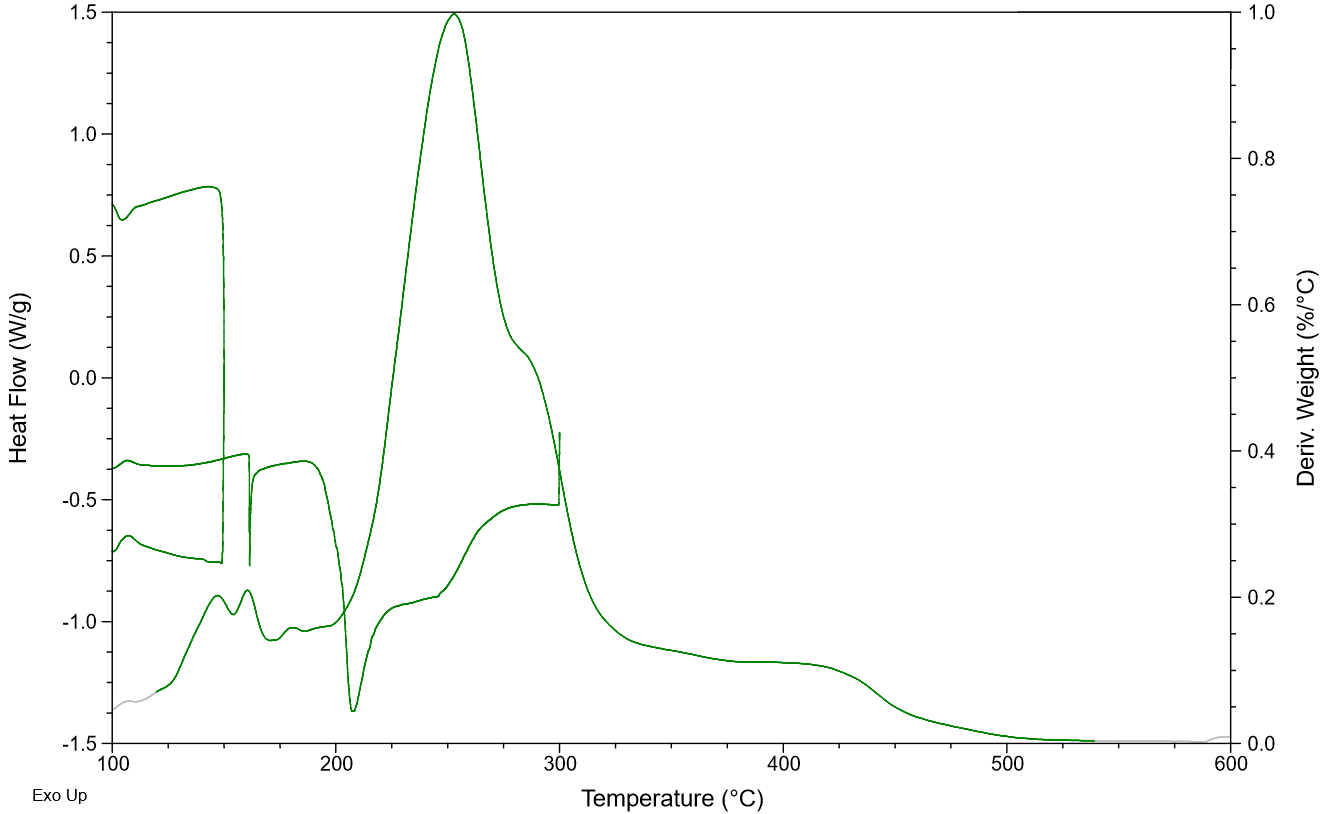
**

**Figure S44.** DSC and TGA of compound C_5_(MIM)_2_/Mal (**15**).

**
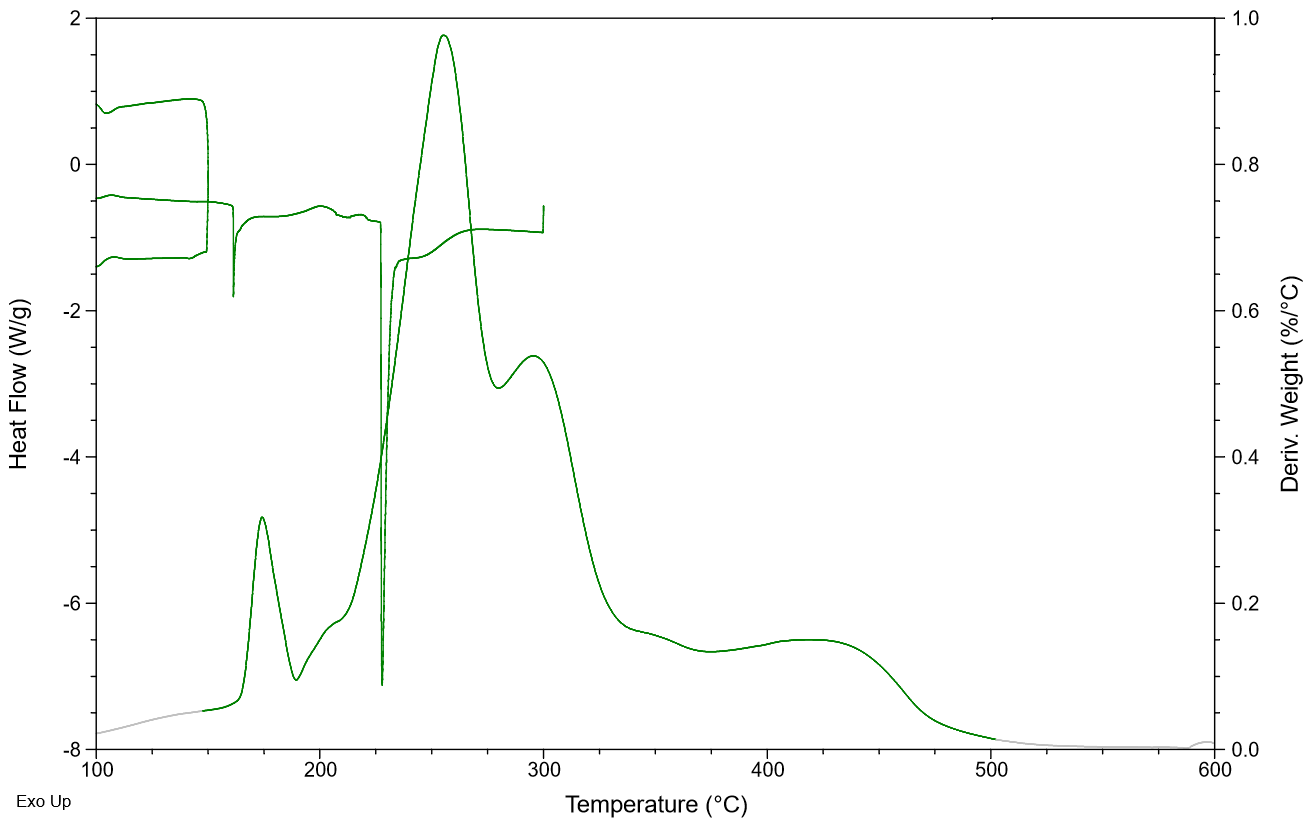
**

**Figure S45.** DSC and TGA of compound C_6_(MIM)_2_/Mal (**16**).

**
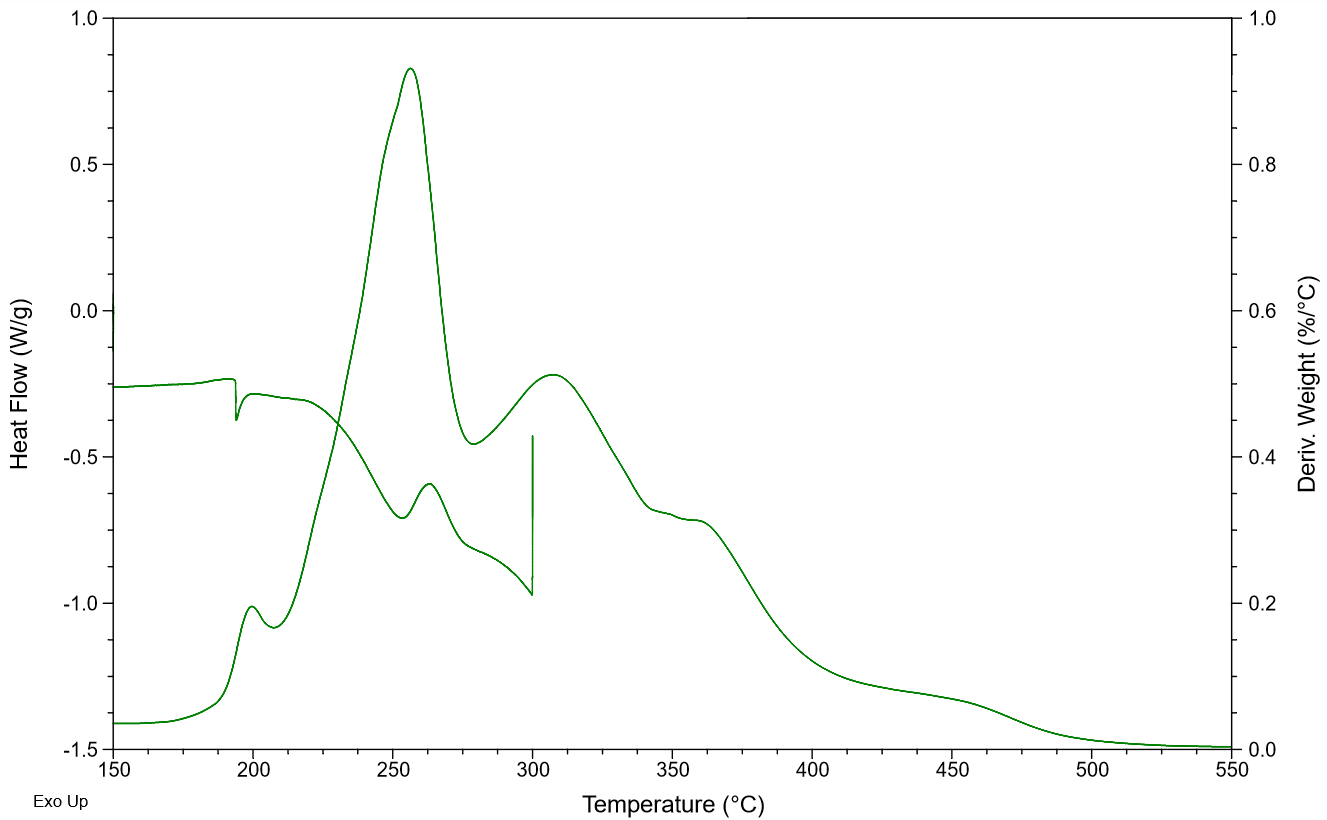
**

**Figure S46.** DSC and TGA of compound C_3_(MIM)_2_/Succ (**17**).

**
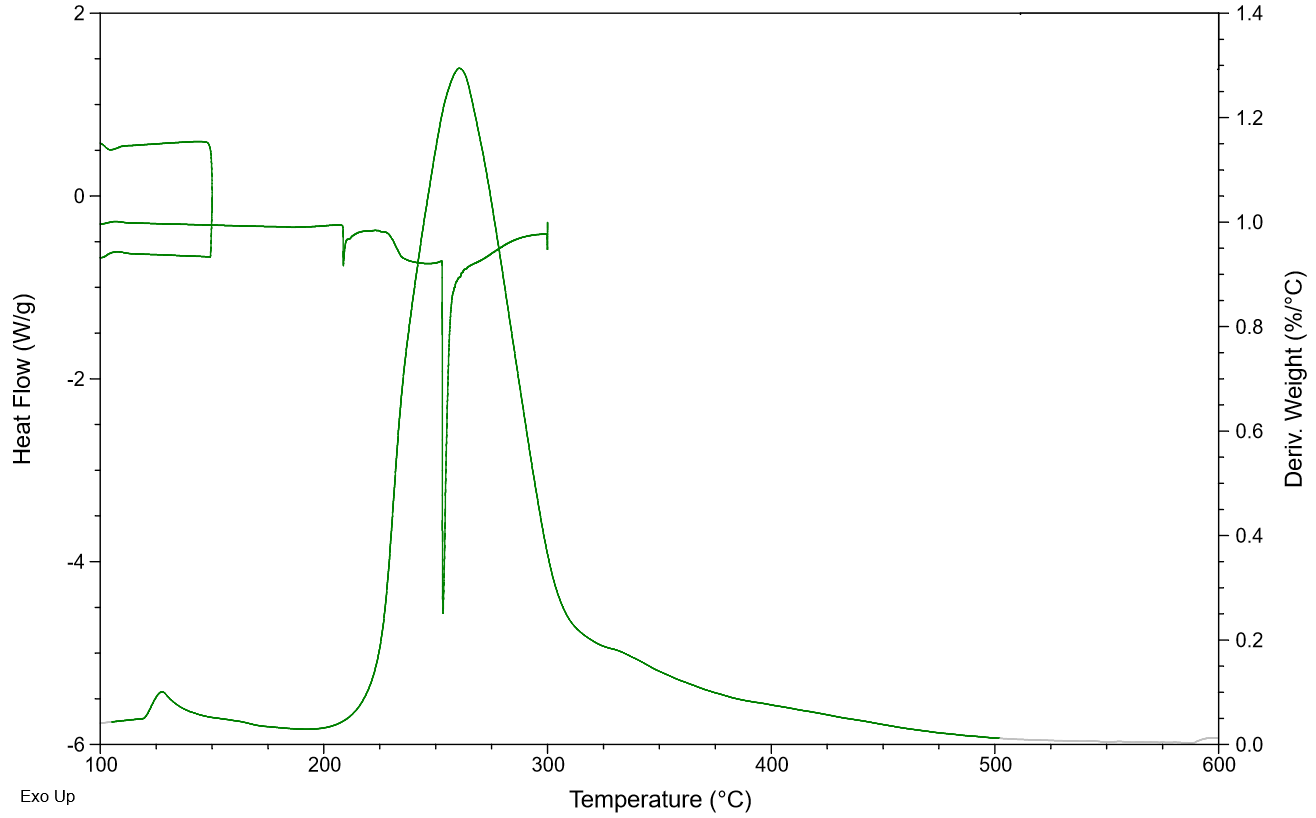
**

**Figure S47.** DSC and TGA of compound C_4_(MIM)_2_/Succ (**18**).

**
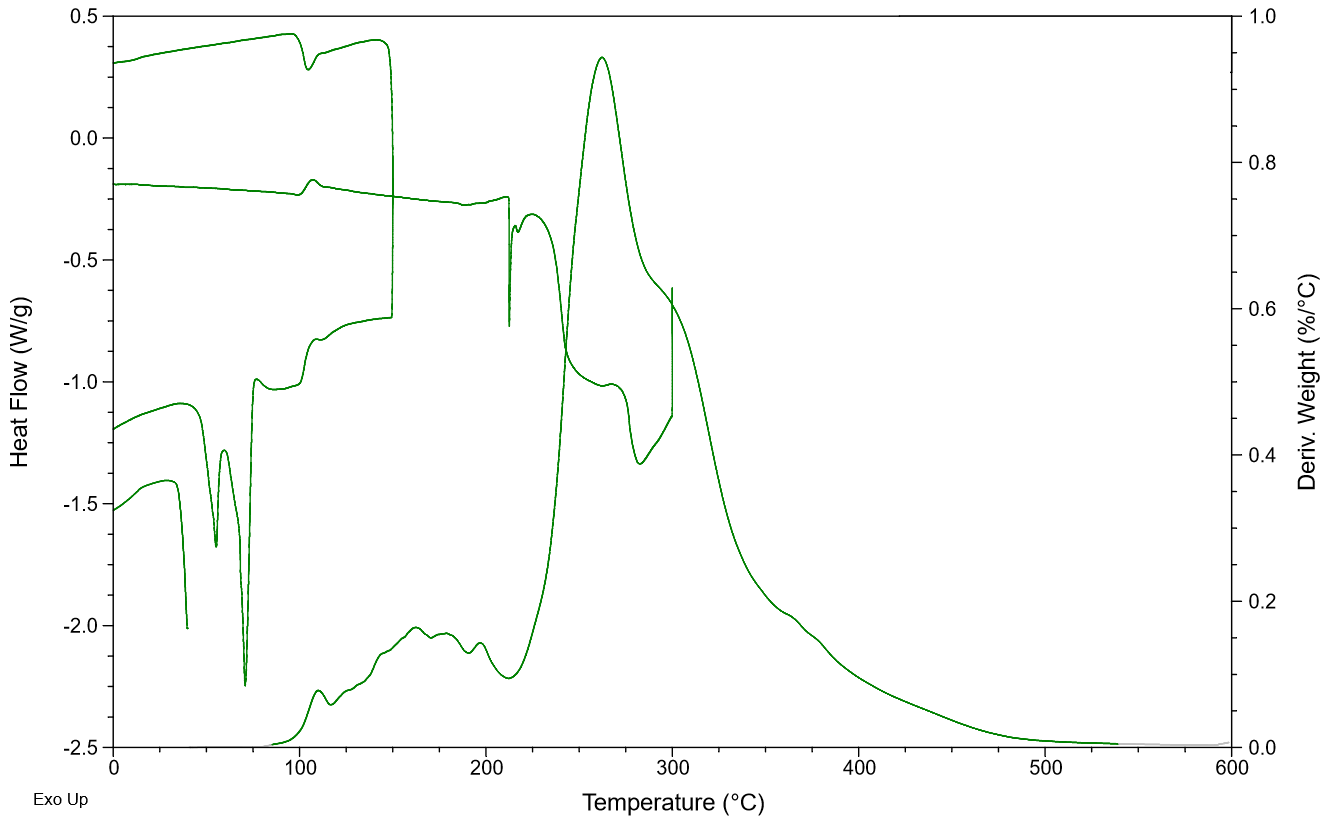
**

**Figure S48.** DSC and TGA of compound C_5_(MIM)_2_/Succ (**19**).


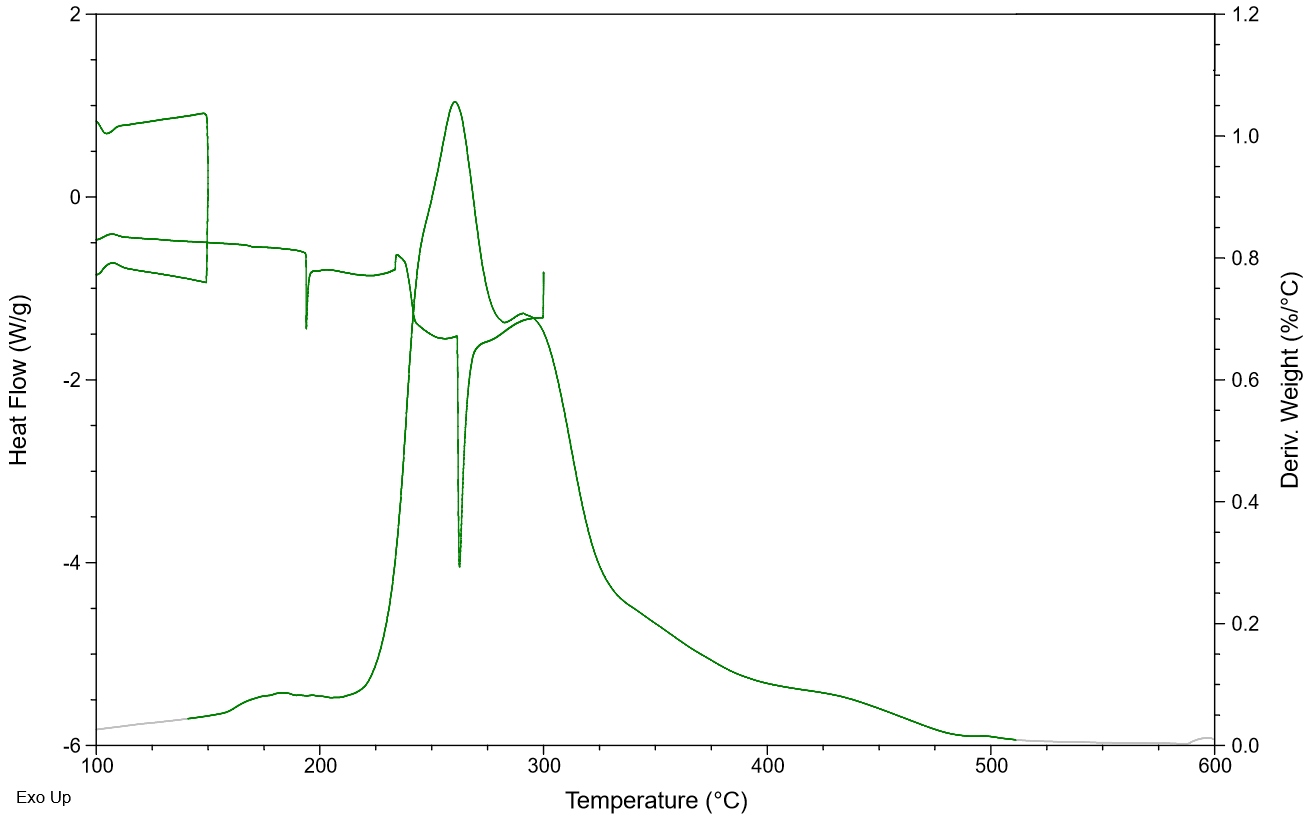


**Figure S49.** DSC and TGA of compound C_6_(MIM)_2_/Succ (**20**).

**
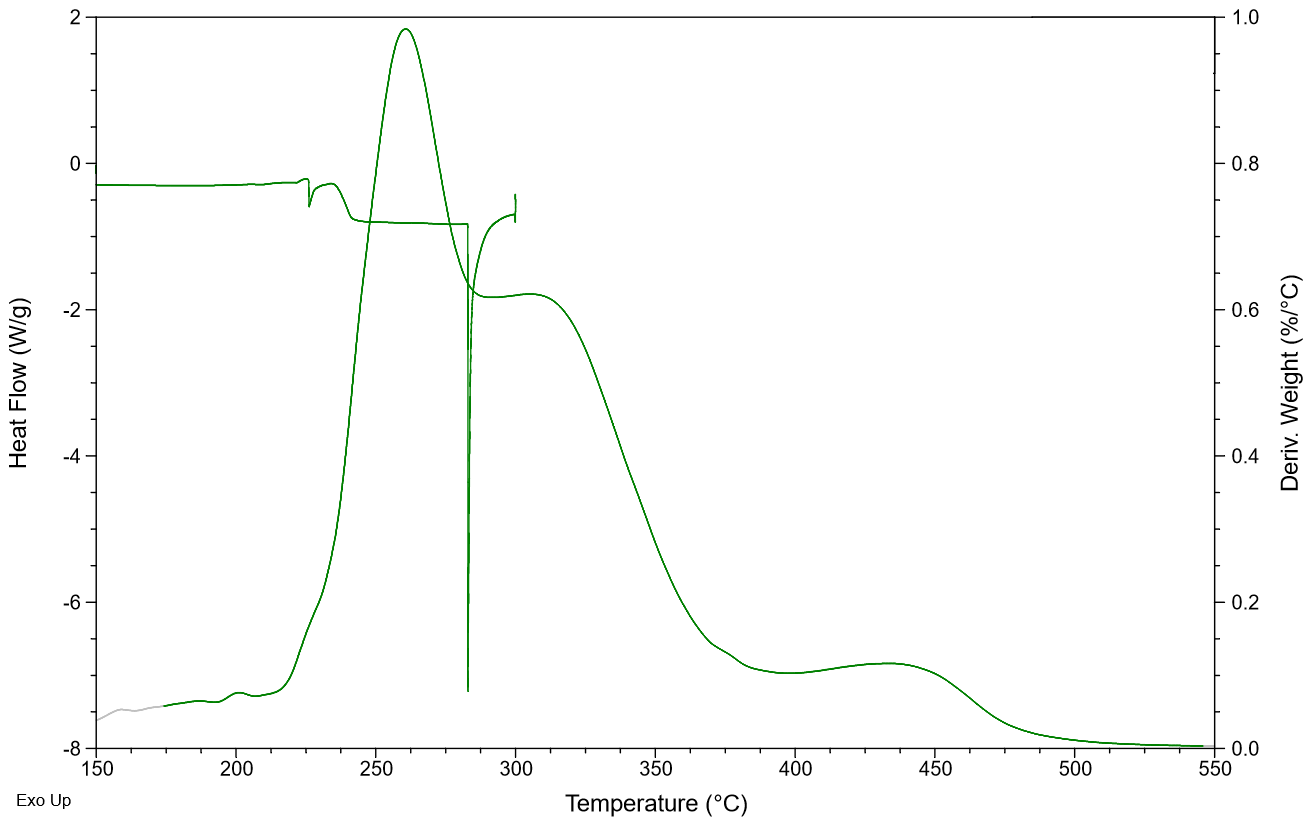
**

**Figure S50.** DSC and TGA of compound C_3_(MIM)_2_/Glut (**21**).


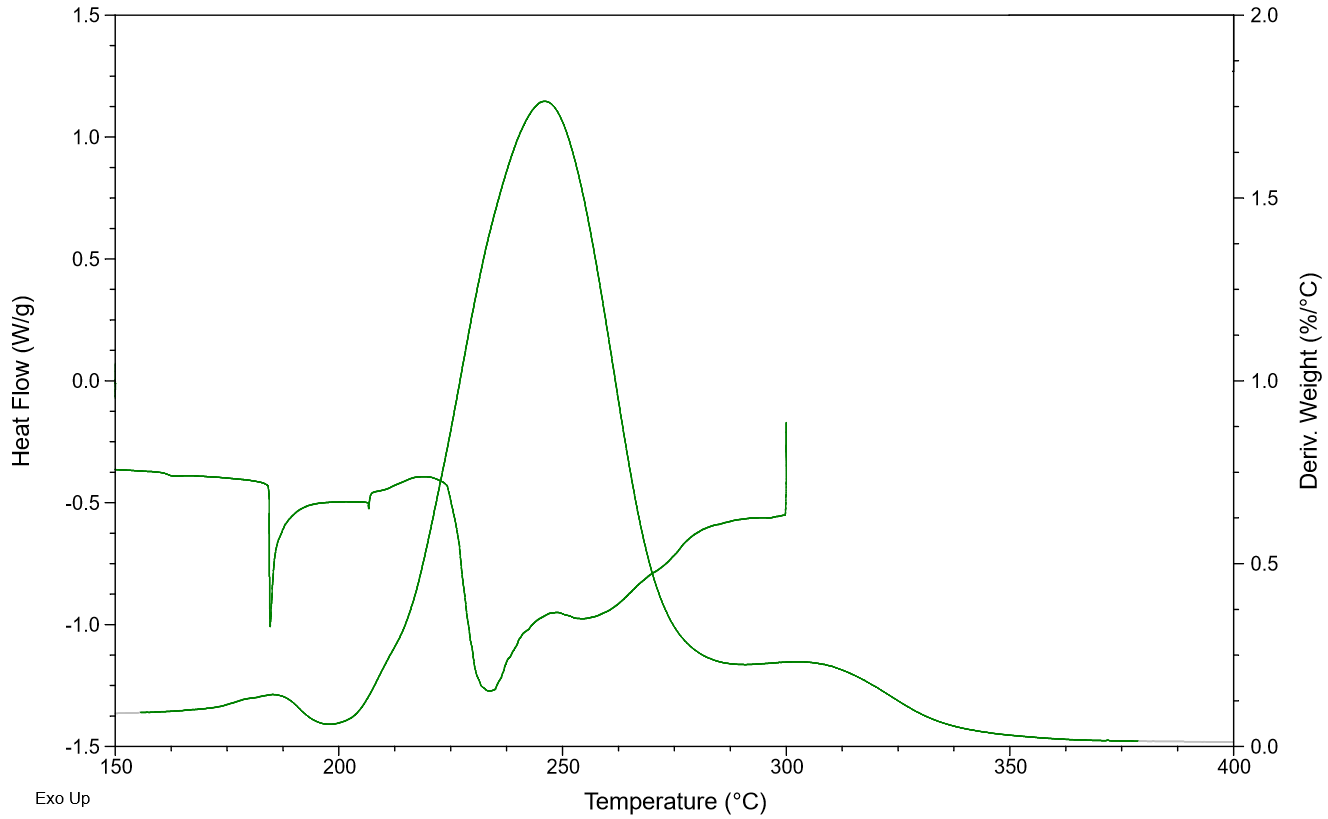


**Figure S51.** DSC and TGA of compound C_4_(MIM)_2_/Glut (**22**).


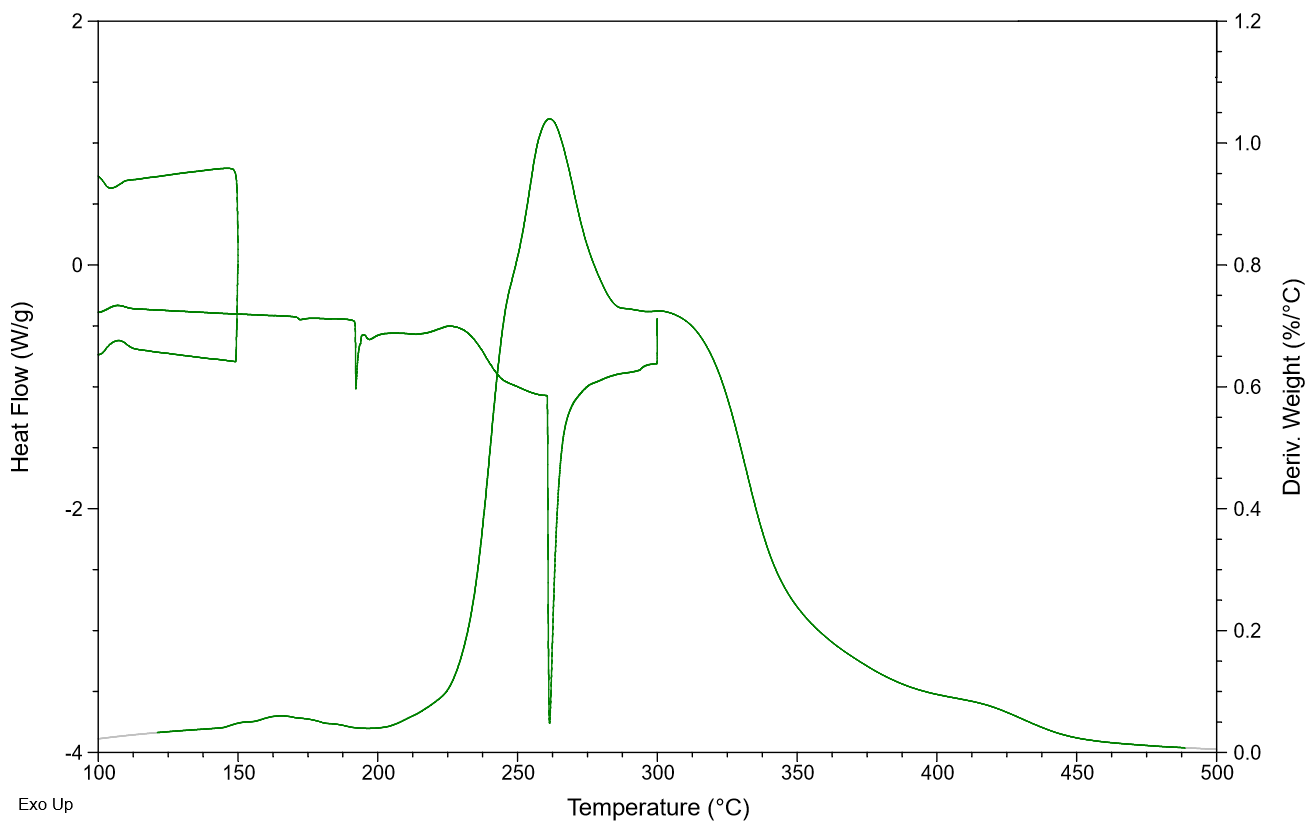


**Figure S52.** DSC and TGA of compound C_5_(MIM)_2_/Glut (**23**).


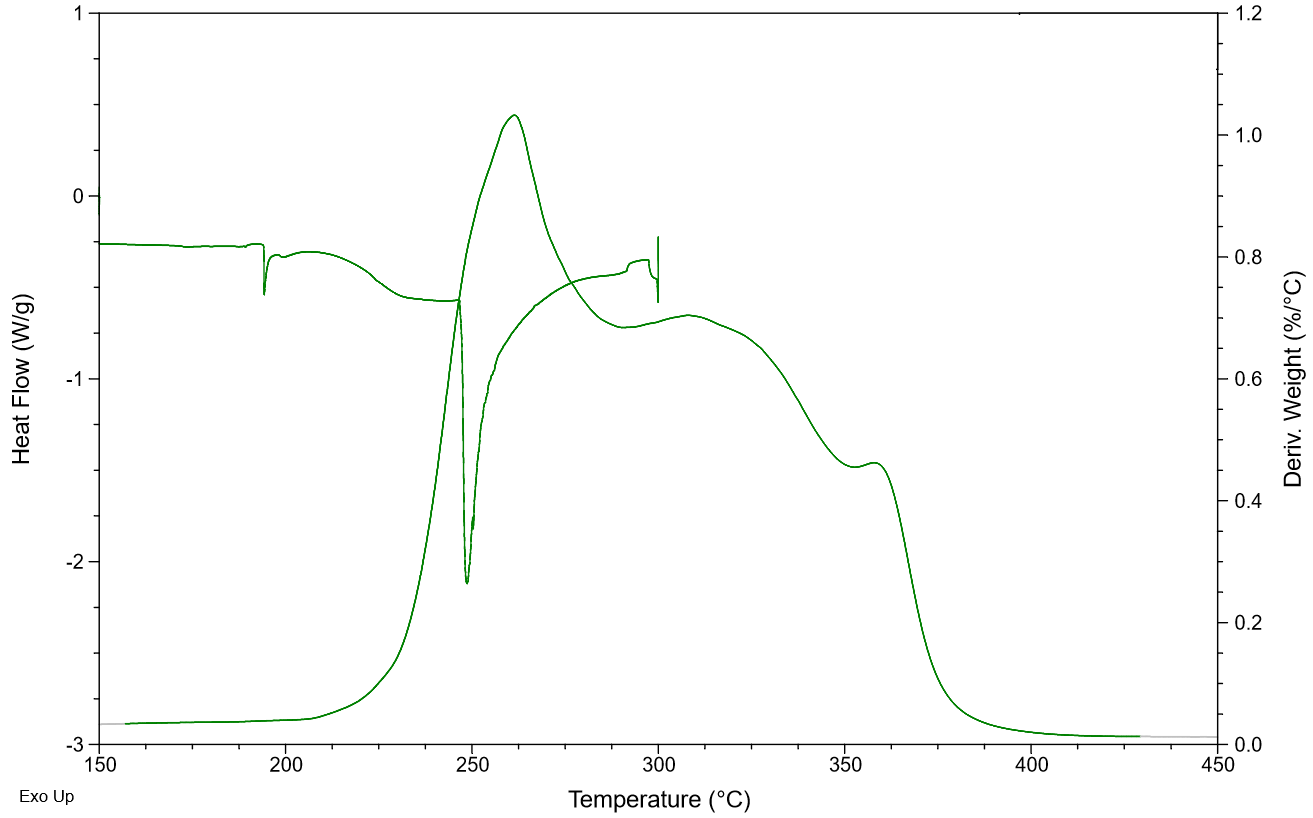


**Figure S53.** DSC and TGA of compound C_6_(MIM)_2_/Glut (**24**).


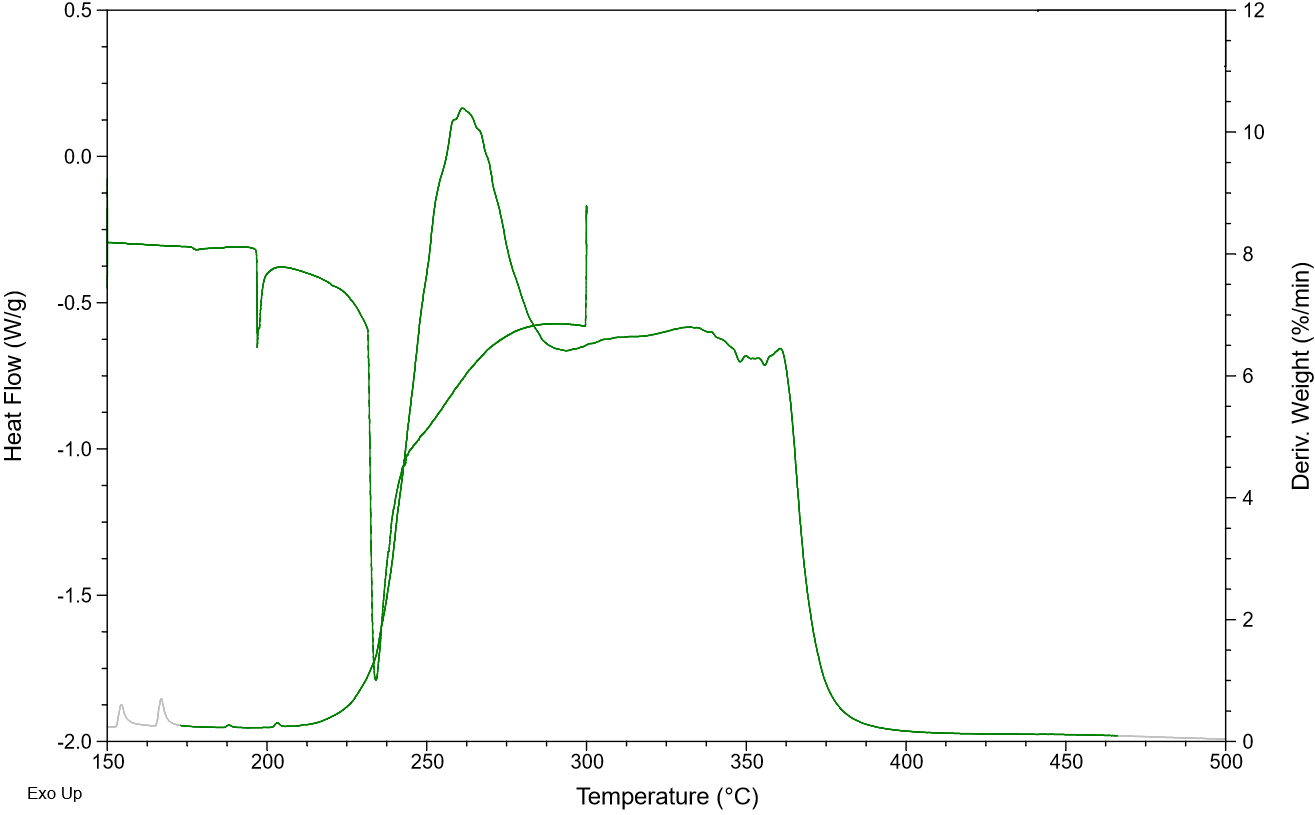


**Figure S54.** ^1^H NMR spectra of Glycerol and C_6_(MIM)_2_/2Br:Glycerol 1:3


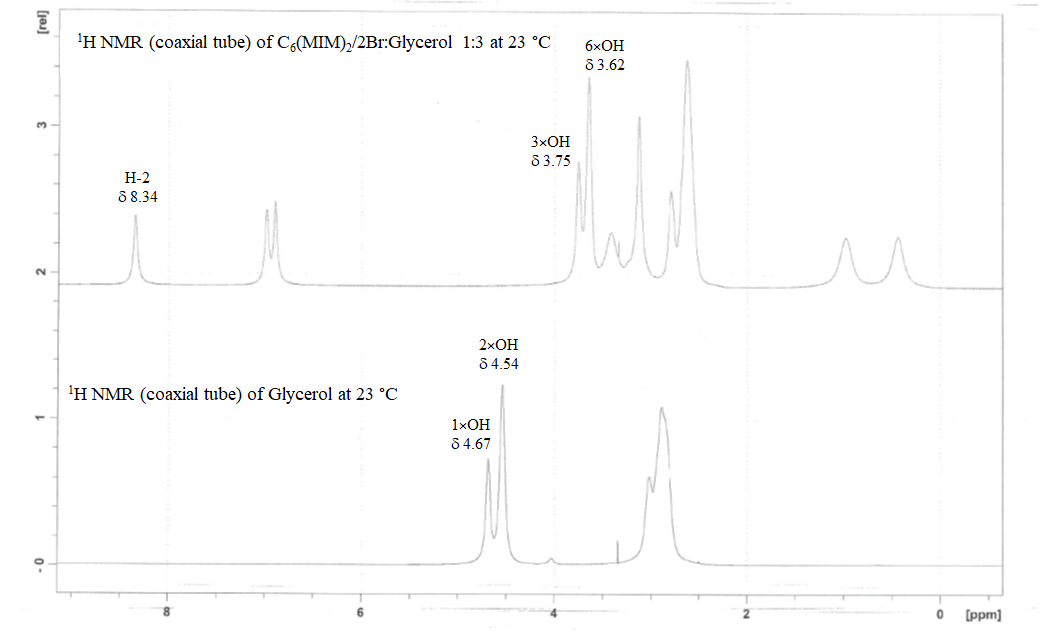


**Figure S55.** ^1^H NMR spectra of Diethylenglycol and C_6_(MIM)_2_/2Br:DiEG 1:6


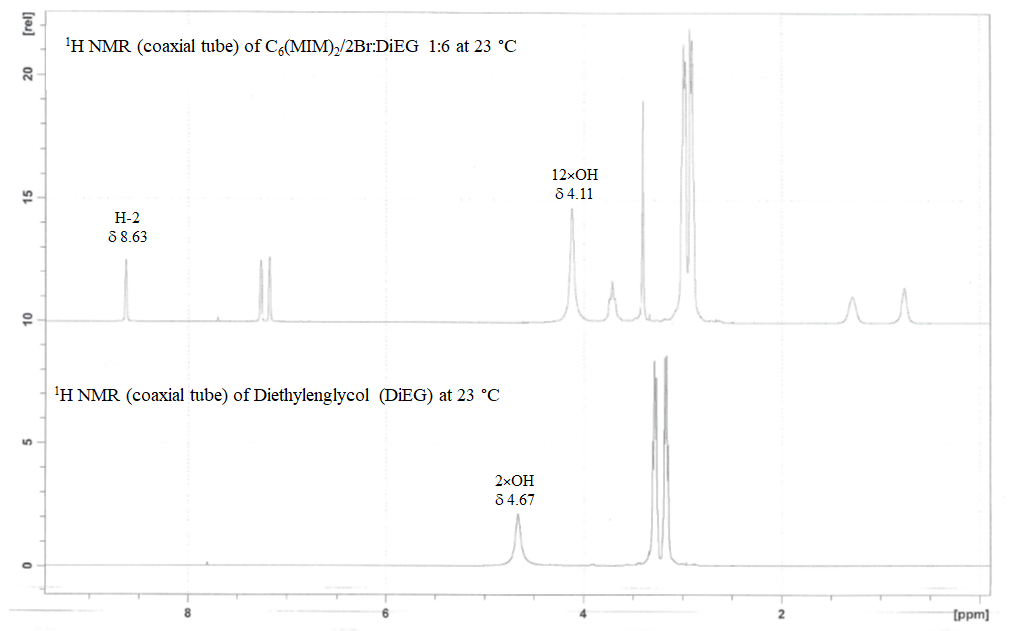


**Figure S56.** ^1^H NMR spectra of Glycerol and C6(MIM)2/Succ:Glycerol 1:2


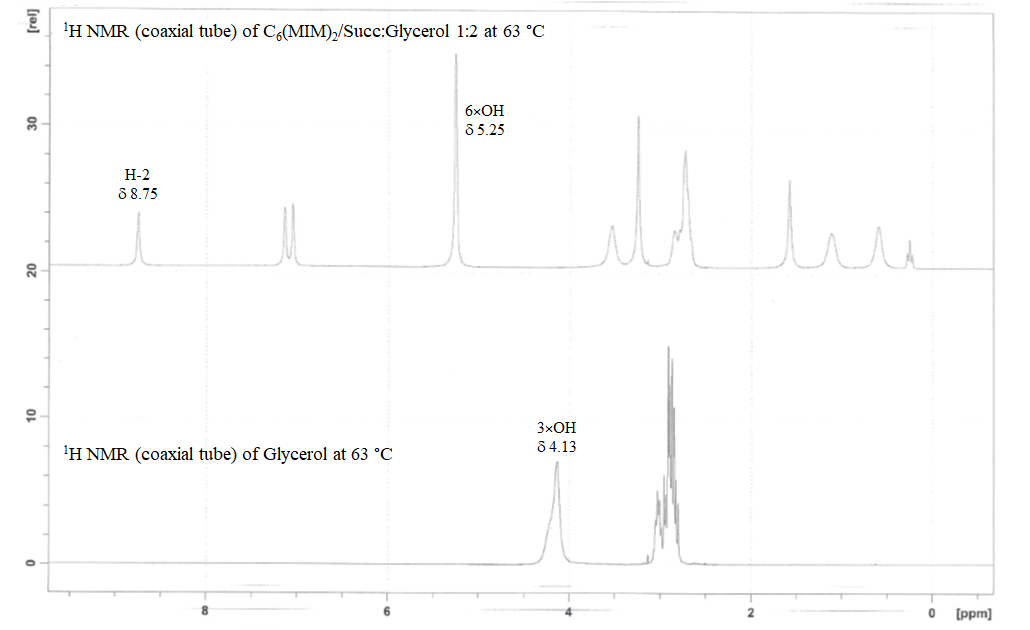


**Figure S57.** ^1^H NMR spectra of Diethylenglycol and C_6_(MIM)_2_/Succ:DiEG 1:2


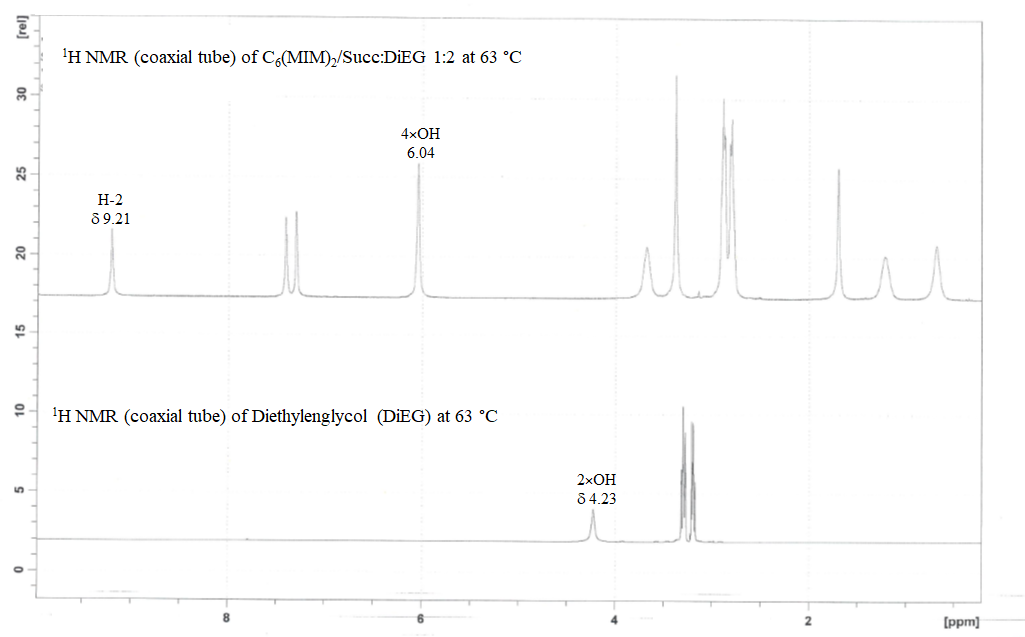

Supplement: Supplementary file 1 [file Data_Sheet_1.docx]
